# A Cullin1-Based SCF E3 Ubiquitin Ligase Targets the InR/PI3K/TOR Pathway to Regulate Neuronal Pruning

**DOI:** 10.1371/journal.pbio.1001657

**Published:** 2013-09-17

**Authors:** Jack Jing Lin Wong, Song Li, Edwin Kok Hao Lim, Yan Wang, Cheng Wang, Heng Zhang, Daniel Kirilly, Chunlai Wu, Yih-Cherng Liou, Hongyan Wang, Fengwei Yu

**Affiliations:** 1 Temasek Life Sciences Laboratory and Department of Biological Sciences, National University of Singapore, Singapore; 2 Graduate School for Integrated Sciences and Engineering, Centre for Life Sciences, National University of Singapore (NUS), Singapore; 3 Neuroscience and Behavioral Disorder Program, Duke–NUS Graduate Medical School Singapore, Singapore; 4 Neuroscience Center of Excellence, Louisiana State University Health Sciences Center, New Orleans, Louisiana, United States of America; 5 Department of Physiology, Yong Loo Lin School of Medicine, National University of Singapore, Singapore; 6 Department of Biochemistry, Yong Loo Lin School of Medicine, National University of Singapore, Singapore; Baylor College of Medicine, United States of America

## Abstract

Pruning that selectively eliminates unnecessary axons/dendrites is crucial for sculpting the nervous system during development. During *Drosophila* metamorphosis, dendrite arborization neurons, ddaCs, selectively prune their larval dendrites in response to the steroid hormone ecdysone, whereas mushroom body γ neurons specifically eliminate their axon branches within dorsal and medial lobes. However, it is unknown which E3 ligase directs these two modes of pruning. Here, we identified a conserved SCF E3 ubiquitin ligase that plays a critical role in pruning of both ddaC dendrites and mushroom body γ axons. The SCF E3 ligase consists of four core components Cullin1/Roc1a/SkpA/Slimb and promotes ddaC dendrite pruning downstream of EcR-B1 and Sox14, but independently of Mical. Moreover, we demonstrate that the Cullin1-based E3 ligase facilitates ddaC dendrite pruning primarily through inactivation of the InR/PI3K/TOR pathway. We show that the F-box protein Slimb forms a complex with Akt, an activator of the InR/PI3K/TOR pathway, and promotes Akt ubiquitination. Activation of the InR/PI3K/TOR pathway is sufficient to inhibit ddaC dendrite pruning. Thus, our findings provide a novel link between the E3 ligase and the InR/PI3K/TOR pathway during dendrite pruning.

## Author Summary

Neurons have the ability to engage in selective pruning that eliminates unnecessary axons/dendrites. This process is crucial for sculpting the nervous system during development. During *Drosophila* development, dendrite arborization sensory neurons (ddaCs) selectively prune their larval dendrites in response to the molting steroid hormone ecdysone, whereas mushroom body γ neurons eliminate their axon branches. However, the underlying molecular mechanisms for both of these modes of pruning were not well understood. Here, we conduct a genome-wide screen and identify a conserved E3 ubiquitin ligase that is critical for pruning both ddaC dendrites and mushroom body γ axons. This ligase complex has four core components—Cullin1, Roc1a, SkpA, and Slimb—that promote ddaC dendrite pruning in response to ecdysone. We show that this ligase facilitates ddaC dendrite pruning through regulation of the InR/PI3K/TOR pathway. The substrate-recognition protein Slimb promotes ubiquitination of Akt, an activator of the InR/PI3K/TOR pathway. Akt ubiquitination leads to its degradation and inactivation of the InR/PI3K/TOR pathway, which is required for dendritic pruning. Consistent with this, ddaC dendrite pruning is inhibited when the InR/PI3K/TOR pathway is activated. Thus, we identify a link between the Cullin1-based E3 ligase and the InR/PI3K/TOR pathway in regulating dendrite pruning. This work represents the first link between neuronal pruning and the insulin signaling pathway, raising interesting questions about how metabolic states may influence the control of such developmental processes.

## Introduction

The selective removal of unnecessary or exuberant neuronal processes without loss of neurons, referred to as pruning, is a central theme in the maturation of the nervous system during animal development [1]. Pruning occurs widely in a variety of neurons of invertebrates [2],[3] and vertebrates [4]. In vertebrates, neurons normally extend exuberant branches to multiple targets, such as muscles or partner neurons, and prune away inappropriate or redundant branches to develop mature and functional connectivity [1],[5]. One well-characterized example is pervasive synaptic branch removal in the mammalian neuromuscular system at birth [6]. In invertebrates, such as holometabolous insects *Manduca* and *Drosophila*, the nervous systems are extensively remodeled via pruning and apoptosis during metamorphosis, a transition stage between larval and adult forms [2],[3]. In the *Drosophila* central nervous system (CNS), mushroom body (MB) γ neurons, serotonergic neurons, and thoracic ventral neurons prune their larval dendrites and/or axons to form the adult neuronal circuits [7]–[10]. In contrast, in the peripheral nervous system (PNS), a subset of dorsal dendrite arborization (dda) sensory neurons, such as Class I (ddaD/E) and class IV (ddaC) neurons, selectively remove their larval dendrite arbors with their axons intact and subsequently regrow their adult-specific dendrites [11],[12], whereas class II (ddaB) and class III (ddaA/F) neurons are eliminated via apoptosis during early metamorphosis [11]. Despite the wide occurrence and key roles of pruning in the maturing nervous systems, the molecular and cellular mechanisms underlying pruning remain poorly understood in both invertebrates and vertebrates.

**Figure 1:**
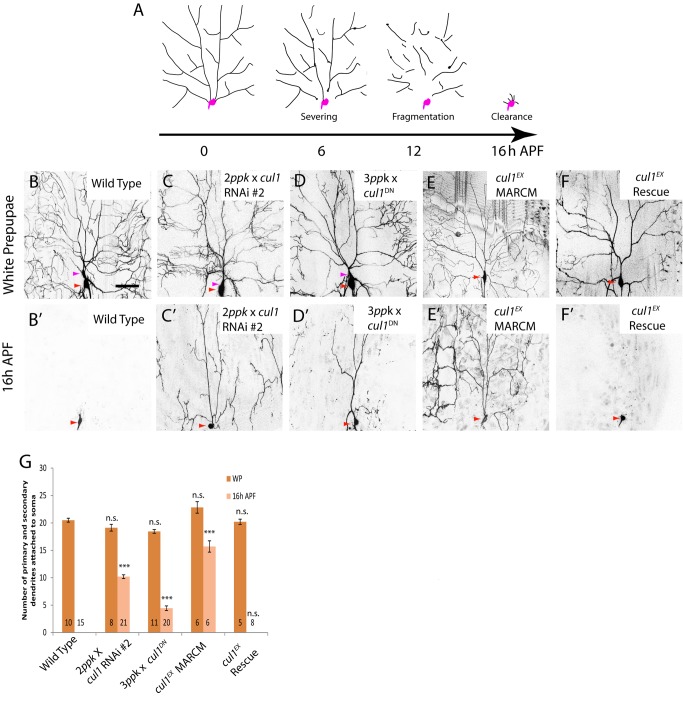
Cullin-1, a core component of the SCF E3 ligase, is required for pruning of ddaC dendrites. (A) A schematic representation of dendrite pruning in ddaC neurons. Soma and axon are shown in purple while dendrites are in black. (B–F′) Live confocal images of ddaC neurons expressing *UAS-mCD8-GFP* driven by *ppk*-*Gal4* at WP and 16 h APF. Red arrowheads point to the ddaC somas, and purple arrowheads point to the ddaF somas that are labeled by two or three copies of *ppk-Gal4* (Chr II). While the wild-type neurons eliminated dendrites (B, B′), ddaC neurons overexpressing two copies of *cul1* RNAi #2 (C, C′), three copies of *cul1^DN^* (D, D′), or *cul1^EX^* MARCM (E, E′) ddaC clones exhibited severe dendrite pruning defects at 16 h APF. Overexpression of the full-length Cul1 protein in the *cul1^EX^* background was able to fully rescue the pruning defect (F, F′). (G) Quantification of the average number of primary and secondary dendrites attached to the soma of the wild-type and mutant ddaC neurons at WP stage and 16 h APF. The number of samples (*n*) in each group is shown on the bars. Error bars represent S.E.M. ***p<0.001, n.s., not significant. Dorsal is up in all images. The scale bars are 50 µm. See genotypes in Text S1.

**Figure 2:**
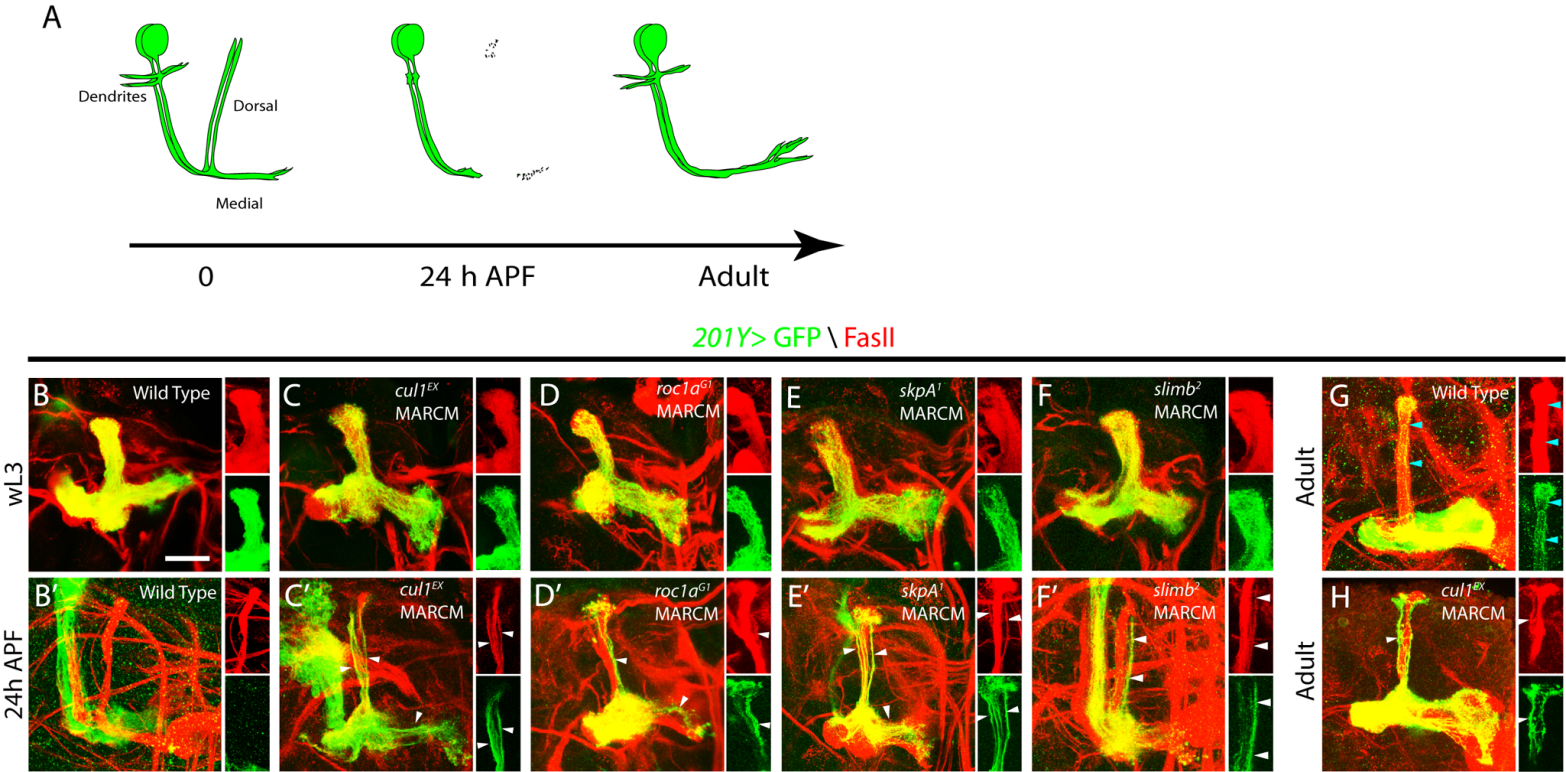
The Cul1-based SCF E3 ligase components are required for MB axon pruning. (A) A schematic representation of axon pruning in the medial and dorsal lobes of MB γ neurons. (B–H) Confocal images of MB neurons expressing *UAS-mCD8-GFP* driven by *201Y*-*Gal4* at wL3, 24 h APF, or adulthood. *201Y-Gal4* labels postmitotic γ neurons and a small subset of later-born α/β neurons. The Anti-FasII (1D4) antibody labels α/β neurons strongly and γ neurons weakly. (B) Wild-type MB γ neurons projected their axons into the dorsal and medial lobe of the brain at wL3. (C–F) *cul1^EX^* (C), *roc1a^G1^* (D), *skpA^1^* (E), and *slimb^2^* (F) neuroblast clones that were labeled with *201Y-Gal4* projected their dorsal and medial axons normally but exhibited proliferation defects at wL3, as the axon branches were less dense than the wild-type control. (B′) Wild-type MB γ neurons pruned their dorsal and medial axon branches by 24 h APF, leaving intact α/β axons (GFP negative and FasII positive). (C′–F′) *cul1^EX^* (C′), *roc1a^G1^* (D′), *skpA^1^* (E′), and *slimb^2^* (F′) MB neuroblast clones displayed notable axon pruning defects at 24 h APF. The right panels in (C′–F′) show that many unpruned dorsal γ axons persisted outside the main α lobes and were co-labeled by GFP and FasII (white arrowheads) in single confocal sections of the dorsal lobe. (G) At adulthood, MB γ neurons regenerated their medial branches but not the dorsal branches. Blue arrowheads point to α axon branches. (H) *cul1^EX^* MB neuroblast clones retained many GFP-positive unpruned larval γ axons (white arrowheads) outside the FasII-positive main α lobe at the adult stage. However, the GFP-positive homozygous *cul1^EX^* mutant α axon branches were absent in this main α lobe (H), suggesting a proliferation defect. Please note that the FasII-positive α lobe is derived from the other heterozygous MB neuroblasts. Dorsal is up in all images. The scale bar is 50 µm. See genotypes in Text S1.

*Drosophila* MB γ and ddaC neurons have been emerging as appealing systems for unraveling mechanisms of axon pruning and dendrite pruning, respectively. These pruning processes occur in a stereotyped but context-specific manner: ddaC neurons sever their major larval dendrites at the proximal regions, followed by rapid fragmentation and hemocyte-dependent debris clearance (Figure 1A) [11],[12], whereas MB γ neurons selectively prune their axon branches within the dorsal and medial lobes via local degeneration and glia-mediated engulfment (Figure 2A) [7],[13]–[15]. Pruning of the *Drosophila* nervous system is regulated by the steroid molting hormone 20-hydroxyecdysone (ecdysone) and its nuclear receptor heterodimer (EcR-B1/Usp) [16]. Ecdysone binds to a nuclear receptor heterodimer consisting of EcR-B1 and its co-receptor Ultraspiracle (Usp) to trigger the onset of pruning by activating the downstream pruning programs, rather than the conventional ecdysone response genes *Broad complex*, *E74*, and *E75* [7]. The ecdysone-induced pruning programs are activated via a multilayered regulatory process. First, in response to a late larval pulse of ecdysone, EcR-B1 expression is upregulated, depending on TGF-β signaling [17],[18], the cohesin complex [19],[20], and the Ftz-F1/Hr39 nuclear receptors [21]. Second, EcR-B1, together with two epigenetic factors, Brahma and CREB-binding protein, activates the expression of their common target gene *sox14* [22]. Third, Sox14, a key transcription factor, in turn induces the expression of the cytoskeletal regulator Mical [23]. Interestingly, despite its elevated expression in ddaC and MB γ neurons, Mical is only required for ddaC dendrite pruning but dispensable for MB axon pruning [23]. Aside from Mical, other molecules, such as caspases [24],[25], Ik2 and Kat60L [26], are also specifically required for ddaC dendrite pruning. Therefore, it remains poorly understood what common pruning machinery directs two distinct modes of pruning in ddaC and MB γ neurons.

The ubiquitin-proteasome system (UPS) is a potent cellular pathway regulating protein stability and homeostasis in eukaryotic cells [27]. Growing evidence indicates that the UPS pathway plays critical roles in neuronal development [28] and neuro-disorders [29]. Pharmacological inhibition of the UPS activity leads to apparent retardation of injury-induced axon degeneration [30]. In the UPS pathway, the polypeptide ubiquitin is activated by a ubiquitin-activating enzyme E1; the activated ubiquitin is transferred to a ubiquitin-conjugating enzyme E2 and then, via a ubiquitin ligase E3, to protein substrates, destined for 26S proteasome-mediated degradation. Substrate specificity is conferred by different classes of E3 ligases, one of which contains RING (really interesting new gene) domain. An important RING E3 ligase is the SCF (Skp1-Cullin-F-box) E3 ligase composed of the adaptor Skp1, the scaffold protein Cullin, the RING-domain protein Roc1-Rbx1-Hrt1, as well as the F-box protein for substrate recognition [31]. The conserved SCF E3 ligase complex containing the RPM-1/Highwire RING protein negatively regulates the MAP kinase signaling or the receptor tyrosine kinase ALK to control synapse formation and homeostasis in *C. elegans* and *Drosophila* [32]–[34]. Another worm SEL-10 SCF E3 ligase, whose activity is spatially restricted, directs selective synapse pruning, presumably by locally disrupting as-yet-unidentified substrates or pathways [35]. In *Drosophila*, inactivation of the UPS pathway by removing the proteasome subunits, the E1 activating enzyme Uba1, or expressing the yeast ubiquitin protease UBP2 blocks both MB γ axon pruning [13] and ddaC dendrite pruning [12]. Autoubiquitination and degradation of the caspase-antagonizing RING E3 ligase, *Drosophila* inhibitor of apoptosis protein 1 (DIAP1), allows for local activation of the Dronc caspase and thereby dendrite pruning of ddaC neurons [24]. However, DIAP1 (Wong and Yu, unpublished data) and Dronc [24] are not involved in axon pruning of MB γ neurons, raising the intriguing questions of whether and which E3 ligase complex directs two distinct modes of pruning in ddaC and MB γ neurons. Moreover, it is also of great interest to understand which downstream pathway is inactivated by the E3 ligase in order to facilitate the pruning process in ddaC neurons during early metamorphosis.

Here, we identified an E3 ubiquitin ligase complex that plays a crucial role in directing two distinct modes of pruning in ddaC and MB γ neurons during early metamorphosis. In a genome-wide RNA interference (RNAi) screen, we isolated Cullin1 (Cul1), a core scaffold protein of the SCF E3 ligase, which governs pruning of ddaC dendrites and MB axons. Additional components of the Cul1-based SCF E3 ligase include the RING domain protein Roc1a, the adaptor protein SkpA, and the F-box protein Slimb, all of which are essential for ddaC dendrite pruning and MB γ axon pruning. Cul1 acts together with Roc1a, SkpA, and Slimb to promote ddaC dendrite pruning downstream of EcR-B1 and Sox14, but independently of Mical. Furthermore, the Cul1-based SCF E3 ligase promotes ddaC dendrite pruning through negative regulation of the InR/PI3K/TOR signaling pathway but not its conventional target pathways, such as the Hedgehog (Hh) and Wingless (Wg) pathways [36]. We show that attenuation of the InR/PI3K/TOR pathway leads to strong suppression of dendrite pruning defects in mutant ddaC neurons deficient in the Cul1 SCF ligase, whereas activation of the InR/PI3K/TOR pathway alone is sufficient to inhibit ddaC dendrite pruning. Moreover, the F-box protein Slimb forms a complex with Akt, an activator of the InR/PI3K/TOR pathway, and promotes Akt ubiquitination. We demonstrate that the Cul1-based SCF E3 ligase complex facilitates ddaC dendrite pruning primarily through inactivation of the InR/PI3K/TOR pathway. Finally, the SCF ligase and the InR/PI3K/TOR pathway regulate dendrite pruning in ddaC neurons at least in part by promoting local caspase activation in the dendrites. Thus, this study provides the first example, to our knowledge, of a critical E3 ligase complex in directing two distinct modes of pruning and also provides mechanistic insights into how this evolutionarily conserved SCF E3 ligase attenuates a key signaling pathway to promote pruning during the maturation of the nervous system.

## Results

### Cullin-1, a Core Component of the SCF E3 Ligase, Governs Pruning of ddaC Dendrites

We previously reported high efficacy and specificity of inducible RNAi knockdown in ddaC neurons during the larval-pupal transition [23]. It prompted us to carry out an unbiased genome-wide RNAi screen searching for novel players of dendrite pruning (Wong and Yu, unpublished data). In this large-scale screen, we isolated two independent RNAi transgenes, v108558 (#1) and v42445 (#2), corresponding to *cullin-1(cul1, also known as lin19)* [37]. RNAi knockdown of Cul1, via one copy of the *ppk-Gal4* driver that can target Gal4 expression in the class IV da neurons, led to dendrite pruning defects in ddaC neurons at 16 h after puparium formation (APF) (60%, *n* = 25 and 100%, *n* = 17, respectively; Figure S1A). The expression of *cul1* RNAi transgene (#2) with two copies of *ppk-Gal4* drivers caused severe defects with approximately 10.2 primary and secondary dendrites attached at 16 h APF (100%, *n* = 21; Figure 1C′ and 1G). Larval dendrites of *cul1* RNAi ddaC neurons were eventually removed by 48 h APF (*n* = 13, unpublished data), probably due to extensive migration and death of the abdominal epidermis on which the dendrites arborize [11]. Likewise, knockdown with other *cul1* RNAi lines, v33406 and v33407, also resulted in similar pruning defects at 16 h APF (unpublished data). In contrast, all dendrites were pruned completely in the wild-type ddaC neurons (*n* = 15; Figure 1B′ and 1G). The Cullin proteins serve as scaffold proteins of the SCF E3 ligase and interact with Roc1-Rbx1-Hrt1. We next generated transgenic flies expressing the dominant-negative form of Cul1 (Cul1^DN^) lacking its C-terminal putative Roc1-binding domain and neddylation site [38],[39]. The expression of Cul1^DN^ also resulted in consistent dendrite pruning defects at 16 h APF with an average of 4.5 primary and secondary dendrites (*n* = 20, 100%; Figure 1D′ and 1G). Development of major dendrite branches appears not to be affected in *cul1* RNAi and Cul1^DN^-expressing ddaC neurons, as judged by the number of their respective primary and secondary dendrites at the white prepupal (WP) stage (Figure 1C–D and 1G). Importantly, *cul1* RNAi (#2) ddaC neurons under one copy of *ppk-Gal4* exhibited normal larval dendrite morphology, as shown by the number of dendrite termini at the wandering 3^rd^ instar (wL3) stage (Figure S2A), while these mutant neurons showed notable dendrite pruning defects at 16 h APF (Figure S1A). Further, we made use of the RU486-inducible Gene-Switch system [40] to drive the expression of Cul1^DN^ from the early 3^rd^ instar (eL3) stage when larval dendrite morphogenesis was largely completed. RU486 treatment did not affect WP dendrite morphology in Cul1^DN^-expressing ddaC neurons (Figure S2B) or dendrite pruning in the control ddaC neurons (*n* = 35; Figure S2B). However, we consistently observed dendrite pruning defects in 67% of Cul1^DN^-expressing ddaC neurons at 16 h APF (*n* = 30; Figure S2B), contrasting with the noninduced controls (0%, *n* = 12, Figure S2B). The *Cul1^DN^* pruning defect in the Gene-Switch system was not as strong as those induced by *ppk-Gal4* driver (three copies) (Figure 1D′), due to weaker expression of the *elav-GS-Gal4* driver (one copy). Thus, *cul1* plays a specific role in dendrite pruning in ddaC neurons.

To further verify the role of *cul1* in ddaC dendrite pruning, we generated homozygous MARCM clones [41] using the previously reported null allele, *cul1^EX^* [37]. All *cul1^EX^* ddaC clones exhibited severe dendrite pruning defects with 15.8 of primary and secondary dendrites attached by 16 h APF (*n* = 6, 100%; Figure 1E′; WP, Figure 1E and 1G), which were fully rescued by the expression of the Cul1 protein (*n* = 8; Figure 1F′ and 1G). Likewise, *cul1^EX^* MARCM ddaD/E neurons failed to prune their larval dendrites at 18 h APF (*n* = 9, 100%; Figure S1B), compared to the wild-type neurons (*n* = 5; Figure S1B). *cul1^EX^* ddaF clones survived by 16 h APF (*n* = 4), whereas the wild-type ddaF neurons were apoptotic (*n* = 5; Figure S1C). Neddylation of Cul1, in which the ubiquitin-like polypeptide Nedd8 covalently conjugates onto the conserved lysine residue (Lys718), is essential for its activity and function [38]. Consistently, all ddaC clones from the null allele *nedd8^AN015^* exhibited dendrite pruning defects (*n* = 18; Figure S1D), further supporting the requirement of the Cul1-based E3 ligase activity for pruning of larval ddaC dendrites. Loss of *nedd8* function also disrupted ddaD/E pruning (*n* = 3; Figure S1B) and ddaF apoptosis (*n* = 4, Figure S1C). Thus, Cul1 is required for pruning and apoptosis of sensory neurons during early metamorphosis.

Taken together, Cul1 is identified as the core component of the SCF E3 ligase that regulates pruning and apoptosis of sensory neurons during early metamorphosis.

### The RING-Domain Protein Roc1a, But Not Roc1b, Is Required for ddaC Dendrite Pruning

**Figure 3:**
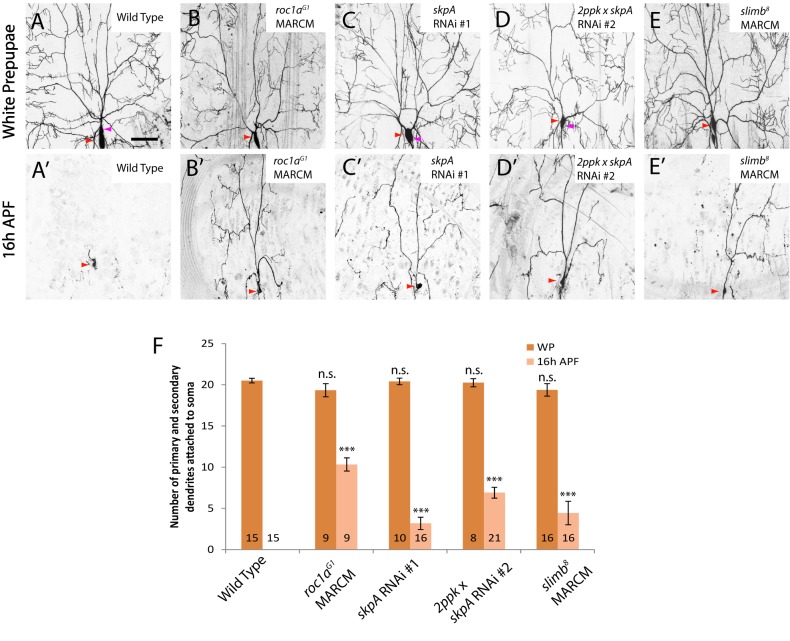
The RING-domain protein Roc1a, the adaptor protein SkpA, and the F-box protein Slimb are required for ddaC dendrite pruning. (A–E′) Live confocal images of ddaC neurons expressing *UAS-mCD8-GFP* driven by *ppk*-*Gal4* at WP and 16 h APF. Red arrowheads point to the ddaC somas, and purple arrowheads point to the ddaF soma also labelled by *ppk-Gal4* (Chr II). While wild-type neurons eliminated dendrites (A, A′), *roc1a^G1^* MARCM ddaC (B, B′), ddaC neurons overexpressing one copy of *skpA* RNAi #1 (C, C′) or two copies of *skpA* RNAi #2 (D, D′), or *slimb^8^* MARCM (E, E′) ddaC clones exhibited dendrite pruning defects at 16 h APF. (F) Quantification of the average number of primary and secondary dendrites attached to the soma of wild-type and mutant ddaC neurons at WP stage and 16 h APF. The number of samples (*n*) in each group is shown on the bars. Error bars represent S.E.M. ***p<0.001, n.s., not significant. Dorsal is up in all images. The scale bars are 50 µm. See genotypes in Text S1.

We next attempted to identify other components of the Cul1-based SCF E3 ligase complex that are required for ddaC dendrite pruning. The Cullin proteins, via their C-terminal regions, bind to the RING-domain protein Roc1-Rbx1-Hrt1, which recruits an ubiquitin conjugating enzyme E2 [31]. In *Drosophila*, two closely related Roc1 proteins, Roc1a and Roc1b, were reported to play differential roles during development [42],[43]. We observed a strong dendrite pruning defect in *roc1a* mutants, but not in *roc1b* mutants, suggesting a specific requirement of Roc1a for pruning. MARCM analysis of *roc1a^G1^*, a null allele [42], revealed a severe *cul1*-like pruning defect in all ddaC neurons: about 10.3 primary and secondary dendrites were attached at 16 h APF (*n* = 9; Figure 3B′ and 3F). Furthermore, RNAi knockdown of Roc1a, via independent v106315 (#1) and v32398 (#2) lines, also resulted in pruning defects (*n* = 22 and *n* = 24, respectively; Figure S3). Loss of *roc1a* function also led to severe defects in ddaD/E pruning (*n* = 2; Figure S4A) and ddaF apoptosis (*n* = 6; Figure S4B). In contrast, *roc1b* null mutants showed no defects in dendrite pruning of ddaC (*n* = 9; Figure S5) and ddaD/E neurons (*n* = 8; unpublished data) or ddaF apoptosis (*n = *8; unpublished data). Consistent with the preferential binding between Roc1b and Cul3 [43], MARCM ddaC clones for *cul3^gft2^*, a null *cul3* allele, did not exhibit apparent pruning defects (*n* = 14; Figure S5). These results underscore the specific roles of Cul1 and Roc1a in governing dendrite pruning.

Thus, Roc1a, like Cul1, plays an essential role in dendrite pruning of ddaC sensory neurons.

### The Adaptor Protein SkpA and the F-Box Protein Slimb Govern ddaC Dendrite Pruning

The N-terminal region of the Cullin proteins interacts with the adaptor subunit Skp1, and via it with an F-box protein to recruit protein substrates in proximity to the E2 enzyme, thereby promoting substrate ubiquitination [31]. We first examined the potential involvement of SkpA, a *Drosophila* homologue of Skp1 [44], in ddaC dendrite pruning. Knockdown of SkpA with three independent RNAi lines, BL28974 (#1), v32790 (#2), and v107815 (#3), resulted in consistent dendrite pruning defects in most of ddaC neurons (Figure 3C′ and 3F, and unpublished data). The expression of v32790 (#2) with two copies of *ppk-Gal4* driver resulted in moderate ddaC dendrite pruning defects with full penetrance: an average of 6.9 primary and secondary dendrites remained attached (*n* = 21, 100%; Figure 3D′ and 3F). Attenuation of *skpA* function also inhibited ddaD/E pruning (*n* = 13; Figure S4A) and ddaF apoptosis (*n* = 15; Figure S4B). No MARCM clones for *skpA^1^*, a *skpA* null allele, could be recovered in ddaC neurons, presumably due to its essential functions during cell division [44]. Hence, SkpA, like Cul1 and Roc1a, appears to govern ddaC dendrite pruning.

Given that the substrate specificity of the Cul1-based SCF E3 ligase complex is conferred by an F-box protein, we further performed a RNAi screen to examine the potential roles of 31 putative F-box proteins [45] in dendrite pruning. Among them, Supernumerary limbs (Slimb), which when knocked down via RNAi, exhibited dendrite pruning defects (Figure S3). Slimb, a *Drosophila* homologue of the mammalian β-TrCP proteins, acts to ubiquitinate Cubitus interruptus (Ci) and Armadillo, two key effectors of Hh and Wg signaling pathways, respectively [36],[45]. Knockdown of Slimb with two independent RNAi lines, v107825 (#1) and v34273 (#2), caused mild pruning defects (37.5%, *n* = 32 and 66.6%, *n* = 12, respectively; Figure S3). These phenotypes have been confirmed by generating MARCM ddaC clones of the null allele *slimb* ^8^, as stronger pruning defects were observed; 4.4 primary and secondary dendrites were attached by 16 h APF (*n* = 16, 50%, Figure 3E′ and 3F). Loss of *slimb* function also inhibited ddaD/E pruning (*n* = 8, 75%; Figure S4A) and ddaF apoptosis (*n* = 6, 50%; Figure S4B). Compared to those of *cul1*, *roc1a*, and *skpA*, the phenotypes of *slimb* mutants are less severe, presumably due to the perdurance of the wild-type protein. Alternatively, we cannot exclude the possible existence of other F-box proteins involved in ddaC dendrite pruning and ddaF apoptosis.

Thus, SkpA and Slimb appear to play important roles in regulating ddaC/D/E dendrite pruning and ddaF apoptosis.

### The Cul1-Based SCF E3 Ligase Complex Regulates Axon Pruning of MB γ Neurons

We next examined the potential requirements of Cul1, Roc1a, SkpA, and Slimb for MB axon pruning. In wild type, MB γ neurons selectively eliminated their larval axon branches by 24 h APF (*n* = 5; Figure 2A and 2B–B′) and regenerated the medial branches in the adulthood (*n* = 6; Figure 2G). Importantly, *cul1^EX^* mutant MB γ neurons retained many larval axons, which were labeled by *201Y-Gal4*-driven mCD8GFP expression (*n* = 19, 100%; Figure 2C′). These unpruned larval axons co-labeled by FasII were located outside the major FasII-positive α/β lobes at 24 h APF (arrowheads in the right panels of Figure 2C′) and persisted in the adult brains (*n* = 5, 100%; Figure 2H). In addition to the pruning defect, we also observed a neuroblast-proliferation defect, as adult *cul1* MB clones lacked late-born α/β neurons (Figure 2H). Moreover, the *cul1^EX^* axon pruning defects were fully rescued by reintroducing the Cul1 protein (Figure S6A). Thus, Cul1, a core component of the SCF E3 ligase, also plays a critical role in regulating axon pruning of MB γ neurons. The MB axon pruning and proliferation defects in *cul1^EX^* mutant resemble those in mutants of *uba1*, a single E1 gene in fly, as reported previously [13]. Interestingly, *roc1a^G1^* MARCM analyses also revealed a notable axon pruning defect in MB γ neurons. Unpruned axon branches, positively labeled by *201Y-Gal4*-driven mCD8GFP expression, remained at 24 h APF (*n* = 13, 62%; Figure 2D′). The axon pruning defects were rescued by expression of the full-length Roc1a protein (*n* = 11; Figure S6A). In contrast, *roc1b* exhibited normal pruning of MB γ axons at 24 h APF (Figure S6B). We were able to generate *skpA^1^* MARCM clones in MB γ neurons, all of which exhibited strong axon pruning defects at 24 h APF (*n* = 8, 100%; Figure 2E′). Likewise, MB axon pruning was also inhibited at 24 h APF in *slimb^2^* (78%, *n* = 14; Figure 2F′) and *slimb^8^* (57%, *n* = 14; unpublished data) MARCM clones. Finally, dendrite pruning of MB γ neurons failed to occur in *cul1^EX^* (*n* = 9, 100%) or *skpA^1^* (*n* = 4, 100%) MB neuroblast clones at 24 h APF (Figure S6C). Taken together, Cul1, Roc1a, SkpA, and Slimb govern both ddaC dendrite pruning and MB axon pruning, presumably as a Cul1-based SCF E3 ligase complex.

### The Cul1-Based SCF E3 Ligase Complex Acts Downstream of EcR-B1 and Sox14

**Figure 4:**
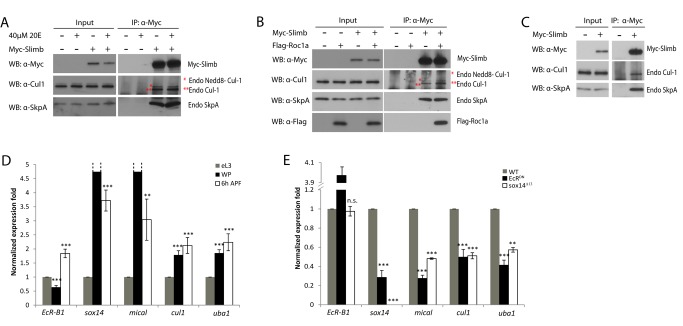
The Cul1-based SCF E3 ligase components interact with each other and *cul1* expression is dependent on EcR-B1 and Sox14. (A) Endogenous SkpA and Cul1 associated with Slimb in an ecdysone-independent manner. Nontreated (−) and ecdysone-treated S2 cells were transfected with Myc-Slimb. (B) Roc1a and Slimb associated in a complex with Cul1 and SkpA in S2 cells co-transfected with Myc-Slimb and Flag-Roc1a. (C) Endogenous SkpA and Cul1 co-immunoprecipitated with Myc-Slimb in the protein extracts from the wandering larval brains. (D) Expression profile of *EcR-B1*, *sox14*, *mical*, *cul1*, and *uba1* in the MB γ neurons at eL3, WP, and 6 h APF. mRNA level of *cul1*, like *sox14*, *mical*, and *uba1*, was upregulated upon white prepupal formation. (E) Expression profile of *EcR-B1*, *sox14*, *mical*, *cul1*, and *uba1* in MB γ neurons from wild-type or mutant animals at 6 h APF. mRNA level of *cul1* was significantly reduced in *EcR^DN^* expressing or *sox14^Δ13^* mutant MB γ neurons. **p<0.01, ***p<0.001, n.s., not significant. See genotypes in Text S1.

We next demonstrated the physical association among Cul1, Roc1a, SkpA, and Slimb. To this end, we first performed co-immunoprecipitation (co-IP) experiments in nontreated and ecdysone-treated S2 cells transfected with Myc-tagged Slimb. Endogenous unmodified Cul1 and Neddylated-Cul1 (Nedd8-Cul1 [37]), together with endogenous SkpA, were detected specifically in the immune complex when Myc-Slimb was immunoprecipitated from nontreated or ecdysone-treated protein extracts using an anti-Myc antibody (Figure 4A), suggesting that Cul1, SkpA, and Slimb form a protein complex, independently of ecdysone. Moreover, Roc1a was specifically co-immunoprecipitated using the protein extracts of S2 cells co-transfected with Flag-tagged Roc1a and Myc-Slimb (Figure 4B), also supported by a previous report that Roc1a preferentially associates with Cul1 in embryos [43]. We next performed coIP experiments using larval brain extracts expressing Myc-Slimb. Slimb was able to specifically co-immunoprecipitate with endogenous Cul1 and SkpA (Figure 4C), suggesting the *in vivo* association in postmitotic neurons.

**Figure 5:**
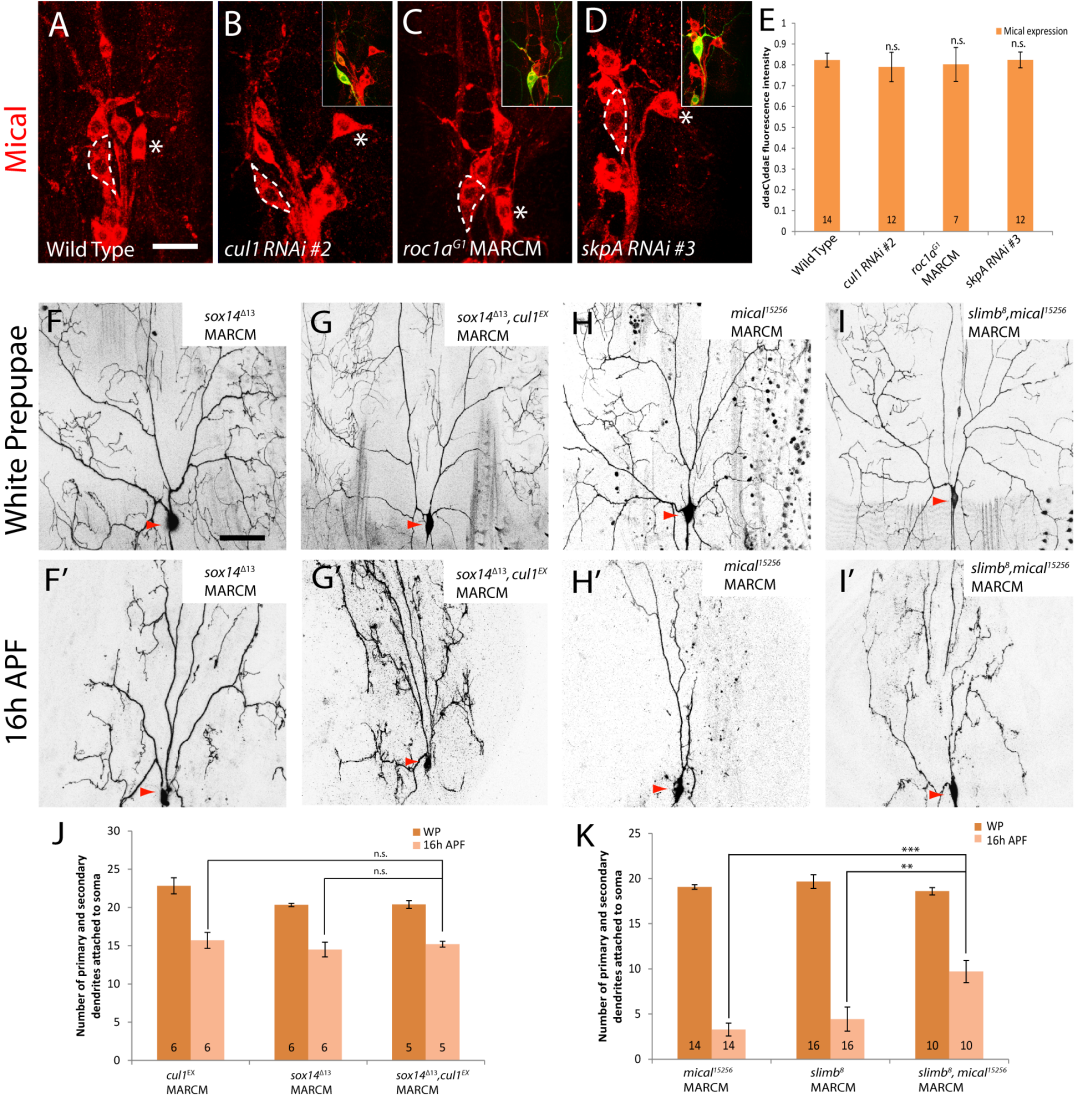
The Cul1-based SCF E3 ligase complex governs ddaC dendrite pruning in a Mical-independent pathway. (A–D) Confocal images show Mical stainings (in red) in various genotypes of ddaC neurons expressing *UAS-mCD8-GFP* driven by *ppk*-*Gal4* at WP. ddaC somas are marked by dashed lines, and ddaE by asterisks. The Mical expression in ddaC neurons of wild type (A), *cul1 RNAi* (B), *roc1a^G1^* MARCM (C), and *skpA* RNAi (D) remained largely unchanged. (E) Quantification of immunostaining for Mical was performed as described in Materials and Methods. The graph displays the average values of ddaC/ddaE ratios and S.E.M—n.s., not significant. *n* is shown on the bars. (E) The scale bar in (A) is 20 µm. (F–I′) Live confocal images of ddaC neurons expressing *UAS-mCD8-GFP* driven at WP and 16 h APF. (F and G) Although WP morphology remained largely unchanged, MARCM ddaC clones of *cul1^EX^* and *sox14^Δ13^* double mutant exhibited dendrite pruning defects with an average of 15.2 primary and secondary dendrites attached (G′), similar to either *cul1^EX^* (Figure 1E′) and *sox14^Δ13^* (F′) single mutant. (H and I) Although WP morphology remained unchanged, MARCM ddaC clones of *mical^15256^*, *slimb^8^* double mutants exhibited a significant enhancement of ddaC dendrite pruning defects (I′), compared to either *slimb^8^* (Figure 3E′) or *mical^15256^* (H′) single mutants. (J and K) Quantification of the average number of primary and secondary dendrites attached to the soma of mutant ddaC neurons at 16 h APF. The number of samples (*n*) in each group is shown on the bars. **p<0.01, ***p<0.001, n.s., not significant. Error bars represent S.E.M. Dorsal is up in all images. The scale bar in (F) is 50 µm. See genotypes in Text S1.

To investigate whether the Cul1 SCF E3 ligase expression is upregulated during the larval-pupal transition, we utilized Laser Capture Microdissection (LCM) technique to microdissect MB γ neurons from wild-type, *EcR^DN^*, and *sox14* brains, subject to total RNA extraction and quantitative real-time PCR experiments (Q-PCR). Importantly, mRNA levels of *cul1*, like *mical*, were significantly upregulated from eL3, WP to 6 h APF (Figure 4D). Upregulation of *cul1* transcription at 6 h APF, like *mical*, was strongly inhibited by EcR^DN^ expression or loss of *sox14* function (50.4% and 48.9% reduction, respectively; Figure 4E), suggesting that *cul1* upregulation is dependent on EcR-B1 and Sox14. Therefore, ecdysone signaling appears to regulate the abundance, but not assembly, of the Cul1 SCF E3 ligase complex. mRNA levels of the E1 gene *uba1* were also increased upon pupal formation (Figure 4D), in agreement with a previous microarray analysis showing that the UPS genes including *cul1* and *uba1* were upregulated in MB γ neurons during the larval-pupal transition [46]. Moreover, knockdown of *cul1* did not enhance the dendrite pruning defects in *sox14* null mutant ddaC neurons (*n* = 41; Figure S8). We also made MARCM ddaC clones for *sox14^Δ13^* and *cul1^EX^* double mutant and observed an average of 15.2 primary and secondary dendrites attached to the double mutant ddaC neurons (*n* = 5; Figure 5G′ and 5J), similar to either *cul1^EX^* (15.8; Figure 1E′ and 1G) or *sox14^Δ13^* (14.5, *n* = 6; Figure 5F′ and 5J) null MARCM neurons, supporting a linear relationship between *sox14* and *cul1*. Thus, *cul1* appears to act downstream of *sox14* during dendrite pruning.

Taken together, Cul1, Roc1a, SkpA, and Slimb function as the components of the SCF E3 ligase complex, among which the expression of *cul1* is dependent on *EcR-B1* and *sox14* during dendrite pruning.

### The Cul1-Based SCF E3 Ligase Complex Governs Dendrite Pruning in a Mical-Independent Manner

Given that the EcR-B1/Sox14/Mical pathway governs ddaC dendrite pruning [23], we next investigated how the SCF E3 ligase complex may integrate into this pathway. We first examined the protein levels of Mical, Sox14, and EcR-B1 in various SCF mutant ddaC neurons. Mical protein levels remained largely unchanged in the *cul1* RNAi ddaC neurons (*n* = 12; Figure 5B and 5E) or *cul1^EX^* MARCM (*n* = 8; unpublished data) at the WP stage, compared to those in the wild-type neurons (*n* = 14; Figure 5A and 5E). Likewise, the Mical levels were not affected in *roc1a^G1^* MARCM (*n* = 7; Figure 5C and 5E), *skpA* RNAi (*n = *12; Figure 5D and 5E), as well as *slimb^8^* MARCM (*n* = 13; unpublished data) ddaC neurons. Using a *mical-lacZ* reporter that drives upregulation of the LacZ expression under a *mical* enhancer (Y. Gu and F. Yu, unpublished data), we detected similar LacZ expression in wild-type, *cul1* RNAi, and *skpA* RNAi WP ddaC neurons (Figure S7A). These data indicate that the Cul1 SCF E3 ligase is dispensable for regulation of Mical transcription/expression in ddaC neurons. Moreover, the expression levels of EcR-B1 and Sox14 were unchanged in WP *cul1^EX^* mutant ddaC neurons, compared with the wild-type controls (Figure S7B). Likewise, the rest of the SCF components are not important for the expression of EcR-B1 and Sox14, as their protein levels were not affected in *roc1a^G1^*, *skpA* RNAi, *slimb^8^*, or *nedd8^AN015^* mutant ddaC neurons (Figure S7B). Thus, these data are consistent with the conclusion that the SCF complex functions downstream of *EcR-B1* or *sox14* during dendrite pruning.

Given that Mical expression is unaffected by the mutants of the SCF components, we hypothesized that the Cul1 SCF E3 complex might act in parallel with Mical during dendrite pruning. If this is true, we would expect enhancement of dendrite defects of *mical* null mutants by compromising the SCF components. Indeed, RNAi knockdown of Cul1 in *mical* mutant ddaC neurons resulted in a drastic pruning defect with the persistence of 10.1 primary and secondary dendrites at 16 h APF (*n* = 22; Figure S8), whereas the *mical* null ddaC neurons retained 5.3 major dendrites (*n* = 26; Figure S8). Likewise, knockdown of Roc1a (*n* = 36; Figure S8), SkpA, or Slimb (*n* = 22 and 30, respectively; unpublished data) significantly enhanced the *mical* null mutant phenotypes. Knockdown of *cul1* or *roc1a* in *mical* mutants exhibited normal elaboration of major dendrites at WP stage (Figure S9). Moreover, MARCM clones of *mical^15256^* and *slimb^8^* double null mutant also exhibited a significant enhancement of dendrite pruning defects (Figure 5K). An average of 9.7 primary and secondary dendrites were attached to the double mutant ddaC neurons (*n* = 10; Figure 5I′ and 5K), compared to either *mical^15256^* (3.3, *n* = 14; Figure 5H′ and 5K) or *slimb^8^* (4.4, *n* = 16; Figure 3E′ and 3F) null MARCM neurons. Thus, these data suggest that the Cul1 SCF E3 ligase facilitates ddaC dendrite pruning in parallel to Mical.

Taken together, our data suggest that the Cul1-based SCF E3 ligase complex acts downstream of EcR-B1/Sox14 and governs dendrite pruning in parallel with Mical.

### The Cul1-Based SCF Ligase Complex Attenuates the Insulin Pathway to Promote ddaC Dendrite Pruning

**Figure 6:**
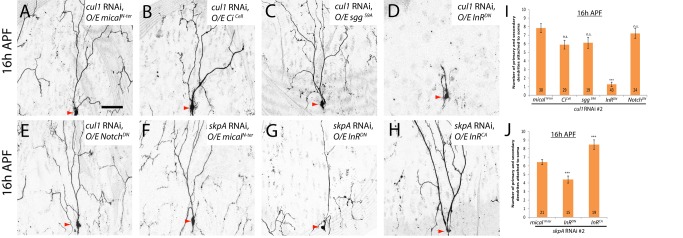
The Cul1-based SCF E3 ligase complex attenuates the Insulin signaling pathway to promote ddaC dendrite pruning. (A–H) Live confocal images of ddaC neurons expressing *UAS-mCD8-GFP* driven by *ppk*-*Gal4* at 16 h APF. Red arrowheads point to the ddaC somas. (A–E) Co-expression of *InR^DN^*, but not other dominant-negative repressors of various pathways, significantly suppressed the pruning defects in *cul1* RNAi ddaC neurons. Co-expression of nonfunctional *mical^N-ter^* (A), *Ci^Cell^* (B), *sgg^S9A^* (C), *InR^DN^* (D), and *Notch^DN^* (E) with *cul1* RNAi in ddaC neurons. Compared to the *skpA* RNAi, *mical^N-ter^* control (F), co-expression of *InR^DN^* significantly suppressed *skpA* RNAi-mediated pruning defects (G), whereas co-expression of *InR^CA^* significantly enhanced *SkpA* RNAi-mediated pruning defects in ddaC neurons (H). (I and J) Quantification of the average number of primary and secondary dendrites attached to the soma of mutant ddaC neurons at 16 h APF. The number of samples (*n*) in each group is shown on the bars. ***p<0.001, n.s., not significant. Error bars represent S.E.M. Dorsal is up in all images. The scale bar is 50 µm. See genotypes in Text S1.

During tissue growth and pattern formation, Cul1, Roc1a, and Slimb negatively regulate Hh or Wg pathways by specifically degrading their respective effectors, Ci or Armadillo [45]. We hypothesized that if the Cul1-based SCF complex also attenuates the Hh and/or Wg pathways during ddaC pruning, the dendrite pruning defects associated with loss of the SCF ligase complex would be attributable to hyperactivation of either or both pathways. However, three lines of evidence indicate that the Cul1-based SCF complex acts independently of Hh and Wg pathways during dendrite pruning. First, inhibition of either pathway did not suppress *cul1* RNAi dendrite pruning defects. Expression of the Hh repressors, Ci^Cell^ (*n* = 29; Figure 6B and 6I) or the Patched receptor (unpublished data), did not suppress the pruning defects associated with *cul1* RNAi, compared to the nonfunctional Mical^N-ter^ control (*n* = 30; Figure 6A and 6I) that was unable to rescue the dendrite pruning defect in *mical* mutant ddaC neurons and its expression alone did not interfere with ddaC dendrite pruning [23]. Likewise, expression of the Wg inhibitors, Sgg^S9A^ (*n* = 19; Figure 6C and 6I) or the truncated form of Dishevelled (unpublished data), did not suppress the *cul1* RNAi effects. Second, the expression of the nondegradable Hh activator Ci^U^ or the Wg activator Arm^S10^ did not affect normal ddaC dendrite pruning (Figure S10). Finally, forced expression of the Hh activators, Ci^U^ (*n* = 16; Figure S11) or the truncated form of Smoothened (Δ661–818; unpublished data), did not enhance the Cul1^DN^ pruning defects, compared to the Mical^N-ter^ control (*n* = 25; Figure S11). Similarly, the expression of the Wg activator Arm^S10^ did not affect the pruning defects in the Cul1^DN^-expressing ddaC neurons (*n* = 22; Figure S11).

To further investigate which signaling pathway might be attenuated by the Cul1-based SCF complex during ddaC pruning, we performed a candidate-based screen to systematically examine other important signaling pathways including Notch, Insulin, JNK, JAK/STAT, Hippo, EGFR, PVR, and Dpp. We expressed the dominant-negative repressors of these pathways, such as Notch^DN^, InR^DN^, Bsk^DN^, Dome^ΔCYT^, Yorkie^S168A^, EGFR^DN^, PVR^DN^, and Tkv^DN^, in *cul1* RNAi ddaC neurons. From this screen, the Insulin pathway was identified as a potential target pathway that is negatively regulated by the Cul1 SCF E3 ligase complex. Notably, the expression of a dominant negative form of the Insulin Receptor (InR^DN^), via one copy of *ppk-Gal4* driver, dramatically suppressed the dendrite pruning defects in the *cul1* RNAi-expressing ddaC neurons (*n* = 43; Figure 6D). On average, only 1.2 primary and secondary dendrites remained attached to these *InR^DN^*, *cul1* RNAi double ddaC neurons, contrasting with 7.8 major dendrites observed in the *mical^N-ter^*, *cul1* RNAi control (*n* = 30; Figure 6A and Figure 6I). In contrast, the expression of Notch^DN^ (*n* = 24; Figure 6E and 6I) or other repressors did not influence the *cul1* RNAi effects on ddaC dendrite pruning (Figure S12A). Expression of either of these dominant-negative transgenes did not affect normal ddaC pruning (Figure S10, unpublished data). The numbers of primary and secondary dendrites were primarily unchanged at the WP stage in various double-mutant combinations, except an apparent reduction in the *Sgg^S9A^*, *cul1* RNAi combination (Figure S12B).

To verify the specific effect of InR, we further conducted the genetic enhancement experiments in which InR^CA^ or Notch^CA^, known to activate Insulin or Notch pathways, respectively, was expressed in *cul1^DN^* ddaC neurons. The expression of InR^CA^ (*n* = 28; Figure S11) but not Notch^CA^ (*n* = 18; Figure S11) resulted in a significant enhancement of the *cul1^DN^*-associated pruning defects with full penetrance. Moreover, the InR^DN^ expression significantly mitigated either *cul1^DN^* (*n* = 22, Figure S13) or *skpA* RNAi (*n* = 15; Figure 6G and 6J) effects on dendrite pruning. Conversely, the expression of InR^CA^ significantly enhanced the ddaC dendrite pruning defects caused by *skpA* RNAi knockdown (*n* = 19; Figure 6H and 6J). The numbers of primary and secondary dendrites remained similar at the WP stage in these double-mutant combinations, compared with single mutants (Figure S12B).

Collectively, these genetic suppression/enhancement results indicate that the Cul1-based SCF complex promotes ddaC dendrite pruning primarily through attenuation of the Insulin pathway.

### The PI3K/TOR Pathway Is Inhibited by the Cul1-Based SCF Complex During ddaC Dendrite Pruning

**Figure 7:**
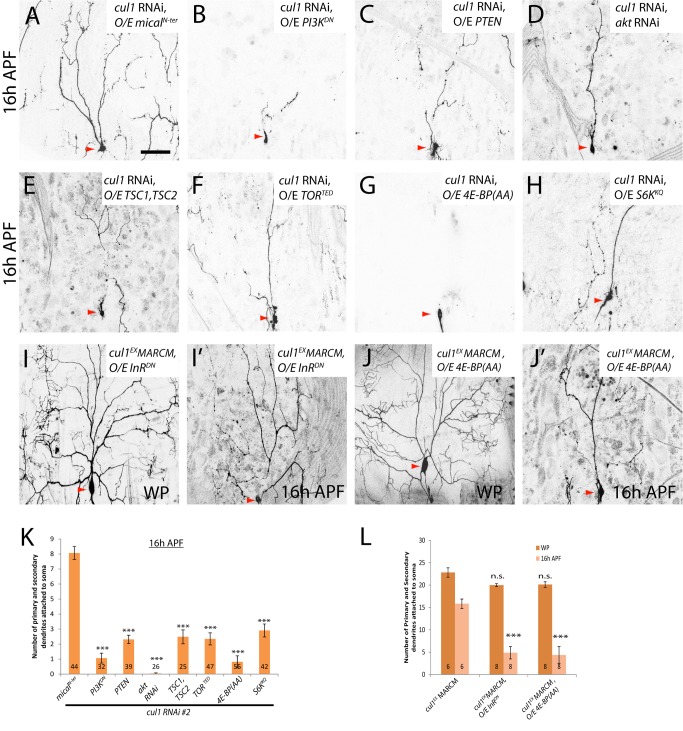
The PI3K/TOR pathway is inhibited by the Cul1-based SCF complex during ddaC dendrite pruning. (A–J′) Live confocal images of ddaC neurons expressing *UAS-mCD8-GFP* driven by *ppk*-*Gal4* at WP or 16 h APF. Red arrowheads point to the ddaC somas. While ddaC neurons co-expressing nonfunctional *mical^N-ter^* with *cul1* RNAi display strong pruning defects (A), co-expression of *PI3K^DN^* (B), *PTEN* (C), *akt* RNAi (D), *TSC1/TSC2* (E), *TOR^TED^* (F), *4E-BP(AA)* (G), or *S6K^KQ^* (H) strongly suppressed a *cul1* RNAi pruning defect at 16 h APF (K). Overexpression of *InR^DN^* (I and I′) or *4E-BP(AA)* (J and J′) in *cul1^EX^* ddaC MARCM clones did not affect the WP morphology but significantly suppressed the *cul1^EX^* pruning defect at 16 h APF. (K and L) Quantification of the average number of primary and secondary dendrites attached to the soma of mutant ddaC neurons at WP and 16 h APF. The number of samples (*n*) in each group is shown on the bars. Error bars represent S.E.M. Dorsal is up in all images. ***p<0.001. n.s., not significant. The scale bar is 50 µm. See genotypes in Text S1.

InR functions through the PI3K/TOR signaling pathway to mediate protein translation, metabolism, and ribosome biogenesis [47]. To examine whether the PI3K/TOR pathway could also be inhibited by the Cul1-based SCF complex during pruning, we performed genetic suppression assays by inactivating the PI3K/TOR pathway in *cul1* RNAi or *cul1^DN^* ddaC neurons. Importantly, the expression of the dominant-negative form of PI3K (PI3K^DN^) [48] or the Phosphatase and tensin homologue (PTEN) [49], both known to inactivate the PI3K pathway, like InR^DN^, drastically mitigated the dendrite pruning phenotypes in *cul1* RNAi ddaC neurons (*n* = 32 and 39, respectively; Figure 7B, 7C, and 7K). Compared to 8.1 primary and secondary dendrites in the Mical^N-ter^ control, PI3K^DN^ or PTEN expression resulted in an average of 1.1 or 2.3 primary/secondary dendrites connected to the *cul1* RNAi ddaC neurons (Figure 7K), respectively. Moreover, the expression of PI3K^DN^ (*n* = 22), PTEN (*n* = 21), or InR^DN^ (*n* = 22) largely rescued the *cul1^DN^*-mediated pruning defects (Figure S13). Thus, these data suggest that Cul1 also attenuates the PI3K pathway during ddaC pruning.

The PI3K pathway is interconnected with the Target of Rapamycin (TOR) pathway via TSC1 and TSC2, which act as negative regulators of the TOR pathway [47]. We next ascertained whether the TOR signaling pathway is also attenuated by the Cul1 SCF complex during ddaC dendrite pruning. Co-expression of TSC1 and TSC2, like PTEN, significantly suppressed the pruning defects in *cul1* RNAi (*n* = 25; Figure 7E and 7K) or *cul1^DN^* (*n* = 30; Figure S13) ddaC neurons. The truncated protein TOR^TED^ lacking its toxic effector domain behaves as the dominant-negative form to disrupt the TOR signaling [50]. The TOR^TED^ expression also strongly suppressed the dendrite pruning defects in *cul1* RNAi (*n* = 47; Figure 7F and 7K) or *cul1^DN^* (*n* = 24; Figure S13) ddaC neurons, respectively. The protein kinase TOR regulates protein synthesis via phosphorylation of the p70 ribosomal protein S6 kinase (S6K) [47] and the eukaryotic translation initiation factor 4E binding protein (4E-BP) [51]. S6K^KQ^, a catalytically inactive version, and 4E-BP(AA), a nonphosphorylated version, are able to repress protein translation and the TOR pathway [51],[52]. The expression of 4E-BP(AA) rescued the dendrite pruning defects in almost all *cul1* RNAi (*n = *55; Figure 7G and 7K) or Cul1^DN^-expressing (*n* = 32; Figure S13) ddaC neurons. Likewise, the expression of S6K^KQ^ also dramatically mitigated the dendrite pruning defects caused by *cul1* RNAi knockdown (*n* = 42, Figure 7H and 7K) or *cul1^DN^* expression (*n* = 18; Figure S13). Moreover, the expression of InR^DN^ (*n* = 8; Figure 7I′) or 4E-BP(AA) (*n* = 8; Figure 7J′) also strongly suppressed the dendrite pruning defects in *cul1^EX^* MARCM ddaC neurons, as approximately 4.9 and 4.3 primary and secondary dendrites remained attached at 16 h APF, respectively, compared to 15.8 major dendrites remaining in *cul1^EX^* mutant neurons (Figure 7L). To further confirm these genetic suppressions, we attenuated the InR/PI3K/TOR pathway by feeding the 3^rd^ instar larvae with Rapamycin, a pharmacological inhibitor of TOR. Rapamycin treatment did not affect the onset of puparium formation/adult eclosion, larval dendrite development (Figure S14A) or wild-type ddaC dendrite pruning (Figure S14B). However, Rapamycin treatment significantly suppressed the ddaC dendrite pruning defects in *cul1* RNAi ddaC neurons (*n* = 58), but not in *mical* RNAi neurons (*n* = 48, Figure S14B). Thus, these data further support the conclusion that the InR/PI3K/TOR pathway is inactivated by the Cul1-based SCF complex during ddaC dendrite pruning.

The following lines of evidence indicate the specificity of these suppression effects. First, attenuation of the InR/PI3K/TOR pathway did not affect normal elaboration of major dendrites in *cul1* RNAi or *cul1^DN^* mutant ddaC neurons, as the numbers of their primary and secondary WP dendrites were essentially unchanged despite the simplified terminal branches (Figure S15A and S15B). Inactivation of the InR/PI3K/TOR pathway alone did not affect normal ddaC dendrite pruning (Figure S10 and unpublished data). More importantly, the severing of major dendrites, a hallmark feature of dendrite pruning, occurred, similar to the wild type, in these suppression experiments (empty arrowheads, Figure S16A), suggesting that inactivation of the InR/PI3K/TOR pathway restores the severing of proximal dendrites from the *cul1* RNAi ddaC neurons. Furthermore, the expression of InR^DN^, PI3K^DN^, PTEN, TOR^TED^, S6K^KQ^, or 4E-BP(AA) was not able to suppress the dendrite pruning defects associated with *mical* RNAi (unpublished data) or *mical^15256^* mutant ddaC neurons (Figure S16B), supporting their specific genetic interactions with the SCF components.

Taken together, the InR/PI3K/TOR pathway is specifically inhibited by the Cul1-based SCF E3 complex in order to promote ddaC dendrite pruning during early metamorphosis.

### Slimb Forms a Protein Complex with Akt and Promotes Akt Ubiquitination

**Figure 8:**
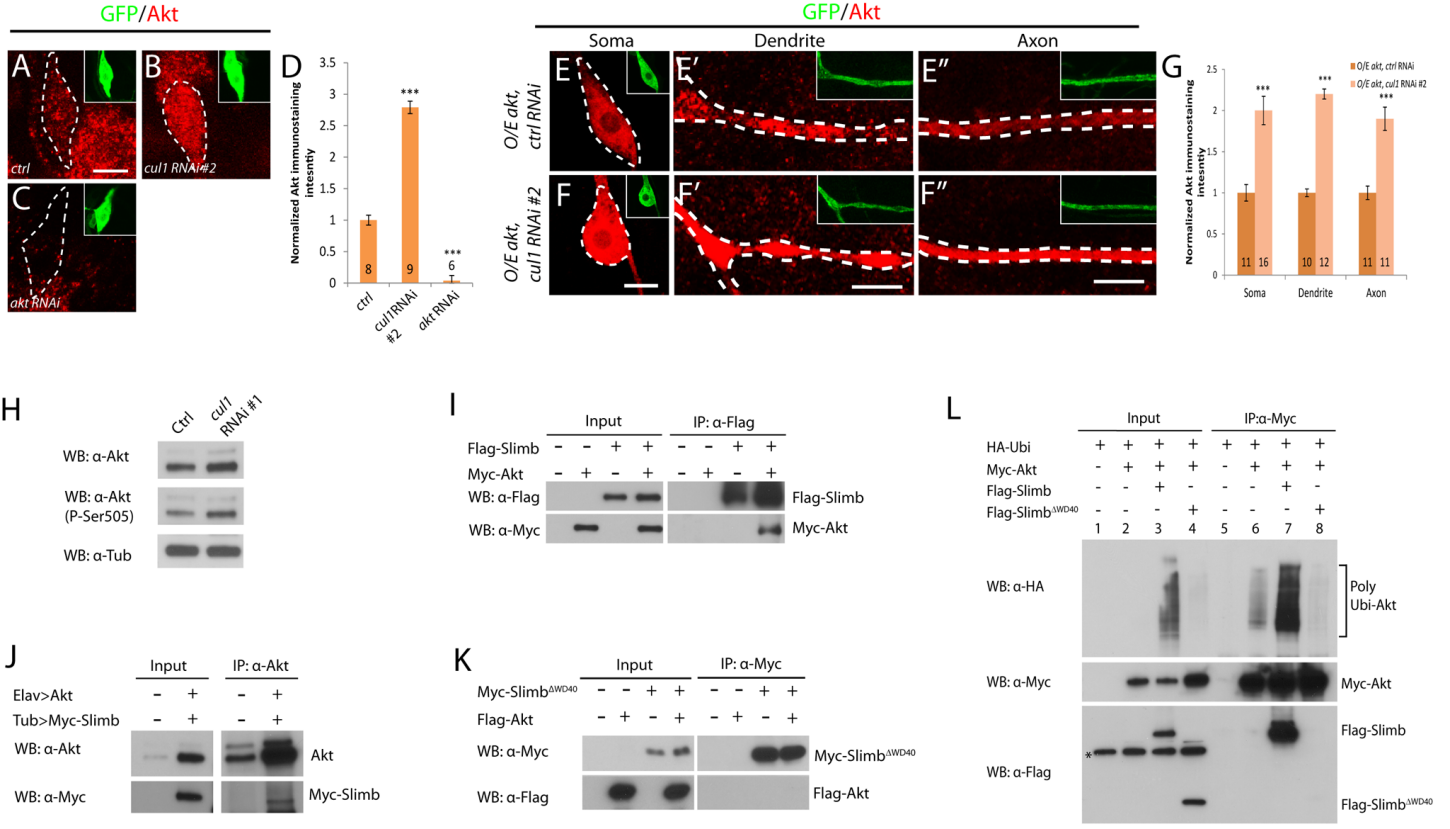
Slimb forms a protein complex with Akt and promotes Akt ubiquitination. (A–C and E–F″) Confocal images show Akt stainings (in red) in various genotypes of ddaC neurons expressing *UAS-mCD8-GFP* driven by *ppk*-*Gal4* at WP. ddaC somas/dendrites/axons are marked by dashed lines. Endogenous Akt level was significantly upregulated in the somas of *cul1* RNAi ddaC neurons (B and D) compared to that in the control RNAi somas (A and D). Akt signals were abolished in *akt* RNAi ddaC neurons (C and D). Overexpressed Akt is upregulated in *cul1* RNAi ddaC somas, dendrites, and axons (F–F″ and G), compared to the control RNAi (E–E″ and G). Quantification of Akt immunostaining was performed as described in Materials and Methods (D and G). The graph displays the normalized Akt immunostaining intensity and S.E.M.; *n* is shown on the bars ***p<0.001. The scale bars are 5 µm. (H) Akt expression and activity were upregulated in the *cul1* RNAi brain extracts. (I) Slimb and Akt associated each other in S2 cells cotransfected with Flag-Slimb and Myc-Akt. (J) Akt associated with Myc-Slimb in brain extracts expressing Myc-Slimb and Akt. (K) The mutant F-box protein, Slimb^ΔWD40^, lacking its substrate-recognition WD40 domains, did not associate with Akt in S2 cells co-transfected with Myc-Slimb^ΔWD40^ and Flag-Akt. (L) In vivo ubiquitination assay, Slimb but not Slimb^ΔWD40^ enhanced ubiquitination of Akt in S2 cells overexpressing HA-Ubiquitin, Myc-Akt, and Flag-Slimb or Flag-Slimb^ΔWD40^. * indicates a nonspecific band. See genotypes in Text S1.

Since inactivation of the InR/PI3K/TOR pathway suppresses the dendrite pruning defects in ddaC neurons lacking the Cul1 E3 ligase activity, we next assessed whether compromised E3 ligase function causes hyperactivation of the InR/PI3K/TOR pathway. To this end, we examined the expression and activity of Akt, a positive regulator of the InR/PI3K/TOR pathway, in ddaC neurons and 6 h APF brain lysates. Using an anti-Akt antibody, a weak localization of endogenous Akt was detected in ddaC somas (Figure 8A), but not in dendrites and axons (unpublished data). Endogenous Akt was significantly upregulated in the somas of *cul1* RNAi ddaC neurons at the WP stage (2.8 folds, *n* = 9; Figure 8B and 8D), compared to the wild-type somas (*n* = 8, Figure 8A and 8D). Akt signals were abolished in *akt* RNAi ddaC neurons (Figure 8C and 8D). Since overexpressed Akt could be observed weakly in axons and dendrites in addition to its robust localization in ddaC somas (Figure S17), we co-expressed Akt with *cul1* RNAi or the control RNAi to examine the Akt protein levels throughout the neurons. Consistently, *cul1* RNAi knockdown also caused a significant increase in Akt protein levels in ddaC somas (2 folds, *n* = 16), dendrites (2.2 folds, *n* = 12), and axons (1.9 folds, *n* = 11; Figure 8F–F″ and 8G), compared to the RNAi controls (Figure 8E–E″ and 8G). As a control, the expression levels of GFP were the same or similar throughout *cul1* RNAi or the control RNAi ddaC neurons (unpublished data). These data suggest that SCF-dependent Akt degradation is not restricted to dendrites of ddaC neurons. Consistently, the Cul1 SCF E3 complex appears to be localized throughout ddaC neurons as indicated by uniform distribution of exogenously expressed SkpA-RFP in the dendrites, axons, and somas (*n* = 7; Figure S17). Moreover, the protein levels of Akt were significantly increased in the *cul1* RNAi brain lysates (the upper panel, Figure 8H) where Cul1 proteins were knocked down via the *cul1* RNAi line (#1) using a pan-neuronal driver *elav-Gal4* (Figure S18A). Concomitantly, Akt activity was also substantially increased, as judged by an increase in active and phosphorylated Akt levels (the middle panel, Figure 8H). Thus, the Cul1-based SCF E3 ligase negatively regulates Akt expression and activity in ddaC neurons and prepupal brains. We then investigated whether attenuation of Akt suppresses the dendrite pruning defects in *cul1* RNAi ddaC neurons. Interestingly, knockdown of Akt with two independent RNAi lines (#1: BL31701 or #2: BL33615) potently suppressed the dendrite pruning defects in *cul1* RNAi ddaC neurons (*n* = 26, Figure 7D; unpublished data). Reduction of *akt* function resulted in an average of 0.2 primary/secondary dendrites connected to the *cul1* RNAi ddaC neurons (Figure 7K), compared to 8.1 in the Mical^N-ter^ control (Figure 7A and 7K). These biochemical and genetic data indicate that compromised SCF E3 ligase function causes hyperactivation of the InR/PI3K/TOR pathway.

To further examine a potential link between the Cul1 SCF E3 ligase and the InR/PI3K/TOR pathway, we assessed the physical interaction between Akt and the F-box protein Slimb. The C-terminal region of Slimb contains seven WD40 domains that are responsible for binding to its substrates and targeting them for ubiquitination. Interestingly, Akt was co-immunoprecipitated with Slimb using the protein extracts of S2 cells co-transfected with Myc-Akt and Flag-Slimb (Figure 8I). We confirmed this interaction in postmitotic neurons, as Slimb was specifically co-immunoprecipitated with Akt in the prepual brain extracts expressing Myc-Slimb and Akt (Figure 8J). Furthermore, Akt specifically interacted with Slimb, as Akt was not pulled down by either the truncated Slimb protein lacking its WD40 domains (Slimb^ΔWD40^, Figure 8K) or another F-box protein Ago (Figure S18B). We then investigated whether Slimb can mediate ubiquitination of Akt. Notably, Slimb expression strongly increased the amount of polyubiquitinated Akt (lane 7, Figure 8L), compared to the control (lane 6, Figure 8L). In contrast, the expression of Slimb^ΔWD40^ failed to facilitate Akt ubiquitination (lane 8, Figure 8L), suggesting that the WD40 domains are responsible for Slimb-mediated ubiquitination of Akt. Thus, Slimb associates with Akt and targets Akt for ubiquitination.

### Activation of the InR/PI3K/TOR Pathway Alone Is Sufficient to Inhibit ddaC Dendrite Pruning

**Figure 9:**
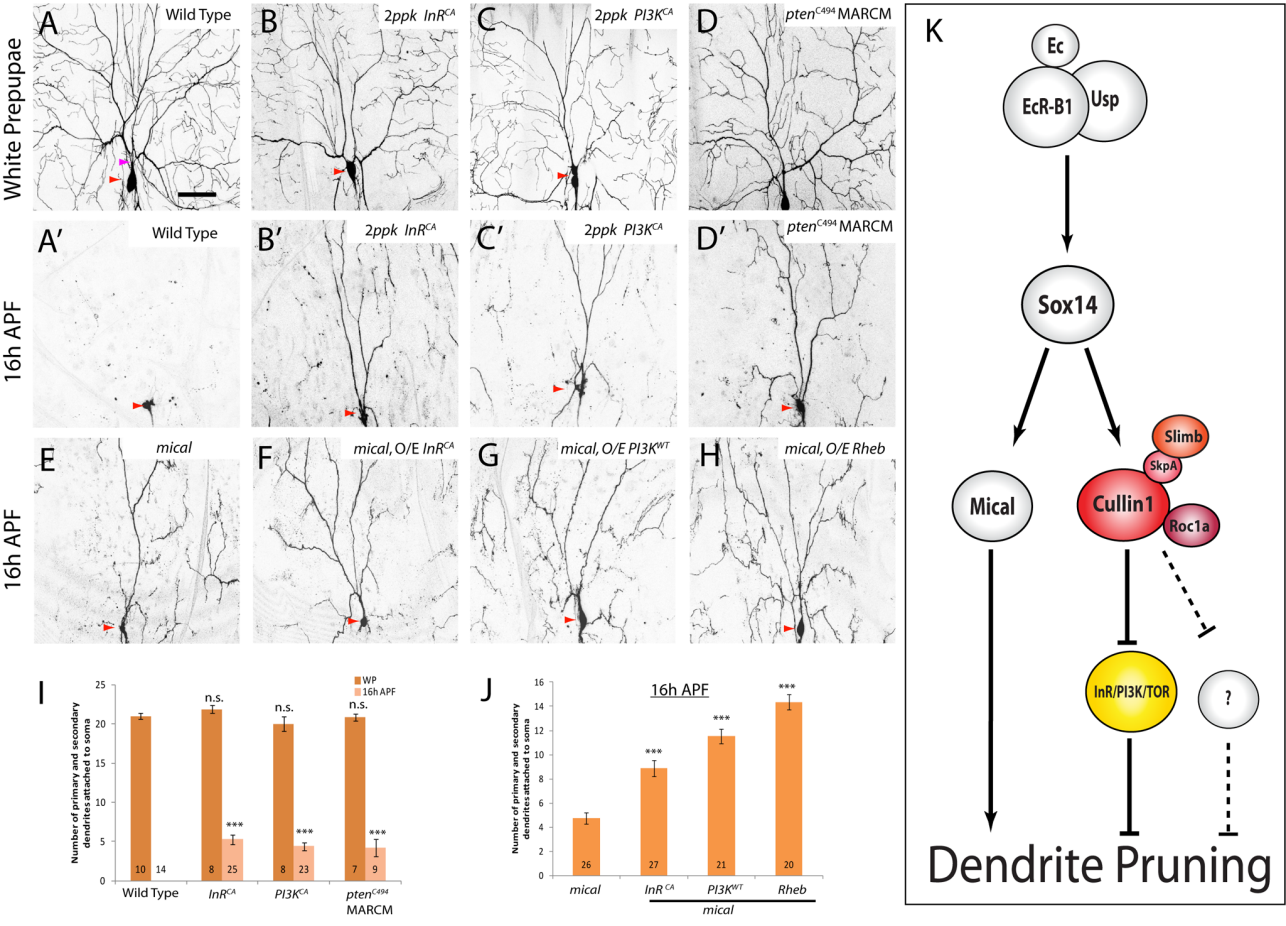
Activation of the InR/PI3K/TOR pathway alone is sufficient to inhibit ddaC dendrite pruning. (A–H) Live confocal images of ddaC neurons expressing *UAS-mCD8-GFP* driven by *ppk*-*Gal4* at WP or 16 h APF. Red arrowheads point to the ddaC somas. Activation of the InR/PI3K/TOR pathway through overexpression of *InR^CA^* (B, B′), *PI3K^CA^* (C, C′), or *pten^c494^* MARCM (D, D′) led to prominent pruning defects at 16 h APF. While *mical* ddaC neurons displayed an obvious pruning defect at 16 h APF (E), activation of the InR/PI3K/TOR pathway by *InR^CA^* (F), *PI3K^WT^* (G), or *Rheb* (H) significantly enhanced the pruning defects in the *mical* ddaC neurons. (I and J) Quantification of the average number of primary and secondary dendrites attached to the soma of mutant ddaC neurons at WP and 16 h APF. The number of samples (*n*) in each group is shown on the bars. Error bars represent S.E.M. Dorsal is up in all images. ***p<0.001, n.s., not significant. The scale bar is 50 µm. See genotypes in Text S1. (K) A model for the Cul1-based SCF E3 ligase and the InR/PI3K/TOR pathway during ddaC dendrite pruning. The Cul1-based SCF E3 ligase acts downstream of EcR-B1 and Sox14 but in parallel to the Mical pathway during ddaC dendrite pruning. The Cul1-based SCF E3 ligase complex facilitates ddaC dendrite pruning primarily through inactivation of the InR/PI3K/TOR pathway. The InR/PI3K/TOR pathway negatively regulates ddaC dendrite pruning.

We have demonstrated that inactivation of the InR/PI3K/TOR pathway by the Cul1-based SCF E3 ligase complex is required to facilitate ddaC dendrite pruning during early metamorphosis. To further substantiate it, we examined whether constitutive activation of the InR/PI3K/TOR pathway alone is sufficient to inhibit normal progression of ddaC dendrite pruning. Notably, the expression of InR^CA^, or PI3K^CA^, both known to constitutively activate the InR/PI3K pathway [48],[53],[54], via two copies of *ppk-Gal4* driver, caused consistent dendrite pruning defects in the vast majority of ddaC neurons (InR^CA^, *n = *25, 92%; and PI3K^CA^, *n* = 23, 91%; Figure 9B′ and 9C′). On average, 5.2 (InR^CA^) and 4.3 (PI3K^CA^) primary/secondary dendrites retained the attachment to their respective ddaC neurons at 16 h APF (Figure 9I). The dendrite pruning defect caused by InR^CA^ expression was fully suppressed by *akt* RNAi knockdown (*n* = 20; Figure S19D), suggesting that expression of InR^CA^ likely activates residual Akt to inhibit dendrite pruning. Supportively, using two *PTEN* null/strong alleles, *PTEN^C494^ and PTEN^1^*, we observed similar dendrite pruning defects in ddaC neurons (89%, *n* = 9 and 48%, *n* = 21, respectively; Figures 9D′ and S19B). Loss of *PTEN* function also inhibited ddaD/E dendrite pruning, but not ddaF apoptosis or MB axon pruning (Figure S19B and S19C, unpublished data). Moreover, the expression of the small GTPase Rheb (Ras homologue enriched in brain) or the constitutively active form of S6K (S6K^STDETE^), activators of the TOR pathway [47],[52], led to dendrite pruning defects in the majority of ddaC neurons (77%, *n* = 25 and 63%, *n* = 24, respectively; Figure S19A). Activation of the InR/PI3K/TOR pathway appears not to affect the morphology and numbers of their major WP dendrites (Figures 9A–D and S19A). Further, we co-expressed InR^CA^, PI3K^CA^, Rheb, or S6K^STDETE^ with Cul1^DN^ (Figure S20) or *roc1a* RNAi (unpublished data) in ddaC neurons, resulting in more severe dendrite pruning defects.

Similar to the Cul1 SCF E3 ligase, the InR/PI3K/TOR pathway regulates ddaC dendrite pruning in a Mical-independent manner. First, the protein levels of Mical, Sox14, or EcR-B1 were unaffected upon activation of the InR/PI3K/TOR pathway, via InR^CA^/PI3K^CA^ expression or loss of *PTEN* function (Figure S21A–B). The upregulation of the Mical expression during the larval-pupal transition was also unaffected upon inactivation of the InR/PI3K/TOR pathway (unpublished data). Second, inactivation of the InR/PI3K/TOR pathway, via expression of InR^DN^, PI3K^DN^, PTEN, TOR^TED^, S6K^KQ^, or 4E-BP(AA), did not suppress the dendrite pruning defects in *mical* null mutant ddaCs (Figure S16B). Finally, activation of the InR/PI3K/TOR pathway, via expression of InR^CA^ (*n* = 27, Figure 9F), PI3K (*n* = 21, Figure 9G), or Rheb (*n* = 20, Figure 9H), significantly enhanced the *mical* null mutant pruning phenotypes (Figure 9J).

Local caspase activation in dendrites was shown to be required for elimination of dendrites in ddaC neurons [24],[25]. We therefore assessed whether SCF and InR/PI3K/TOR govern dendrite pruning through local caspase activation. Using the genetically encoded caspase reporter CD8::PARP::Venus [25], we observed no or negligible caspase activity in dendrites of *cul1* RNAi (*n* = 9) or InR^CA^-expressing (*n* = 6) ddaC neurons at 6 h APF, in contrast to strong caspase activity in wild-type ddaC dendrites (*n* = 6; Figure S22). Therefore, the SCF ligase and the InR/PI3K/TOR pathway regulate dendrite-specific pruning in ddaC neurons at least in part by promoting local caspase activation in the dendrites.

In summary, our data indicate that activation of the InR/PI3K/TOR pathway alone is sufficient to inhibit ddaC dendrite pruning in a Mical-independent manner. Thus, we demonstrate that during early metamorphosis, the Cul1-based SCF E3 ligase complex facilitates ddaC dendrite pruning primarily through inactivation of the InR/PI3K/TOR pathway.

## Discussion

Previous studies showed that the UPS activity plays an intrinsic and essential role in governing both modes of pruning in ddaC [12] and MB γ neurons [13]. However, little is known about the E3 ubiquitin ligase that is able to direct two distinct modes of neuronal pruning in *Drosophila*. Moreover, it is also unknown which downstream pathway is inactivated by the E3 ligase in order for ddaC neurons to prune their dendrites. Here, we report the identification of the Cul1-based SCF E3 ligase complex that plays a critical role in both modes of pruning of ddaC and MB γ neurons. In a genome-wide RNAi screen, we first isolated Cul1, a core scaffold protein of the SCF E3 ligase, which is required for ddaC dendrite pruning and MB axon pruning during early metamorphosis. We further identified the other components of the E3 ligase complex including the RING domain protein Roc1a, the adaptor protein SkpA, as well as the F-box protein Slimb. These molecules, like Cul1, are all required for pruning of ddaC and MB γ neurons. We show that the Cul1-based SCF E3 ligase acts downstream of EcR-B1/Sox14 and promotes ddaC dendrite pruning in a Mical-independent manner. Moreover, via a candidate-based screen, we observed that during ddaC dendrite pruning, the Cul1 E3 ligase negatively regulates the InR/PI3K/TOR signaling pathway but not other major developmental pathways examined. We demonstrate that inactivation of the InR/PI3K/TOR pathway leads to strong suppression of dendrite pruning defects in ddaC neurons deficient in the Cul1 E3 ligase activity, whereas activation of the InR/PI3K/TOR pathway alone is sufficient to inhibit ddaC dendrite pruning. Thus, the Cul1-based SCF E3 ligase promotes dendrite pruning in ddaC neurons primarily through inactivation of the InR/PI3K/TOR pathway (Figure 9K).

### The Cul1-Based SCF E3 Ubiquitin Ligase Regulates Pruning of ddaC Dendrites and MB Axons in *Drosophila*

Previous studies indicated that UPS activity is cell-autonomously required for both MB axon pruning and ddaC dendrite pruning in *Drosophila* [12],[13]. First, the expression of a yeast ubiquitin protease, which eliminates ubiquitin from substrates and inhibits UPS-mediated degradation [55], causes severe pruning defects in MB γ and ddaC neurons [12],[13]. Second, loss of the E1 enzyme Uba1 leads to strong pruning defects in both types of neurons. Third, removal of the proteasome subunits, Mov34 or Rpn6, also results in *uba1*-like pruning defects in MB and ddaC neurons. The selectivity of the UPS machinery for pruning is mainly conferred by a specific E3 ligase. Given the existence of a large number of E3 ligases in the *Drosophila* genome, it is challenging to identify the specific one that directs these two modes of neuronal pruning. It was reported that DIAP1, an RING E3 ligase, antagonizes the Dronc caspase activity and inhibits ddaC dendrite severing [24]. A model had been proposed that autoubiquitination and degradation of DIAP1 allows for the activation of the Dronc caspase in ddaC dendrites and thereby pruning of ddaC neurons. However, recent studies reported that all ddaC neurons sever their dendrites normally in DIAP1 gain-of-function mutants [26] and overexpression condition [25]. Nevertheless, Dronc caspase and DIAP1 appear not to be important for axon pruning of MB γ neurons, as analyses of *Dronc* MARCM clones [24], expression of the caspase inhibitor p35 [13], or gain of *DIAP1* function (Wong and Yu, unpublished data) revealed no axon pruning defects. Thus, the question of which specific E3 ligase directs both modes of pruning remains open for a long time. In this study, we demonstrate that the Cul1-based SCF E3 ligase complex plays key roles in regulating both ddaC dendrite pruning and MB axon pruning during *Drosophila* metamorphosis.

Several lines of evidence indicate that the Cul1-based SCF E3 ligase complex is the specific one that governs both ddaC dendrite pruning and MB axon pruning. First, while Cul1 and its binding partner Roc1a are critical for pruning of ddaC and MB neurons, Cul3 and Roc1b that preferentially bind each other are not important. Second, the Cul4-based E3 ligase, which regulates TSC2 protein stability and TSC1/2 complex turnover in *Drosophila* [56], is dispensable for ddaC dendrite pruning. MARCM analyses of the null allele *cul-4^11L^* exhibited no ddaC dendrite pruning defect (*n* = 5; Figure S5). Consistently, overexpression of TSC1/TSC2 complex did not inhibit ddaC pruning (unpublished data). Third, among 31 fly F-box proteins, Slimb was identified, which when knocked down via RNAi, resulted in apparent dendrite pruning defects in ddaC neurons. Furthermore, Slimb appears to be important for both ddaC dendrite pruning and MB γ axon pruning. Another F-box protein, Archipelago (Ago), is not required for ddaC pruning, in contrast to the essential role of its worm homologue SEL10 in synapse elimination [35]. MARCM analyses with *ago^3^*, a null allele [57], revealed no pruning defect in ddaC neurons (*n* = 3; Figure S5), supporting the selectivity of the E3 ligases for pruning. Finally, Nutcracker, the F-box protein of an SCF ubiquitin ligase (E3) required for caspase activation during sperm differentiation [58], appears not to be essential for ddaC pruning (*n* = 9, unpublished data).

### The Cul1-Based SCF E3 Ligase Acts in Parallel to Mical to Promote ddaC Dendrite Pruning

We previously reported that a transcriptional hierarchy, consisting of EcR-B1, Sox14, and Mical, is commonly induced in both ddaC neurons and MB γ neurons, and essential for ddaC dendrite pruning [23]. How is the Cul1 E3 ligase integrated into this linear pathway? We propose that the Cul1-based SCF E3 ligase likely acts downstream of the transcriptional activators EcR-B1 and Sox14, however, in parallel to Mical, during ddaC dendrite pruning (see the model in Figure 9K). First, previous microarray analyses showed that the UPS genes, involved in all steps of the UPS pathway including *cul1* and *skpA*, appear to be upregulated in remodeling MB γ neurons during the larval-pupal transition [46]. Upregulation of these genes is abolished by the expression of EcR^DN^ [46]. Second, our Q-PCR data further verify that *cul1* is a downstream effector of *EcR-B1* and *sox14*, suggesting ecdysone signaling regulates the abundance, but not assembly, of the Cul1 SCF E3 ligase complex. Third, ddaC neurons devoid of the Cul1 E3 ligase retain strong expression of Sox14. Reduction of *cul1* function does not enhance *sox14* mutant phenotype, supporting that the Cul1 E3 ligase acts downstream of *sox14* during ddaC pruning. Finally, the Cul1-based E3 ligase is dispensable for the transcription and translation of *mical* in ddaC neurons, suggesting that it likely exerts its roles in a Mical-independent pathway. This is further supported by the fact that reduction of the E3 ligase function in *mical* null mutant ddaC neurons results in additive effects on dendrite pruning. Taken together, our data support the model in which the Cul1-based SCF E3 ligase acts in parallel with Mical to govern ddaC dendrite pruning, downstream of EcR-B1 and Sox14 (Figure 9K).

### The Cul1-Based E3 Ligase Inactivates the InR/PI3K/TOR Pathway During ddaC Dendrite Pruning

During pattern formation, the Cul1-based SCF E3 ligase degrades Ci or Arm to negatively regulate Hh or Wg pathways, respectively [45]. Importantly, our data demonstrate that the InR/PI3K/TOR pathway, rather than Wg or Hh pathways, is inactivated by the Cul1-based E3 ligase in order for ddaC neurons to prune their dendrites. First, we show that inactivation of the InR/PI3K/TOR pathway suppresses the dendrite pruning defects in ddaC neurons lacking the Cul1 E3 ligase activity. Second, compromised *cul1* function causes enhanced expression/activity of Akt, a positive regulator of the InR/PI3K/TOR pathway. These data suggest that compromised E3 ligase function causes activation of the InR/PI3K/TOR pathway. Consistently, a reversal of the UPS pathway by a deubiquitinating enzyme also leads to enhancement of the insulin signaling in mammals [59]. Finally, activation of the InR/PI3K/TOR pathway alone is sufficient to inhibit ddaC dendrite pruning. In contrast, activation of the InR/PI3K/TOR pathway by loss of *PTEN* function (*n* = 11) or InR^CA^ expression (*n* = 7) is dispensable for MB axon pruning (Figure S19C). Given the distinct architecture and morphology of ddaC dendrites and MB γ axons, it is conceivable that the SCF E3 ligase regulates differential target pathways during these two types of pruning. Like the Cul1 E3 ligase, the InR/PI3K/TOR pathway acts downstream of EcR-B1/Sox14 and regulates dendrite pruning in parallel to Mical (see the model in Figure 9K).

It has been reported that ecdysone can inhibit the release of insulin-like peptides from insulin-producing-cells during the larval-pupal transition, systemically inactivate the InR/PI3K/TOR pathway, and thereby terminate larval growth [60]. We show that during dendrite pruning, ecdysone signaling increases abundance of the Cul1-based SCF complex, which in turn inactivates the InR/PI3K/TOR pathway (Figure 9K). These regulations with different mechanisms together would ultimately ensure lower levels of InR/PI3K/TOR activity/function in ddaC neurons, thereby resulting in dendrite pruning in these neurons during early metamorphosis. How does the Cul1-based SCF E3 ligase inactivate the InR/PI3K/TOR pathway during ddaC pruning? This negative regulation may occur through direct degradation of positive regulators of the pathway, such as IRS (fly Chico), Akt, and TOR. Several lines of evidence indicate that Akt is a good candidate as a substrate of the Cul1 SCF E3 ligase. First, the F-box protein Slimb, a component of the Cul1 SCF E3 ligase required for substrate recognition, specifically interacts with Akt. Second, Slimb promotes ubiquitination of Akt in a WD40-dependent manner. Third, Akt levels are significantly increased in *cul1* RNAi mutant ddaC neurons and brains. Finally, knockdown of Akt strongly suppresses the *cul1* RNAi dendrite pruning defects. Since the InR/PI3K/TOR pathway and Akt are important for ddaC dendrite pruning but not for MB axon pruning, it is conceivable that the targets of the Cul1 SCF E3 ligase are divergent in these two modes of neuronal pruning. Interestingly, the SCF-Fbxo40 ligase targets the mammalian IRS for degradation and limits the insulin/InR signaling in skeletal muscle [61], whereas the RING E3 ligase ZNRF1 ubiquitinates Akt to promote Wallerian degeneration of injured dorsal root ganglia neurons, a pruning-like process [62]. Finally, our results also show that the Cul1-based SCF E3 ligase and the InR/PI3K/TOR pathway regulate local caspase activation in ddaC dendrites during pruning. Attenuation of the insulin pathway can downregulate IAP, an inhibitor of caspases, during the differentiation of embryonic chicken lens epithelial cells [63]. It will be of great interest to determine whether global attenuation of InR/PI3K/TOR pathway can similarly downregulate DIAP1, a *Drosophila* homologue of IAP, to locally activate caspases during dendrite pruning.

In summary, we demonstrate that the Cul1-based SCF E3 ligase plays crucial roles in directing two distinct modes of pruning in ddaC and MB γ neurons in *Drosophila*. We further show that the Cul1 SCF E3 ligase inactivates the InR/PI3K/TOR pathway, a key signaling pathway, in order for ddaC neurons to prune their unnecessary larval dendrites during metamorphosis. This study provides a novel link between the SCF E3 ligase and the InR/PI3K/TOR pathway in regulating neuronal pruning. Thus, we open up new avenues for further studies of the E3 ligase in the remodeling and maturation of the developing nervous system, as well as their implications in the pathogenesis of many neurodegenerative diseases.

## Materials and Methods

### Fly Strains

The following fly stocks were used in this study: *cul1^EX^* (C.T. Chien) [37], *UAS-Flag-Cul1* (C.T. Chien) [37], *UAS-cul1^DN^*(generated in this study) [37], *nedd8^AN015^* (C.T. Chien) [37], *roc1a^G1^* [42], *roc1b^dc3^* (R.J. Duronio) [43], *slimb^8^* (B. Limbourg-Bouchon) [64], *slimb^2^, tub-Myc-slimb* (J. Jiang) [36], *Df(3R)swp2^MICAL^*, *UAS-mical^N-ter^* (A. Kolodkin) [65], *mical^15256^* (the Yu lab), *ppk-Gal4* (on Chr II or Chr III; Y. Jan) [66], *UAS-Ci^Cell^* (K. Basler), *UAS-Ci^U^* (K. Basler) [67], *UAS-Dsh^DIX^* (N. Perrimon) [68], *UAS-Smo^Δ661-818^* (J. Jiang) [69], *UAS-Notch^DN^* (d.n.N), *UAS-Notch^CA^* (act.N) (S. Artavanis-Tsakonas) [70], *UAS-Dome^ΔCYT^* (J. Castelli-Gair Hombria) [71], *UAS-PVR^DN^* (P. Rorth) [72], *UAS-Tkv^1ΔGSK^* (M. O'Connor) [73], *UAS-PTEN* (T. Xu) [49], *UAS-TSC1,UAS-TSC2* (T. Xu) [74], *UAS-4E-BP(AA)* (S. Cohen) [51], *ago^3^* (I. Hariharan [57]), *sox14^Δ13^*(the Yu lab), *mical-lacZ* (the Yu lab), *PTEN^c494^* (T, Xu) [49], *PTEN^1^* (C. Wilson) [75], *UAS-CD8::PARP::Venus* [25], *elav-GeneSwitch-Gal4* [40], and *UAS-skpA-RFP* (generated in this study).

The following stocks were obtained from Bloomington stock centre (BSC): *skpA^1^*, *skpA* RNAi #1 (BL29874), *FRT40A Cul3^gft2^,UAS-ptc*, *FRTG13 Cul4^11L^*, *UAS-sgg^S9A^*, *UAS-Bsk^DN^*, *UAS-InR*, *UAS-InR^DN^ (InR^K1409A^)*, *UAS-InR^CA^ (InR^A1325D^)*, *UAS-Arm^S10^*, *UAS-Yki^S168A^*, *UAS-EGFR^DN^*, *UAS-PI3K*, *UAS-PI3K^DN^ (PI3K^D954A^)*, *UAS-PI3K^CA^ (PI3K^CAAX^)*, *UAS-TOR^TED^*, *UAS-S6K^KQ^*, *UAS-S6K^CA^ (S6K^STDETE^)*, *UAS-akt*, *UAS-Rheb*, *akt* RNAi (BL31701 and BL33615), and *Gal4^2–21^*, *201Y-Gal4*, *OK107-Gal4*, *Gal4^109(2)80^*, *elav-Gal4, ppk-CD4-tdTomato*.

The following Stocks were obtained from Vienna Drosophila RNAi Centre (VDRC): *cul1* RNAi #1 (v108558), *cul1* RNAi #2(v42445), *cul1* RNAi lines (v33406 and v33407), *roc1a* RNAi #1 (v106315), *roc1a* RNAi #2 (v32398), *skpA* RNAi #2 (v32790), *skpA* RNAi #3 (v107815), *slimb* RNAi #1 (v107825), and *slimb* RNAi #2 (v34273).

### *UAS-cul1^DN^* and *UAS-skpA-RFP* Transgenes

The GATEWAY pTW vector containing a fragment of the *cul1* cDNA (encoding aa 1–532; Cul1^DN^) or GATEWAY pTRW vector containing a fragment of the *skpA* cDNA were constructed and several transgenic lines were established by the Bestgene Inc.

### Mical Antibody Production

The cDNA fragment corresponding to the last 359 aa of Mical was amplified by PCR and verified by DNA sequencing. The product was expressed using the GST expression vector (pGEX 4T-1, Pharmacia) and the purified protein was used to immunize guinea pigs to generate antibodies against Mical. The specificity of the guinea pig anti-Mical antibody was verified using *mical* mutants.

### MARCM Analysis and Dendrite Imaging/Quantification

We carried out MARCM analysis, dendrite imaging, and quantification as previously described [23].

### Immunohistochemistry and Antibodies

Larval and pupal fillet samples for each set of experiments were processed simultaneously, stained in the same tube, and imaged with the same parameters using Leica SPE confocal microscope. The following antibodies were used for immunohistochemistry at the indicated dilution: guinea pig Anti-Mical (1∶500), mouse polyclonal anti-Sox14 (1∶200), mouse anti-EcR-B1 (1∶50, DDA2.7, DSHB), rabbit anti-GFP (1∶1000, A11122, Invitrogen), mouse anti-FasII (1∶100; 1D4, DSHB), rabbit anti-Akt (1∶500, #4691L, Cell Signaling), and rabbit anti-cleaved PARP (1∶500, 2317-50, Abcam). Cy3- and Cy5-conjugated secondary antibodies (Jackson Laboratories) were used at 1∶400 dilution.

### S2 Cell Culture, Ecdysone Treatment, Transfection, and Western Blotting

We carried out S2 cell culture, ecdysone treatment, and Western blotting as described [23]. For brain extracts, mutant brains were dissected in cold PBS and lysed in 2×SDS protein loading dye and boiled for 5 min, before Western blot analyses. We used mouse anti-Myc (1∶2,000, ab32, Abcam), rabbit anti-Flag (1∶1,000, F-3165, Sigma), rabbit anti-Cullin-1 (C.T. Chien), rabbit anti-SkpA (T. Murphy), rabbit anti-Akt (1∶1,000, #4691L, Cell Signaling), rabbit anti-Akt P-Ser505 (1∶1,000, #4054S, Cell Signaling), and rat anti-HA (1∶1,000, 11867423001, Roche). Flag-Slimb, Flag-Slimb^ΔWD40^, Myc-Slimb, Myc-Slimb^ΔWD40^, Myc-Akt, Flag-Akt, and Flag-Roc1a expression vectors were generated by Gateway cloning and were transfected into S2 cells using Effectene Transfection Reagent (Qiagen). The specificity of all the antibodies was examined in the individual RNAi S2 cells.

### Co-Immunoprecipitation (Co-IP)

Transfected S2 cells or prepupae brains were homogenized with lysis buffer (25 mM Tris pH8/27.5 mM NaCl/20 mM KCl/25 mM sucrose/10 mM EDTA/10 mM EGTA/1 mM DTT/10% (v/v) glycerol/0.5% Nonidet P40) with protease inhibitors (Complete, Boehringer; PMSF 10 µg/ml, Sodium orthovanadate 10 µg/ml) in presence or absence of ecdysone (20E). The supernatants were used for immunoprecipitation with anti-Myc, anti-Flag, or anti-Akt overnight at 4°C, followed by incubation with protein A/G beads (Pierce Chemical Co.) for 2 h. Protein A/G beads were washed four times with cold PBS. Bound proteins were separated by SDS-PAGE and analysed by Western blotting with anti-Myc, anti-Flag, anti-Cullin-1, anti-SkpA, and anti-Akt.

### In Vivo Ubiquitination Assay

S2 cells were transfected with Flag-Slimb, Flag-Slimb^ΔWD40^, Myc-Akt, and pHsp70-hemagglutinin (HA)-ubiquitin (A.Sehgal). At 48 h postransfection, cells were homogenized with the lysis buffer (25 mM Tris pH8/27.5 mM NaCl/20 mM KCl/25 mM sucrose/10 mM EDTA/10 mM EGTA/1 mM DTT/10% (v/v) glycerol/0.5% Nonidet P40) with protease inhibitors (Complete, Boehringer; PMSF 10 µg/ml, Sodium orthovanadate 10 µg/ml). The supernatants were used for immunoprecipitation with anti-Myc, overnight at 4°C, followed by incubation with protein A/G beads (Pierce Chemical Co.) for 2 h. Protein A/G beads were washed four times with cold PBS. Bound proteins were separated by SDS-PAGE and analysed by Western blotting with anti-Myc, anti-HA, and anti-Flag.

### Quantification of ddaC Dendrites

Live confocal images of ddaC neurons expressing *UAS-mCD8-GFP* driven by *ppk-GAL4* were shown at w3L, WP, and 16 h APF. The average number of primary and secondary dendrites attached to soma or total dendritic termini was counted from wild-type and mutant ddaC neurons. The number of samples (*n*) in each group is shown on the bars. Error represents S.E.M. Dorsal is up in all images. The strength of the pruning phenotypes was divided into three levels: mild (less than 5 primary and secondary dendrites), moderate (5–10 primary and secondary dendrites), and strong/severe (>10 primary and secondary dendrites).

### Dissection of Brains, Visualization of Mushroom Body Neurons, and MARCM Mosaic Analysis

Larval, pupal, and adult brains were dissected in PBS and fixed in 4% formaldehyde for 15 min. Brains were washed in PBS+1%Triton X for 3 times for 10 min each. For the clonal analysis study (MARCM), embryos were collected at 6 h interval. The clones were induced in the first instar lavae by applying a 1 h heat shock at 38°C. The antibody to FASII (1D4) was obtained from the Developmental Studies Hybridoma Bank and used at 1∶50. Rabbit anti-GFP antibody was obtained from Invitrogen and used at 1∶1,000. *201Y-Gal4* labels postmitotic γ neurons and a small subset of late-born α/β neurons. The Anti-FasII (1D4) antibody labels α/β neurons strongly and γ neurons weakly. The severity of the axon pruning phenotypes was divided into three levels: weak, strong, and complete, according to a previous study [21].

### Quantification of Immunolabeling

To quantify the immunolabeling intensities, cell nuclei (EcR-B1/Sox14 immunostaining) or whole cell body (Mical immunostaining) were drawn on the appropriate fluorescent channel based on the GFP channel relative cellular localization in ImageJ software. After subtracting the background (Rolling Ball Radius = 30) on the entire image of that channel, we measured the mean grey value in the marked areas in ddaC and ddaE on the same images and calculated their ratios. The ratios were normalized to corresponding average control values and subjected to statistical *t* test for comparison between different conditions (*p<0.05, **p<0.01, ***p<0.001, n.s., not significant). Graphs display the average values of ddaC/ddaE ratios and the standard error of means (S.E.M). *n* is shown on the bars. Insets show the ddaC neurons labeled by *ppk-*GAL4 driven *UAS-mCD8-GFP* expression. Dorsal is up in all images.

To quantify the immunolabeling intensities of Akt, soma/dendrite/axon regions were drawn on the appropriate fluorescent channel based on the GFP channel relative cellular localization in ImageJ software. After subtracting the background, we measured the mean grey value of Akt in the marked areas. The values were normalized to corresponding average control values and subjected to statistical *t* test for comparison between different conditions. Graphs display the average values of normalized Akt expression and the standard error of means (S.E.M). *n* is shown on the bars. Insets show the soma/dendrite/axon labeled by *ppk-GAL4* driven *UAS-mCD8-GFP* expression (*p<0.05, **p<0.01, ***p<0.001, n.s., not significant).

### Rapamycin Treatment

To avoid any developmental delay, wild-type or mutant embryos were collected at 6 h intervals and were reared on standard food to the 3^rd^ instar stage (96 h after egg laying, AEL) before being transferred to the standard culture medium containing 2 µM of Rapamycin dissolved in ethanol (Sigma Aldrich R0395). Larvae were fed in Rapamycin food for approximately 8 h before cessation of feeding. The onset of puparium formation and adult eclosion was not affected by Rapamycin treatment. wL3 larvae were used to quantify the total dendrite termini.

### RU486/Mifepristone Treatment for *elav-GeneSwitch* System

To avoid any developmental delay, wild-type or mutant embryos were collected at 6 h intervals and were reared on standard food to the e3L stage before being transferred to the standard culture medium containing 240 µg/ml mifepristone dissolved in ethanol (Sigma Aldrich M8046). The onset of puparium formation and adult eclosion was not affected by RU486 treatment.

### RNA Isolation and Reverse Transcription

Isolation of RNA from MB γ neurons was accomplished using Laser Capture Microdissection (LCM). Ten-micrometer frozen sections were cut from larval or pupal brains. MB γ neurons labeled by *201Y-GAL4*>mCD8-GFP were microdissected using the Zeiss PALM microbeam microdissection system. Each capture consisted of ∼80 cell bodies, and 30 captures were pooled to obtain each replicate. Total RNA was extracted using the PicoPure RNA isolation kit from Arcturus and subject to reverse transcription using Oligo dT and the SuperScript III First-Stand Synthesis SuperMix (Invitrogen). The genomic DNA was digested by RNase-free DNase (Qiagen).

### Quantitative Real-Time PCR (Q-PCR)

Independent experiments were conducted in triplicates using Maxima SYBR Green/ROX qPCR Master Mix (Fermentas) and 7900HT Fast Real-time PCR system (Applied Biosystems) according to the manufacturer's recommendations. *rp49* (CG7939) was used as an internal control gene. Results were normalized to the controls indicated. Error bars represent standard error of mean (SEM) for four experimental repeats (*n* = 4).

**Primers listed in 5′-to-3′ Sequence:**

*EcR-B1*

CTGCTCATAGCCATCCTGGT

GCGGCCAAGACTTTGTTAAG

*sox14*

GAAAGATCTCCGAGCCACAG

ATCTGGCTCCAAACCATGAA

*mical*

TTGGTGGGCTTCCTTAGATG

GTTCAAACCGAGTCCGAGAG

*cul1*

CCACATGCGAAGAGGTTCTTAT

CAAGGATGGACTTGAGATCTGTC

*uba1*

GATATCCTTCTGTCGGGACTTG

GATATCGGCTTCCGTGAGATAG

*rp49*

GCTTCAAGGGACAGTATCTGATG

GACAATCTCCTTGCGCTTCTT

## Supporting Information

Figure S1 **Cul1 and Nedd8 are required for remodeling of dda sensory neurons.** (A–D) Live confocal images of dda neurons expressing *UAS-mCD8-GFP* at WP, 16 h, or 18 h APF. (A) ddaC neurons overexpressing one copy of *cul1* RNAi #1 or *cul1* RNAi #2 ddaC clones exhibited dendrite pruning defects at 16 h APF. Note: the severity of RNAi phenotype mainly depends on copy number/strength of *ppk-Gal4* drivers as well as *UAS-Dcr2*. Expression level of *ppk-Gal4* (inserted on Chr II) is much stronger than that of *ppk-Gal4* (on Chr III). Thus, the phenotype of *cul1* RNAi #2 in Figure 1C′ is almost double compared to that in Figure S1A due to the presence of two copies of *ppk-Gal4* (Chr II). Please note that the ddaF neuron is occasionally labeled by one copy of *ppk-Gal4* (Chr II) but always by two copies of the driver. (B) While wild-type class I ddaD/ddaE neurons pruned normally at 18 h APF, *cul1^EX^*, and *nedd8^AN015^* MARCM ddaD neurons failed to prune their respective dendrites by 18 h APF. Blue arrowheads point to ddaD neurons, and green arrowheads to ddaE. (C) Wild-type class III ddaF neurons underwent apoptosis by 16 h APF, whereas *cul1^EX^* and *nedd8^AN015^* MARCM ddaF neurons survived by 16 h APF. Purple arrowheads point to ddaF neurons. (D) Similar to *cul1^EX^*, *nedd8^AN015^* MARCM ddaC neurons failed to prune their larval dendrites by 16 h APF. Quantification of the average number of primary and secondary dendrites attached to the soma of mutant ddaC neurons at WP and 16 h APF. Dorsal is up in all images. The number of samples (*n*) in each group is shown on the bars. Error bars represent S.E.M. The scale bars are 50 µm. See genotypes in Text S1.
(TIF)

Figure S2 **Initial dendrite development is not affected in** ***cul1*** **RNAi,** ***skpA*** **RNAi, or** ***roc1a*** **RNAi ddaC neurons.** (A) Live confocal images of ddaC neurons expressing *UAS-mCD8-GFP* at w3L. ddaC neurons overexpressing *cul1* RNAi #2, *skpA* RNAi #2, or *roc1a* RNAi #2 via one copy of *ppk-Gal4* (on Chr II) showed similar w3L dendrite morphology, compared to the wild-type control. Quantification of the average number of dendritic termini of each genotype. The number of samples (*n*) in each group is shown on the bars. Error bars represent S.E.M. n.s., not significant. (B) Live confocal images of ddaC neurons labeled by *ppk-CD4-tdTomato*. RU486 treatment did not affect dendrite pruning in the wild-type ddaC neurons (the far left panels). Using the RU486-inducible Gene-Switch system, inducible expression of *cul1^DN^* resulted in dendrite pruning defects in 16 h APF ddaC neurons, compared to 0% of the noninduced controls. WP dendrite morphology of induced versus noninduced ddaC neurons was similar. Please note that weak dendrite signals at 16 h APF are due to less prominent fluorescence of tdTomato. See genotypes in Text S1.
(TIF)

Figure S3 **Roc1a, SkpA, and Slimb are required for pruning of class IV ddaC neurons.** Live confocal images of ddaC neurons expressing *UAS-mCD8-GFP* at WP or 16 h APF. ddaC neurons expressing one copy of *Roc1a* RNAi #1, two copies of *Roc1a* RNAi #2, one copy of *slimb* RNAi #1, or one copy of *slimb* RNAi #2 exhibited prominent pruning defects. Quantification of the average number of primary and secondary dendrites attached to the soma of mutant ddaC neurons at WP and 16 h APF. Dorsal is up in all images. The number of samples (*n*) in each group is shown on the bars. Error bars represent S.E.M. The scale bars are 50 µm. See genotypes in Text S1.
(TIF)

Figure S4 **Roc1a, SkpA, and Slimb are required for pruning of class I ddaD/E neurons and apoptosis of class III ddaF neurons.** (A and B) Live confocal images of dda neurons expressing *UAS-mCD8-GFP* at WP, 16 h, or 18 h APF. (A) Wild-type class I ddaD/ddaE neurons pruned normally at 18 h APF, whereas *roc1a^G1^* MARCM, *Slimb^8^* MARCM, and *SkpA* RNAi expressing ddaD neurons failed to prune their larval dendrites by 18 h APF. Blue arrowheads point to ddaD neurons, and green arrowheads to ddaE. (B) Wild-type class III ddaF neurons underwent apoptosis by 16 h APF, whereas *roc1a^G1^* and *slimb^8^* MARCM ddaF neurons survived at 16 h APF. Knockdown of SkpA in ddaF neurons via the da neuronal driver *109(2)80-Gal4* driven *skpA* RNAi expression also resulted in failure of ddaF to undergo apoptosis at 18 h APF. Purple arrowheads point to ddaF neurons. Dorsal is up in all images. See genotypes in Text S1.
(TIF)

Figure S5 **Roc1b, Cul3, Ago, and Cul4 are not required for pruning of class IV ddaC neurons.** Similar to wild-type ddaC neurons, *roc1b^dc3^* homozygous mutant, *cul3^gft2^*, *ago^3^*, or *cul4^11L^* MARCM ddaC neurons pruned their dendrites normally at 16 h APF. Dorsal is up in all images. See genotypes in Text S1.
(TIF)

Figure S6 **The Cul1-based SCF E3 ligase is required for MB γ neuron remodelling.** (A–C) Confocal images of MB γ neurons expressing *UAS-mCD8-GFP* driven by *201Y*-*Gal4* at wL3 or 24 h APF in wild-type, *cul1^EX^* MB neuroblast clones expressing the full-length Cul1, *roc1a^G1^* MB neuroblast clone expressing the full-length Roc1a, and *roc1b^dc3^* MB neuroblast mutants. (A) Overexpression of Cul1 in *cul1^EX^* MB or Roc1a in *roc1a^G1^* MB neuroblast clones fully rescued their axon pruning defects. (B) Roc1b is not required for MB axon pruning. (C) *cul^EX^* and *skpA^1^* MB neuroblast clones failed to prune their dendrites at 24 h APF compared to the wild-type control. Red arrowheads point to the unpruned dendrites. The scale bars are 50 µm. See genotypes in Text S1.
(TIF)

Figure S7 **The Cul1-based SCF E3 ligase is not required for** ***EcR-B1/sox14*** **expression or** ***mical*** **transcription.** (A) Confocal images of ddaC neurons expressing *UAS-mCD8-GFP* driven by *ppk*-*Gal4* at WP immunostained for LacZ (shown in red). The *mical-lacZ* reporter drives upregulation of LacZ expression at WP under a *mical* enhancer. *mical* transcription was not affected in *cul1* RNAi or *skpA* RNAi ddaC neurons at WP. (B) Quantification of immunostaining for EcR-B1 and Sox14. The graphs display the average values of ddaC/ddaE ratios and S.E.M. *n* is shown on the bars. EcR-B1 and Sox14 expression in wild-type, *cul1^EX^* MARCM, *roc1a^G1^* MARCM, *skpA* RNAi, *slimb^8^* MARCM, or *nedd8^AN015^* MARCM ddaC neurons remained largely unchanged. Dorsal is up in all images. n.s., not significant. The scale bar in (A) is 20 µm. See genotypes in Text S1.
(TIF)

Figure S8 **The Cul1-based SCF E3 ligase acts downstream of Sox14 but in parallel to Mical to mediate dendrite pruning.** *mical* ddaC neurons displayed a pruning defect with the average of 5.3 primary and secondary dendrites attached to the soma at 16 h APF. Knockdown of *cul1* and *roc1a* in the *mical* background with their respective RNAi lines significantly enhanced the *mical* pruning defects. Knockdown of *cul1* with *cul1* RNAi #2 in ddaC neurons displayed moderate pruning defect with the average of 4.1 primary and secondary dendrites attached to the soma. *sox14* ddaC neurons displayed a severe pruning defect with the average of 14.7 primary and secondary dendrites attached to the soma at 16 h APF . Knockdown of *cul1* in *sox14* mutant resulted in no enhancement of pruning defect, compared to *sox14* mutant alone. Quantification of the average number of primary and secondary dendrites attached to the soma of mutant ddaC neurons at WP. The number of samples (*n*) in each group is shown on the bars. Error bars represent S.E.M. Dorsal is up in all images. ***p<0.001. n.s., not significant. Scale bar is 20 µm. See genotypes in Text S1.
(TIF)

Figure S9 **ddaC neurons of various double mutant combinations have a similar number of major dendrites attached to their somas at WP stage.** Live confocal images of ddaC neurons expressing *UAS-mCD8-GFP* driven by *ppk*-*Gal4* at WP. Knockdown of Cul1 or Roc1a in the *mical* background did not significantly alter the ddaC WP morphology. *sox14* mutant, *cul1* RNAi, *cul1* RNAi, and *sox14* double mutant ddaC neurons displayed similar elaboration of primary and secondary dendrites at WP stage. Quantification of the average number of primary and secondary dendrites attached to the soma of mutant ddaC neurons at WP. The number of samples (*n*) in each group is shown on the bars. Error bars represent S.E.M. Dorsal is up in all images. Scale bar is 20 µm. See genotypes in Text S1.
(TIF)

Figure S10 **Activation of the Insulin pathway in ddaC neurons results in ddaC dendrite pruning defects.** Live confocal images of ddaC neurons expressing *UAS-mCD8-GFP* driven by *ppk*-*Gal4* at 16 h APF. Inactivation of Hh, Wg, Insulin, or Notch pathways via expression of their respective repressors in ddaC neurons did not result in any pruning defects. Activation of the Insulin signaling via *InR^CA^*, but not activation of Hh, Wg, or Notch signalling in ddaC neurons, led to a notable pruning defect at 16 h APF. Quantification of the average number of primary and secondary dendrites attached to the soma of mutant ddaC neurons at WP and 16 h APF. The number of samples (*n*) in each group is shown on the bars. Error bars represent S.E.M. Dorsal is up in all images. ***p<0.001. Scale bar is 20 µm. See genotypes in Text S1.
(TIF)

Figure S11 **Activation of the Insulin pathway in ddaC neurons enhances dendrite pruning defects in** ***cul1^DN^*** **expressing ddaC neurons.** Co-expression of *InR^CA^*, but not other activators of various pathways examined, significantly enhanced *cul1^DN^*-mediated pruning defects in ddaC neurons. Quantification of the average number of primary and secondary dendrites attached to the soma of mutant ddaC neurons at 16 h APF. The number of samples (*n*) in each group is shown on the bars. Error bars represent S.E.M. Dorsal is up in all images. ***p<0.001. n.s., not significant. Scale bar is 20 µm. See genotypes in Text S1.
(TIF)

Figure S12 **Attenuation of other signaling pathways is unable to rescue** ***cul1*** **RNAi-mediated dendrite pruning defect.** (A) Attenuation of JNK, JAK/STAT, Hippo, EGFR, PVR, and Dpp pathways in ddaC neurons by co-expression of *Bsk^DN^* (JNK), *Fos^DN^* (JNK), *Dome^ΔCYT^* (JAK/STAT), *Yki^S168A^* (Hippo), *Egfr^DN^* (EGFR), *Pvr^DN^* (PVR), or *TkV^DN^* (Dpp) with *cul1* RNAi was unable to rescue *cul1* RNAi-mediated dendrite pruning defects, similar to the *mical^N-ter^* control. Quantification of the average number of primary and secondary dendrites attached to the soma of ddaC neurons at WP and 16 h APF. (B) Quantification of the average number of WP primary and secondary dendrites attached to various genotypes of ddaC neurons in Figure 6A–H. The number of samples (*n*) in each group is shown on the bars. ** *p*<0.01. n.s., not significant. Error bars represent S.E.M. See genotypes in Text S1.
(TIF)

Figure S13 **Attenuation of PI3K/TOR signaling pathway is sufficient to rescue** ***cul1^DN^*** **-mediated ddaC dendrite pruning defect.** Inhibition of the PI3K/TOR pathway was also able to suppress *cul1^DN^* pruning defects. While ddaC neurons co-expressing nonfunctional *mical^N-ter^* with *cul1^DN^* displayed apparent pruning defects, co-expression of *PI3K^DN^*, *PTEN*, *InR^DN^*, *TSC1/TSC2*, *TOR^TED^*, *4E-BP(AA)*, or *S6K^KQ^* significantly suppressed *cul1^DN^* pruning defects. Quantification of the average number of primary and secondary dendrites attached to the soma of mutant ddaC neurons at 16 h APF. The number of samples (*n*) in each group is shown on the bars. Error bars represent S.E.M. Dorsal is up in all images. ***p<0.001. Scale bar is 20 µm. See genotypes in Text S1.
(TIF)

Figure S14 **Pharmacological attenuation of the InR/PI3K/TOR signaling significantly suppresses the dendrite pruning defects in** ***cul1*** **RNAi ddaC neurons.** (A) Rapamycin treatment did not affect initial dendrite development in *cul1* RNAi or *mical* RNAi-expressing ddaC neurons. Quantifications of the total dendritic termini of Rapamycin-treated or nontreated mutant ddaC neurons at w3L. (B) Rapamycin treatment, similar to the effects of InR^DN^ and PI3K^DN^, significantly suppressed the dendrite pruning defects in *cul1* RNAi ddaC neurons, but not in *mical* RNAi ddaC neurons. Quantification of the average number of primary and secondary dendrites attached to the soma of mutant ddaC neurons at 16 h APF. The number of samples (*n*) in each group is shown on the bars. ***p<0.001. n.s., not significant. Error bars represent S.E.M. See genotypes in Text S1.
(TIF)

Figure S15 **Attenuation of PI3K/TOR signaling does not alter the number of major dendrites attached to ddaC somas at WP stage.** (A) Co-expression of nonfunctional *mical^N-ter^*, *InR^DN^*, *PI3K^DN^*, *PTEN*, *TOR^TED^*, *TSC1/TSC2*, *S6K^KQ^*, *4E-BP(AA)*, or *akt* RNAi with *cul1* RNAi resulted in normal elaboration of primary and secondary dendrites in ddaC neurons at the WP stage. (B) Co-expression of nonfunctional *mical^N-ter^*, *InR^DN^*, *PI3K^DN^*, *PTEN*, *TOR^TED^*, *TSC1/TSC2*, *S6K^KQ^*, and *4E-BP(AA)* with *cul1^DN^* resulted in normal elaboration of primary and secondary dendrites in ddaC neurons at the WP stage. Quantification of the average number of primary and secondary dendrites attached to the soma of mutant ddaC neurons at 16 h APF. The number of samples (*n*) in each group is shown on the bars. n.s., not significant. Error bars represent S.E.M. See genotypes in Text S1.
(TIF)

Figure S16 **Specific effects of the InR/PI3K/TOR pathway on ddaC dendrite pruning.** (A and B) Live confocal images of ddaC neurons expressing *UAS-mCD8-GFP* driven by *ppk*-*Gal4* at 12.5 h or 16 h APF. (A) While all ddaC neurons co-expressing nonfunctional *mical^N-ter^* with *cul1* RNAi failed to sever the proximal regions of their dorsal dendrite branch at 12.5 h APF, proximal severing of dorsal dendrite branches was observed in *cul1* RNAi ddaC neurons co-expressing *InR^DN^*, *PI3K^DN^*, *PTEN*, *TOR^TED^*, *TSC1/TSC2*, *S6K^KQ^*, or *4E-BP(AA)*. Empty red arrowheads point to proximal severing of the dorsal dendrite branches. (B) The expression of nonfunctional *mical^N-ter^* control, *InR^DN^*, *PI3K^DN^*, *PTEN*, *TOR^TED^*, *TSC1/TSC2*, *S6K^KQ^*, or *4E-BP(AA)* was unable to rescue the dendrite pruning defects in *mical* mutant ddaC neurons. Quantification of the average number of primary and secondary dendrites attached to the soma of mutant ddaC neurons at WP and 16 h APF. The number of samples (*n*) in each group is shown on the bars. Error bars represent S.E.M. n.s., not significant. Dorsal is up in all images. The scale bar is 50 µm. See genotypes in Text S1.
(TIF)

Figure S17 **Overexpressed SkpA-RFP and Akt are localized uniformly throughout the ddaC neurons.** SkpA-RFP and Akt were labeled in red, and mCD8-GFP in green. White arrowheads and arrows point to ddaC somas and axons, respectively. The scale bars are 50 µm. Dorsal is up in all images. See genotypes in Text S1.
(TIF)

Figure S18 **Specificity of** ***cul1*** **knockdown in the brain.** Akt is unable to associate with another F-box containing protein, Ago. (A) Cul1 protein levels were reduced via *cul1* RNAi lines #1 using a pan-neuronal driver *elav-Gal4*. See genotypes in Text S1. (B) Akt did not associate with another F-box-containing protein Ago in S2 cells cotransfected with Flag-Ago and Myc-Akt.
(TIF)

Figure S19 **Activation of the InR/PI3K/TOR pathway is sufficient to inhibit ddaC dendrite pruning but not MB γ axon pruning.** (A, B, and D) Live confocal images of dda neurons expressing *UAS-mCD8-GFP* driven at WP, 16 h APF, or 18 h APF. (A) Activation of the InR/PI3K/TOR pathway by the expression of *Rheb* or S6K^STDETE^ in ddaC neurons resulted in ddaC dendrite pruning defects. (B) *pten^1^* MARCM ddaC neurons exhibited dendrite pruning defects at 16 h APF; similarly, its ddaD neurons also failed to prune at 18 h APF. Quantification of the average number of primary and secondary dendrites attached to the soma of mutant ddaC neurons at WP or 16 h APF. The number of samples (*n*) in each group is shown on the bars. Error bars represent S.E.M. Dorsal is up in all images. (C) Confocal images of MB γ neurons expressing *UAS-mCD8-GFP* driven by *201Y*-*Gal4* at 24 h APF. Similar to wild-type MB γ neurons, *pten^c494^* MB γ neurons MARCM clones and *InR^CA^*-expressing MB γ neurons pruned their dorsal and medial axon branches by 24 h APF. (D) Expression of *akt* RNAi in *InR^CA^*-expressing ddaC neurons fully suppressed *InR^CA^*-mediated dendrite pruning defect. The scale bars are 50 µm. See genotypes in Text S1.
(TIF)

Figure S20 **Activation of the InR/PI3K/TOR pathway enhances** ***cul1^DN^*** **-mediated dendrite pruning defect.** Activation of the InR/PI3K/TOR pathway by *InR^CA^*, *PI3K^CA^*, *Rheb*, or *S6K^STDETE^* in *cul1^DN^* ddaC neurons did not affect normal dendrite arborization at WP. Co-expression of *InR^CA^*, *PI3K^CA^*, *Rheb*, or *S6K^STDETE^* with *cul1^DN^* dramatically enhanced the pruning defects, compared to that of nonfunctional *mical^N-ter^* control. Quantification of the average number of primary and secondary dendrites attached to the soma of mutant ddaC neurons at WP or 16 h APF. The number of samples (*n*) in each group is shown on the bars. Error bars represent S.E.M. ***p<0.001. n.s., not significant. Dorsal is up in all images. The scale bar is 50 µm. See genotypes in Text S1.
(TIF)

Figure S21 **Activation of InR/PI3K/TOR pathway does not affect EcR-B1, Sox14, and Mical expression.** (A and B) Activation of the InR/PI3K/TOR pathway via I*nR^CA^*, *PI3K^CA^*, *pten^c494^* MARCM, or *pten^1^* MARCM in ddaC neurons did not affect EcR-B1, Sox14, and Mical expression at WP stage. Quantification of immunostaining for EcR-B1, Sox14, and Mical levels was performed as described in Materials and Methods. Graphs display the average values of ddaC/ddaE ratios. Error bars represent S.E.M. *n* is shown on the bars. n.s., not significant. See genotypes in Text S1.
(TIF)

Figure S22 **The SCF ligase and the InR/PI3K/TOR pathway regulate dendrite pruning in ddaC neurons at least in part by promoting local caspase activation in the dendrites.** Confocal images of ddaC neurons expressing the caspase reporter construct CD8::PARP::VENUS at 6 h APF. While cleaved PARP marked in white bracket was readily detected in the wild-type ddaC neurons at 6 h APF, ddaC neurons expressing *cul1* RNAi or *InR^CA^* failed to activate the caspase activity at 6 h APF. White arrowhead points to ddaC somas. The scale bar is 20 µm. See genotypes in Text S1.
(TIF)

Text S1 **List of fly strains.** Various genotypes were used in the main and supplementary figures.
(DOCX)

## Abbreviations

4E-BP: eukaryotic translation initiation factor 4E binding protein
APF: after puparium formation
CNS: central nervous system
Cul1: Cullin1
dda: dorsal dendrite arborization
DN: dominant negative form
ecdysone: 20-hydroxyecdysone
EcR: Ecdysone Receptor
eL3: early 3^rd^ instar
InR: Insulin Receptor
LCM: Laser Capture Microdissection
MB: mushroom body
PNS: peripheral nervous system
PTEN: the Phosphatase and tensin homologue
Q-PCR: Quantitative real-time PCR
Rheb: Ras homologue enriched in brain
RNAi: RNA interference
S6K: p70 ribosomal protein S6 kinase
SCF: Skp1-Cullin-F-box
Slimb: Supernumerary limbs
TOR: Target of Rapamycin
UPS: ubiquitin-proteasome system
Usp: Ultraspiracle
wL3: wandering 3^rd^ instar
WP: white prepupal

## References

[1] Luo L, O'Leary DD (2005) Axon retraction and degeneration in development and disease. Annu Rev Neurosci 28: 127–156. 16022592 10.1146/annurev.neuro.28.061604.135632

[2] Weeks JC, Truman JW (1985) Independent steroid control of the fates of motoneurons and their muscles during insect metamorphosis. J Neurosci 5: 2290–2300. 4020438 10.1523/JNEUROSCI.05-08-02290.1985 PMC6565275

[3] Truman JW (1990) Metamorphosis of the central nervous system of Drosophila. J Neurobiol 21: 1072–1084. 1979610 10.1002/neu.480210711

[4] O'Leary DD, Koester SE (1993) Development of projection neuron types, axon pathways, and patterned connections of the mammalian cortex. Neuron 10: 991–1006. 8318235 10.1016/0896-6273(93)90049-w

[5] Lichtman JW, Colman H (2000) Synapse elimination and indelible memory. Neuron 25: 269–278. 10719884 10.1016/s0896-6273(00)80893-4

[6] Tapia JC, Wylie JD, Kasthuri N, Hayworth KJ, Schalek R, et al (2012) Pervasive synaptic branch removal in the Mammalian neuromuscular system at birth. Neuron 74: 816–829. 22681687 10.1016/j.neuron.2012.04.017

[7] Lee T, Marticke S, Sung C, Robinow S, Luo L (2000) Cell-autonomous requirement of the USP/EcR-B ecdysone receptor for mushroom body neuronal remodeling in Drosophila. Neuron 28: 807–818. 11163268 10.1016/s0896-6273(00)00155-0

[8] Marin EC, Watts RJ, Tanaka NK, Ito K, Luo L (2005) Developmentally programmed remodeling of the Drosophila olfactory circuit. Development 132: 725–737. 15659487 10.1242/dev.01614

[9] Brown HL, Cherbas L, Cherbas P, Truman JW (2006) Use of time-lapse imaging and dominant negative receptors to dissect the steroid receptor control of neuronal remodeling in Drosophila. Development 133: 275–285. 16354717 10.1242/dev.02191

[10] Singh AP, VijayRaghavan K, Rodrigues V (2010) Dendritic refinement of an identified neuron in the Drosophila CNS is regulated by neuronal activity and Wnt signaling. Development 137: 1351–1360. 20223760 10.1242/dev.044131

[11] Williams DW, Truman JW (2005) Cellular mechanisms of dendrite pruning in Drosophila: insights from in vivo time-lapse of remodeling dendritic arborizing sensory neurons. Development 132: 3631–3642. 16033801 10.1242/dev.01928

[12] Kuo CT, Jan LY, Jan YN (2005) Dendrite-specific remodeling of Drosophila sensory neurons requires matrix metalloproteases, ubiquitin-proteasome, and ecdysone signaling. Proc Natl Acad Sci U S A 102: 15230–15235. 16210248 10.1073/pnas.0507393102 PMC1242853

[13] Watts RJ, Hoopfer ED, Luo L (2003) Axon pruning during Drosophila metamorphosis: evidence for local degeneration and requirement of the ubiquitin-proteasome system. Neuron 38: 871–885. 12818174 10.1016/s0896-6273(03)00295-2

[14] Watts RJ, Schuldiner O, Perrino J, Larsen C, Luo L (2004) Glia engulf degenerating axons during developmental axon pruning. Curr Biol 14: 678–684. 15084282 10.1016/j.cub.2004.03.035

[15] Awasaki T, Ito K (2004) Engulfing action of glial cells is required for programmed axon pruning during Drosophila metamorphosis. Curr Biol 14: 668–677. 15084281 10.1016/j.cub.2004.04.001

[16] Schubiger M, Wade AA, Carney GE, Truman JW, Bender M (1998) Drosophila EcR-B ecdysone receptor isoforms are required for larval molting and for neuron remodeling during metamorphosis. Development 125: 2053–2062. 9570770 10.1242/dev.125.11.2053

[17] Zheng X, Wang J, Haerry TE, Wu AY, Martin J, et al (2003) TGF-beta signaling activates steroid hormone receptor expression during neuronal remodeling in the Drosophila brain. Cell 112: 303–315. 12581521 10.1016/s0092-8674(03)00072-2

[18] Awasaki T, Huang Y, O'Connor MB, Lee T (2011) Glia instruct developmental neuronal remodeling through TGF-beta signaling. Nat Neurosci 14: 821–823. 21685919 10.1038/nn.2833 PMC3337551

[19] Pauli A, Althoff F, Oliveira RA, Heidmann S, Schuldiner O, et al (2008) Cell-type-specific TEV protease cleavage reveals cohesin functions in Drosophila neurons. Dev Cell 14: 239–251. 18267092 10.1016/j.devcel.2007.12.009 PMC2258333

[20] Schuldiner O, Berdnik D, Levy JM, Wu JS, Luginbuhl D, et al (2008) piggyBac-based mosaic screen identifies a postmitotic function for cohesin in regulating developmental axon pruning. Dev Cell 14: 227–238. 18267091 10.1016/j.devcel.2007.11.001 PMC2268086

[21] Boulanger A, Clouet-Redt C, Farge M, Flandre A, Guignard T, et al (2011) ftz-f1 and Hr39 opposing roles on EcR expression during Drosophila mushroom body neuron remodeling. Nat Neurosci 14: 37–44. 21131955 10.1038/nn.2700

[22] Kirilly D, Wong JJ, Lim EK, Wang Y, Zhang H, et al (2011) Intrinsic epigenetic factors cooperate with the steroid hormone ecdysone to govern dendrite pruning in Drosophila. Neuron 72: 86–100. 21982371 10.1016/j.neuron.2011.08.003

[23] Kirilly D, Gu Y, Huang Y, Wu Z, Bashirullah A, et al (2009) A genetic pathway composed of Sox14 and Mical governs severing of dendrites during pruning. Nat Neurosci 12: 1497–1505. 19881505 10.1038/nn.2415 PMC3101876

[24] Kuo CT, Zhu S, Younger S, Jan LY, Jan YN (2006) Identification of E2/E3 ubiquitinating enzymes and caspase activity regulating Drosophila sensory neuron dendrite pruning. Neuron 51: 283–290. 16880123 10.1016/j.neuron.2006.07.014

[25] Williams DW, Kondo S, Krzyzanowska A, Hiromi Y, Truman JW (2006) Local caspase activity directs engulfment of dendrites during pruning. Nat Neurosci 9: 1234–1236. 16980964 10.1038/nn1774

[26] Lee HH, Jan LY, Jan YN (2009) Drosophila IKK-related kinase Ik2 and Katanin p60-like 1 regulate dendrite pruning of sensory neuron during metamorphosis. Proc Natl Acad Sci U S A 106: 6363–6368. 19329489 10.1073/pnas.0902051106 PMC2661847

[27] Glickman MH, Ciechanover A (2002) The ubiquitin-proteasome proteolytic pathway: destruction for the sake of construction. Physiol Rev 82: 373–428. 11917093 10.1152/physrev.00027.2001

[28] Ding M, Shen K (2008) The role of the ubiquitin proteasome system in synapse remodeling and neurodegenerative diseases. Bioessays 30: 1075–1083. 18937340 10.1002/bies.20843 PMC3095215

[29] Ciechanover A, Brundin P (2003) The ubiquitin proteasome system in neurodegenerative diseases: sometimes the chicken, sometimes the egg. Neuron 40: 427–446. 14556719 10.1016/s0896-6273(03)00606-8

[30] Zhai Q, Wang J, Kim A, Liu Q, Watts R, et al (2003) Involvement of the ubiquitin-proteasome system in the early stages of wallerian degeneration. Neuron 39: 217–225. 12873380 10.1016/s0896-6273(03)00429-x

[31] Deshaies RJ (1999) SCF and Cullin/Ring H2-based ubiquitin ligases. Annu Rev Cell Dev Biol 15: 435–467. 10611969 10.1146/annurev.cellbio.15.1.435

[32] Collins CA, Wairkar YP, Johnson SL, DiAntonio A (2006) Highwire restrains synaptic growth by attenuating a MAP kinase signal. Neuron 51: 57–69. 16815332 10.1016/j.neuron.2006.05.026

[33] Nakata K, Abrams B, Grill B, Goncharov A, Huang X, et al (2005) Regulation of a DLK-1 and p38 MAP kinase pathway by the ubiquitin ligase RPM-1 is required for presynaptic development. Cell 120: 407–420. 15707898 10.1016/j.cell.2004.12.017

[34] Liao EH, Hung W, Abrams B, Zhen M (2004) An SCF-like ubiquitin ligase complex that controls presynaptic differentiation. Nature 430: 345–350. 15208641 10.1038/nature02647

[35] Ding M, Chao D, Wang G, Shen K (2007) Spatial regulation of an E3 ubiquitin ligase directs selective synapse elimination. Science 317: 947–951. 17626846 10.1126/science.1145727

[36] Jiang J, Struhl G (1998) Regulation of the Hedgehog and Wingless signalling pathways by the F-box/WD40-repeat protein Slimb. Nature 391: 493–496. 9461217 10.1038/35154

[37] Ou CY, Lin YF, Chen YJ, Chien CT (2002) Distinct protein degradation mechanisms mediated by Cul1 and Cul3 controlling Ci stability in Drosophila eye development. Genes Dev 16: 2403–2414. 12231629 10.1101/gad.1011402 PMC187440

[38] Wu JT, Lin HC, Hu YC, Chien CT (2005) Neddylation and deneddylation regulate Cul1 and Cul3 protein accumulation. Nat Cell Biol 7: 1014–1020. 16127432 10.1038/ncb1301

[39] Voigt J, Papalopulu N (2006) A dominant-negative form of the E3 ubiquitin ligase Cullin-1 disrupts the correct allocation of cell fate in the neural crest lineage. Development 133: 559–568. 16396913 10.1242/dev.02201

[40] Osterwalder T, Yoon KS, White BH, Keshishian H (2001) A conditional tissue-specific transgene expression system using inducible GAL4. Proc Natl Acad Sci U S A 98: 12596–12601. 11675495 10.1073/pnas.221303298 PMC60099

[41] Lee T, Luo L (1999) Mosaic analysis with a repressible cell marker for studies of gene function in neuronal morphogenesis. Neuron 22: 451–461. 10197526 10.1016/s0896-6273(00)80701-1

[42] Noureddine MA, Donaldson TD, Thacker SA, Duronio RJ (2002) Drosophila Roc1a encodes a RING-H2 protein with a unique function in processing the Hh signal transducer Ci by the SCF E3 ubiquitin ligase. Dev Cell 2: 757–770. 12062088 10.1016/s1534-5807(02)00164-8

[43] Donaldson TD, Noureddine MA, Reynolds PJ, Bradford W, Duronio RJ (2004) Targeted disruption of Drosophila Roc1b reveals functional differences in the Roc subunit of Cullin-dependent E3 ubiquitin ligases. Mol Biol Cell 15: 4892–4903. 15331761 10.1091/mbc.E04-03-0180 PMC524738

[44] Murphy TD (2003) Drosophila skpA, a component of SCF ubiquitin ligases, regulates centrosome duplication independently of cyclin E accumulation. J Cell Sci 116: 2321–2332. 12730292 10.1242/jcs.00463

[45] Ho MS, Tsai PI, Chien CT (2006) F-box proteins: the key to protein degradation. J Biomed Sci 13: 181–191. 16463014 10.1007/s11373-005-9058-2

[46] Hoopfer ED, Penton A, Watts RJ, Luo L (2008) Genomic analysis of Drosophila neuronal remodeling: a role for the RNA-binding protein Boule as a negative regulator of axon pruning. J Neurosci 28: 6092–6103. 18550751 10.1523/JNEUROSCI.0677-08.2008 PMC2713105

[47] Neufeld TP (2003) Body building: regulation of shape and size by PI3K/TOR signaling during development. Mech Dev 120: 1283–1296. 14623438 10.1016/j.mod.2003.07.003

[48] Leevers SJ, Weinkove D, MacDougall LK, Hafen E, Waterfield MD (1996) The Drosophila phosphoinositide 3-kinase Dp110 promotes cell growth. EMBO J 15: 6584–6594. 8978685 PMC452483

[49] Huang H, Potter CJ, Tao W, Li DM, Brogiolo W, et al (1999) PTEN affects cell size, cell proliferation and apoptosis during Drosophila eye development. Development 126: 5365–5372. 10556061 10.1242/dev.126.23.5365

[50] Hennig KM, Neufeld TP (2002) Inhibition of cellular growth and proliferation by dTOR overexpression in Drosophila. Genesis 34: 107–110. 12324961 10.1002/gene.10139

[51] Teleman AA, Chen YW, Cohen SM (2005) 4E-BP functions as a metabolic brake used under stress conditions but not during normal growth. Genes Dev 19: 1844–1848. 16103212 10.1101/gad.341505 PMC1186183

[52] Barcelo H, Stewart MJ (2002) Altering Drosophila S6 kinase activity is consistent with a role for S6 kinase in growth. Genesis 34: 83–85. 12324955 10.1002/gene.10132

[53] Wittwer F, Jaquenoud M, Brogiolo W, Zarske M, Wustemann P, et al (2005) Susi, a negative regulator of Drosophila PI3-kinase. Dev Cell 8: 817–827. 15935772 10.1016/j.devcel.2005.04.002

[54] Stocker H, Andjelkovic M, Oldham S, Laffargue M, Wymann MP, et al (2002) Living with lethal PIP3 levels: viability of flies lacking PTEN restored by a PH domain mutation in Akt/PKB. Science 295: 2088–2091. 11872800 10.1126/science.1068094

[55] DiAntonio A, Haghighi AP, Portman SL, Lee JD, Amaranto AM, et al (2001) Ubiquitination-dependent mechanisms regulate synaptic growth and function. Nature 412: 449–452. 11473321 10.1038/35086595

[56] Hu J, Zacharek S, He YJ, Lee H, Shumway S, et al (2008) WD40 protein FBW5 promotes ubiquitination of tumor suppressor TSC2 by DDB1-CUL4-ROC1 ligase. Genes Dev 22: 866–871. 18381890 10.1101/gad.1624008 PMC2279197

[57] Moberg KH, Bell DW, Wahrer DC, Haber DA, Hariharan IK (2001) Archipelago regulates Cyclin E levels in Drosophila and is mutated in human cancer cell lines. Nature 413: 311–316. 11565033 10.1038/35095068

[58] Bader M, Arama E, Steller H (2010) A novel F-box protein is required for caspase activation during cellular remodeling in Drosophila. Development 137: 1679–1688. 20392747 10.1242/dev.050088 PMC2860250

[59] Suzuki M, Setsuie R, Wada K (2009) Ubiquitin carboxyl-terminal hydrolase l3 promotes insulin signaling and adipogenesis. Endocrinology 150: 5230–5239. 19837878 10.1210/en.2009-0332

[60] Colombani J, Bianchini L, Layalle S, Pondeville E, Dauphin-Villemant C, et al (2005) Antagonistic actions of ecdysone and insulins determine final size in Drosophila. Science 310: 667–670. 16179433 10.1126/science.1119432

[61] Shi J, Luo L, Eash J, Ibebunjo C, Glass DJ (2011) The SCF-Fbxo40 complex induces IRS1 ubiquitination in skeletal muscle, limiting IGF1 signaling. Dev Cell 21: 835–847. 22033112 10.1016/j.devcel.2011.09.011

[62] Wakatsuki S, Saitoh F, Araki T (2011) ZNRF1 promotes Wallerian degeneration by degrading AKT to induce GSK3B-dependent CRMP2 phosphorylation. Nat Cell Biol 13: 1415–1423. 22057101 10.1038/ncb2373

[63] Basu S, Rajakaruna S, Menko AS (2012) Insulin-like growth factor receptor-1 and nuclear factor kappaB are crucial survival signals that regulate caspase-3-mediated lens epithelial cell differentiation initiation. J Biol Chem 287: 8384–8397. 22275359 10.1074/jbc.M112.341586 PMC3381865

[64] Miletich I, Limbourg-Bouchon B (2000) Drosophila null slimb clones transiently deregulate Hedgehog-independent transcription of wingless in all limb discs, and induce decapentaplegic transcription linked to imaginal disc regeneration. Mech Dev 93: 15–26. 10781936 10.1016/s0925-4773(00)00256-2

[65] Terman JR, Mao T, Pasterkamp RJ, Yu HH, Kolodkin AL (2002) MICALs, a family of conserved flavoprotein oxidoreductases, function in plexin-mediated axonal repulsion. Cell 109: 887–900. 12110185 10.1016/s0092-8674(02)00794-8

[66] Grueber WB, Jan LY, Jan YN (2003) Different levels of the homeodomain protein cut regulate distinct dendrite branching patterns of Drosophila multidendritic neurons. Cell 112: 805–818. 12654247 10.1016/s0092-8674(03)00160-0

[67] Methot N, Basler K (1999) Hedgehog controls limb development by regulating the activities of distinct transcriptional activator and repressor forms of Cubitus interruptus. Cell 96: 819–831. 10102270 10.1016/s0092-8674(00)80592-9

[68] Axelrod JD, Miller JR, Shulman JM, Moon RT, Perrimon N (1998) Differential recruitment of Dishevelled provides signaling specificity in the planar cell polarity and Wingless signaling pathways. Genes Dev 12: 2610–2622. 9716412 10.1101/gad.12.16.2610 PMC317102

[69] Zhao Y, Tong C, Jiang J (2007) Hedgehog regulates smoothened activity by inducing a conformational switch. Nature 450: 252–258. 17960137 10.1038/nature06225

[70] Go MJ, Eastman DS, Artavanis-Tsakonas S (1998) Cell proliferation control by Notch signaling in Drosophila development. Development 125: 2031–2040. 9570768 10.1242/dev.125.11.2031

[71] Brown S, Hu N, Hombria JC (2001) Identification of the first invertebrate interleukin JAK/STAT receptor, the Drosophila gene domeless. Curr Biol 11: 1700–1705. 11696329 10.1016/s0960-9822(01)00524-3

[72] Duchek P, Somogyi K, Jekely G, Beccari S, Rorth P (2001) Guidance of cell migration by the Drosophila PDGF/VEGF receptor. Cell 107: 17–26. 11595182 10.1016/s0092-8674(01)00502-5

[73] Haerry TE, Khalsa O, O'Connor MB, Wharton KA (1998) Synergistic signaling by two BMP ligands through the SAX and TKV receptors controls wing growth and patterning in Drosophila. Development 125: 3977–3987. 9735359 10.1242/dev.125.20.3977

[74] Potter CJ, Huang H, Xu T (2001) Drosophila Tsc1 functions with Tsc2 to antagonize insulin signaling in regulating cell growth, cell proliferation, and organ size. Cell 105: 357–368. 11348592 10.1016/s0092-8674(01)00333-6

[75] Goberdhan DC, Paricio N, Goodman EC, Mlodzik M, Wilson C (1999) Drosophila tumor suppressor PTEN controls cell size and number by antagonizing the Chico/PI3-kinase signaling pathway. Genes Dev 13: 3244–3258. 10617573 10.1101/gad.13.24.3244 PMC317204

