# Supplementary figures and images for: A Cullin1-Based SCF E3 Ubiquitin Ligase Targets the InR/PI3K/TOR Pathway to Regulate Neuronal Pruning
Source: PLoS Biol. 2013 Sep 17;11(9):e1001657. doi: 10.1371/journal.pbio.1001657 (PMC3775723; doi:10.1371/journal.pbio.1001657)

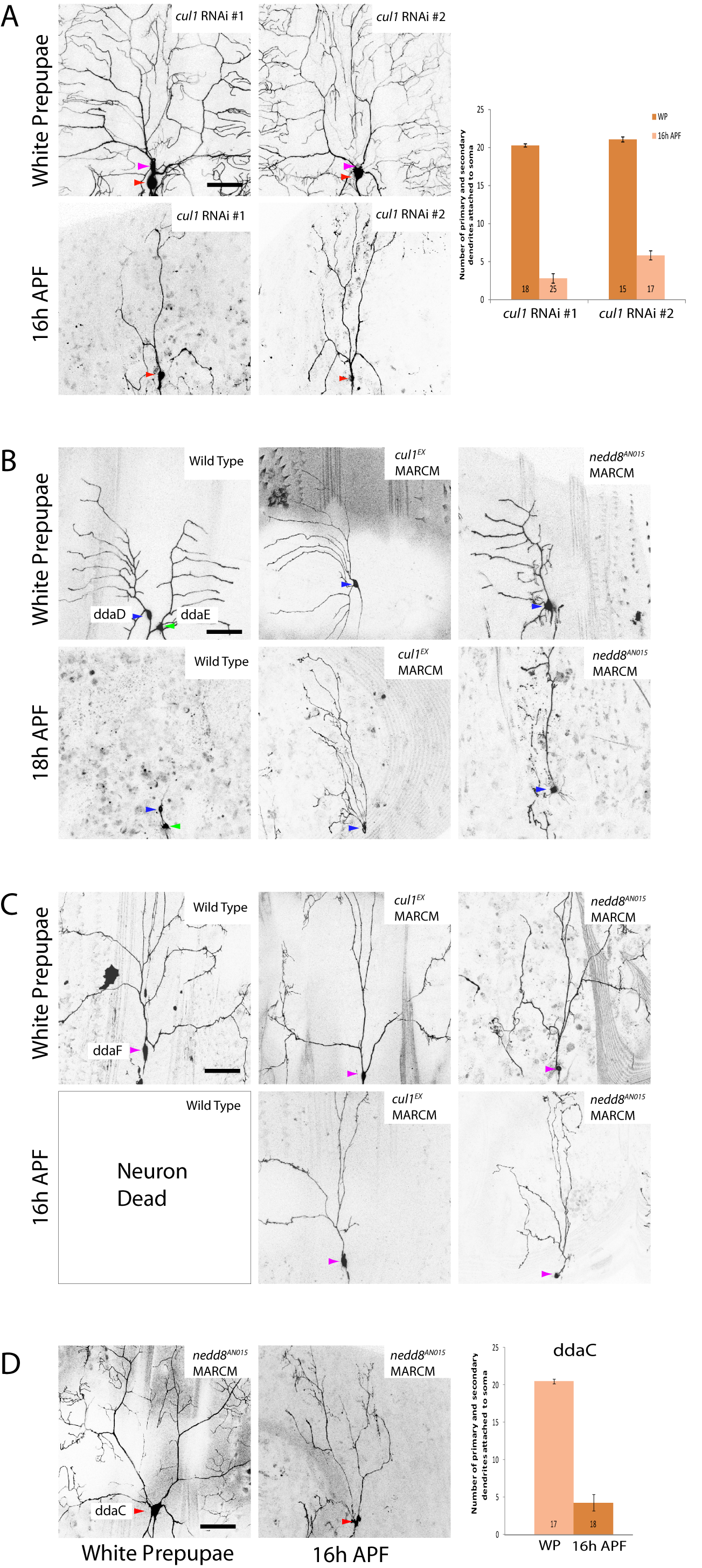

Supplement: Figure S1 — Cul1 and Nedd8 are required for remodeling of dda sensory neurons. (A–D) Live confocal images of dda neurons expressing UAS-mCD8-GFP at WP, 16 h, or 18 h APF. (A) ddaC neurons overexpressing one copy of cul1 RNAi #1 or cul1 RNAi #2 ddaC clones exhibited dendrite pruning defects at 16 h APF. Note: the severity of RNAi phenotype mainly depends on copy number/strength of ppk-Gal4 drivers as well as UAS-Dcr2. Expression level of ppk-Gal4 (inserted on Chr II) is much stronger than that of ppk-Gal4 (on Chr III). Thus, the phenotype of cul1 RNAi #2 in Figure 1C′ is almost double compared to that in Figure S1A due to the presence of two copies of ppk-Gal4 (Chr II). Please note that the ddaF neuron is occasionally labeled by one copy of ppk-Gal4 (Chr II) but always by two copies of the driver. (B) While wild-type class I ddaD/ddaE neurons pruned normally at 18 h APF, cul1EX, and nedd8AN015 MARCM ddaD neurons failed to prune their respective dendrites by 18 h APF. Blue arrowheads point to ddaD neurons, and green arrowheads to ddaE. (C) Wild-type class III ddaF neurons underwent apoptosis by 16 h APF, whereas cul1EX and nedd8AN015 MARCM ddaF neurons survived by 16 h APF. Purple arrowheads point to ddaF neurons. (D) Similar to cul1EX, nedd8AN015 MARCM ddaC neurons failed to prune their larval dendrites by 16 h APF. Quantification of the average number of primary and secondary dendrites attached to the soma of mutant ddaC neurons at WP and 16 h APF. Dorsal is up in all images. The number of samples (n) in each group is shown on the bars. Error bars represent S.E.M. The scale bars are 50 µm. See genotypes in Text S1. (TIF) [file pbio.1001657.s001.tif]

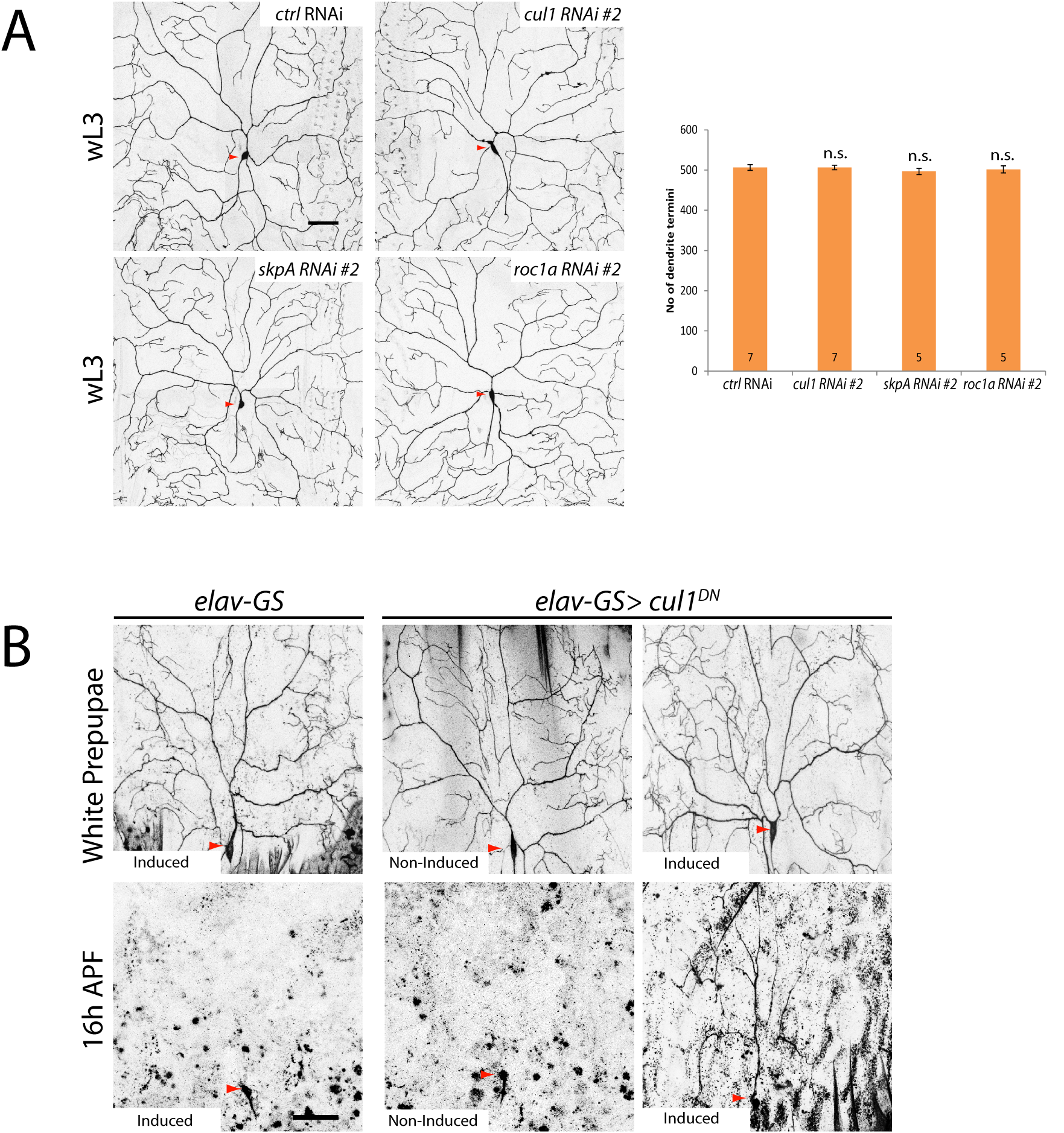

Supplement: Figure S2 — Initial dendrite development is not affected in cul1 RNAi, skpA RNAi, or roc1a RNAi ddaC neurons. (A) Live confocal images of ddaC neurons expressing UAS-mCD8-GFP at w3L. ddaC neurons overexpressing cul1 RNAi #2, skpA RNAi #2, or roc1a RNAi #2 via one copy of ppk-Gal4 (on Chr II) showed similar w3L dendrite morphology, compared to the wild-type control. Quantification of the average number of dendritic termini of each genotype. The number of samples (n) in each group is shown on the bars. Error bars represent S.E.M. n.s., not significant. (B) Live confocal images of ddaC neurons labeled by ppk-CD4-tdTomato. RU486 treatment did not affect dendrite pruning in the wild-type ddaC neurons (the far left panels). Using the RU486-inducible Gene-Switch system, inducible expression of cul1DN resulted in dendrite pruning defects in 16 h APF ddaC neurons, compared to 0% of the noninduced controls. WP dendrite morphology of induced versus noninduced ddaC neurons was similar. Please note that weak dendrite signals at 16 h APF are due to less prominent fluorescence of tdTomato. See genotypes in Text S1. (TIF) [file pbio.1001657.s002.tif]

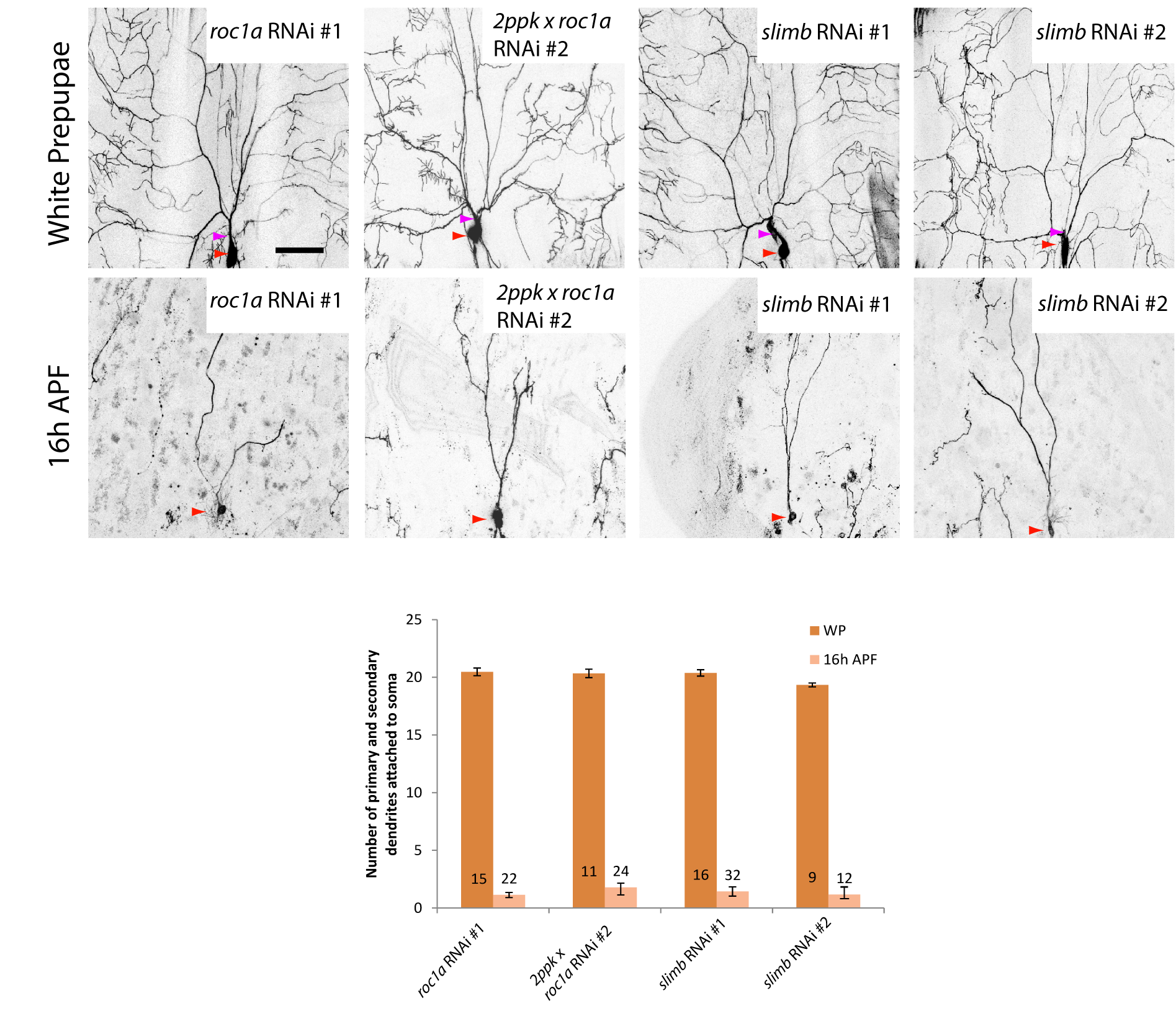

Supplement: Figure S3 — Roc1a, SkpA, and Slimb are required for pruning of class IV ddaC neurons. Live confocal images of ddaC neurons expressing UAS-mCD8-GFP at WP or 16 h APF. ddaC neurons expressing one copy of Roc1a RNAi #1, two copies of Roc1a RNAi #2, one copy of slimb RNAi #1, or one copy of slimb RNAi #2 exhibited prominent pruning defects. Quantification of the average number of primary and secondary dendrites attached to the soma of mutant ddaC neurons at WP and 16 h APF. Dorsal is up in all images. The number of samples (n) in each group is shown on the bars. Error bars represent S.E.M. The scale bars are 50 µm. See genotypes in Text S1. (TIF) [file pbio.1001657.s003.tif]

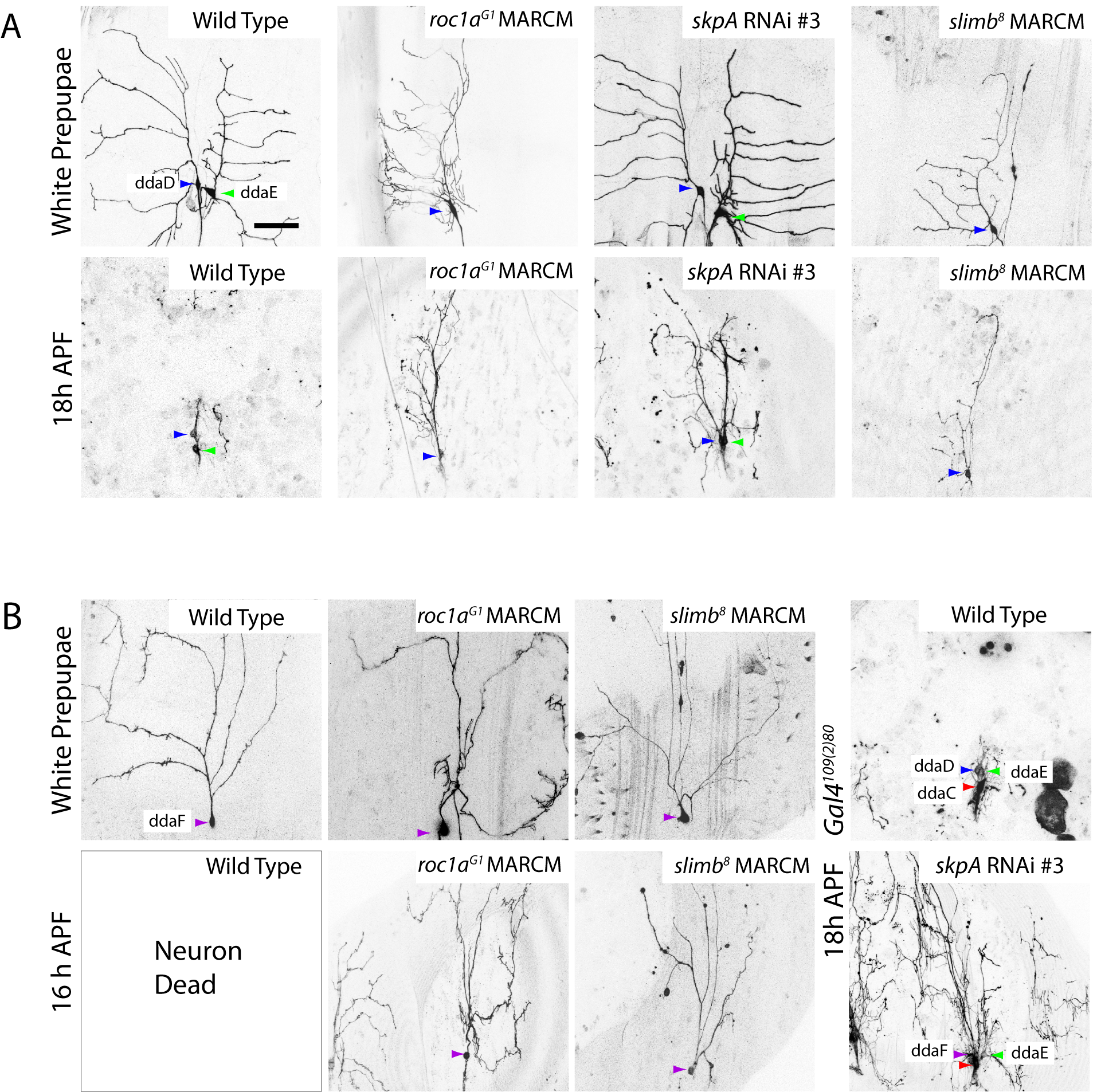

Supplement: Figure S4 — Roc1a, SkpA, and Slimb are required for pruning of class I ddaD/E neurons and apoptosis of class III ddaF neurons. (A and B) Live confocal images of dda neurons expressing UAS-mCD8-GFP at WP, 16 h, or 18 h APF. (A) Wild-type class I ddaD/ddaE neurons pruned normally at 18 h APF, whereas roc1aG1 MARCM, Slimb8 MARCM, and SkpA RNAi expressing ddaD neurons failed to prune their larval dendrites by 18 h APF. Blue arrowheads point to ddaD neurons, and green arrowheads to ddaE. (B) Wild-type class III ddaF neurons underwent apoptosis by 16 h APF, whereas roc1aG1 and slimb8 MARCM ddaF neurons survived at 16 h APF. Knockdown of SkpA in ddaF neurons via the da neuronal driver 109(2)80-Gal4 driven skpA RNAi expression also resulted in failure of ddaF to undergo apoptosis at 18 h APF. Purple arrowheads point to ddaF neurons. Dorsal is up in all images. See genotypes in Text S1. (TIF) [file pbio.1001657.s004.tif]

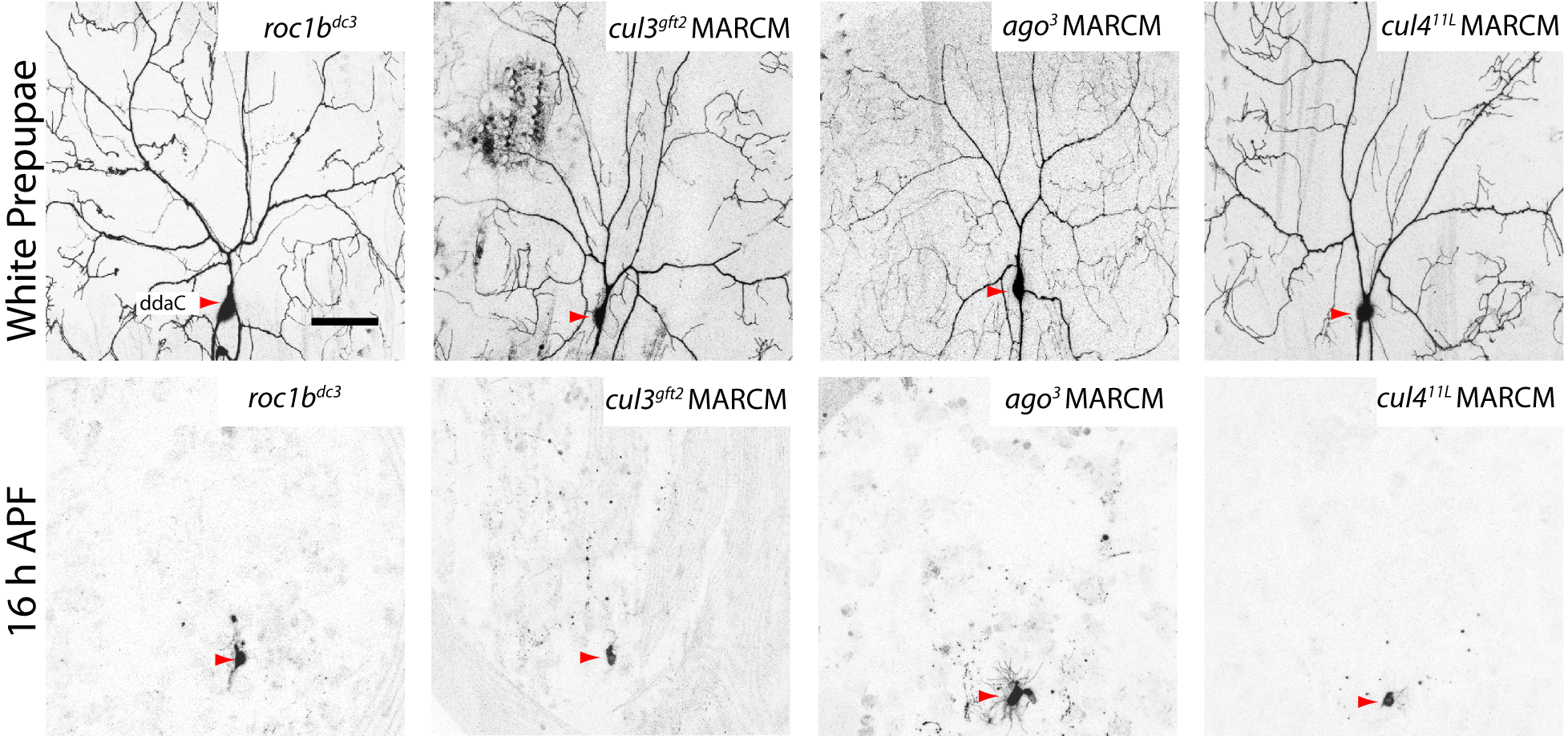

Supplement: Figure S5 — Roc1b, Cul3, Ago, and Cul4 are not required for pruning of class IV ddaC neurons. Similar to wild-type ddaC neurons, roc1bdc3 homozygous mutant, cul3gft2, ago3, or cul411L MARCM ddaC neurons pruned their dendrites normally at 16 h APF. Dorsal is up in all images. See genotypes in Text S1. (TIF) [file pbio.1001657.s005.tif]

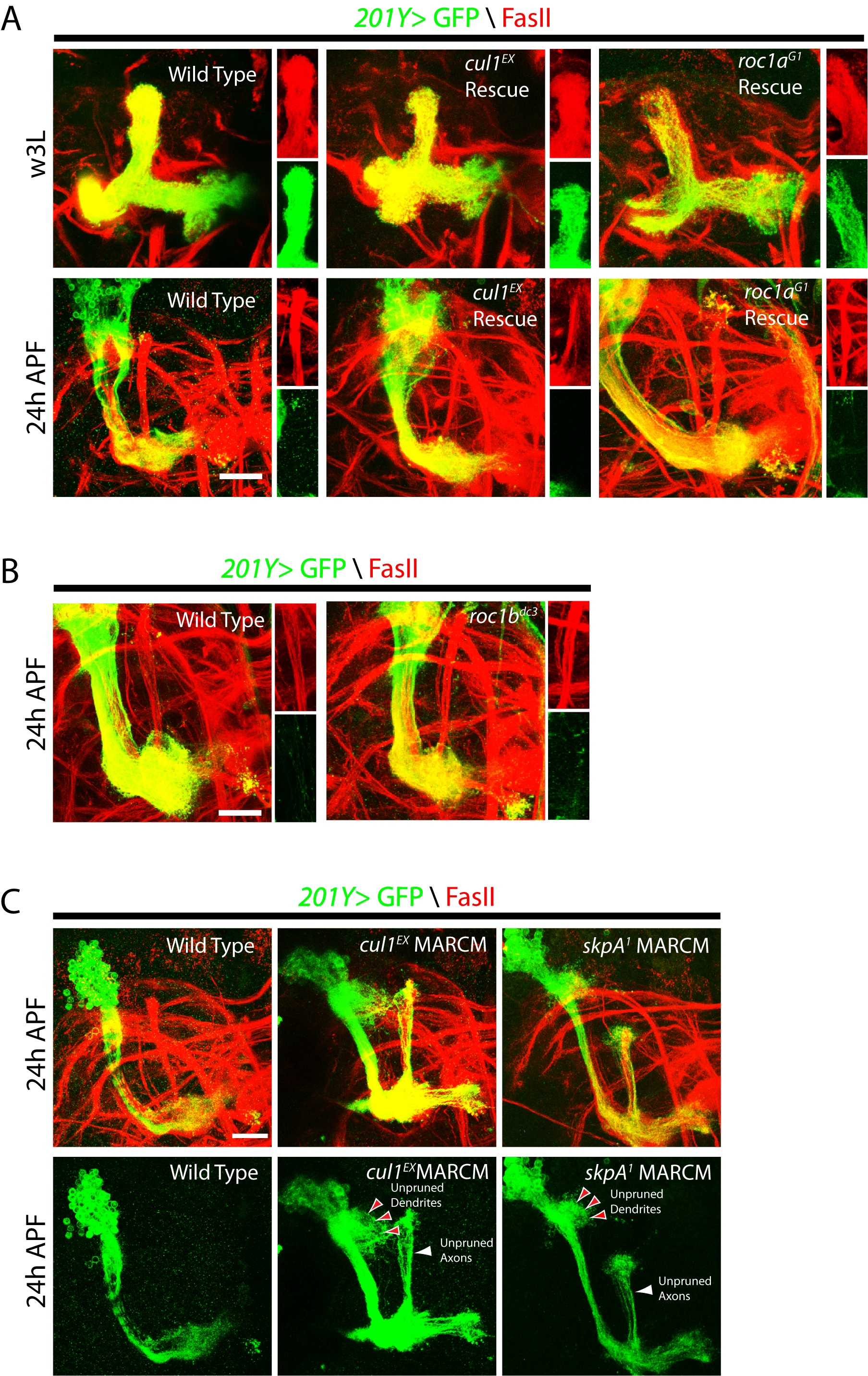

Supplement: Figure S6 — The Cul1-based SCF E3 ligase is required for MB γ neuron remodelling. (A–C) Confocal images of MB γ neurons expressing UAS-mCD8-GFP driven by 201Y-Gal4 at wL3 or 24 h APF in wild-type, cul1EX MB neuroblast clones expressing the full-length Cul1, roc1aG1 MB neuroblast clone expressing the full-length Roc1a, and roc1bdc3 MB neuroblast mutants. (A) Overexpression of Cul1 in cul1EX MB or Roc1a in roc1aG1 MB neuroblast clones fully rescued their axon pruning defects. (B) Roc1b is not required for MB axon pruning. (C) culEX and skpA1 MB neuroblast clones failed to prune their dendrites at 24 h APF compared to the wild-type control. Red arrowheads point to the unpruned dendrites. The scale bars are 50 µm. See genotypes in Text S1. (TIF) [file pbio.1001657.s006.tif]

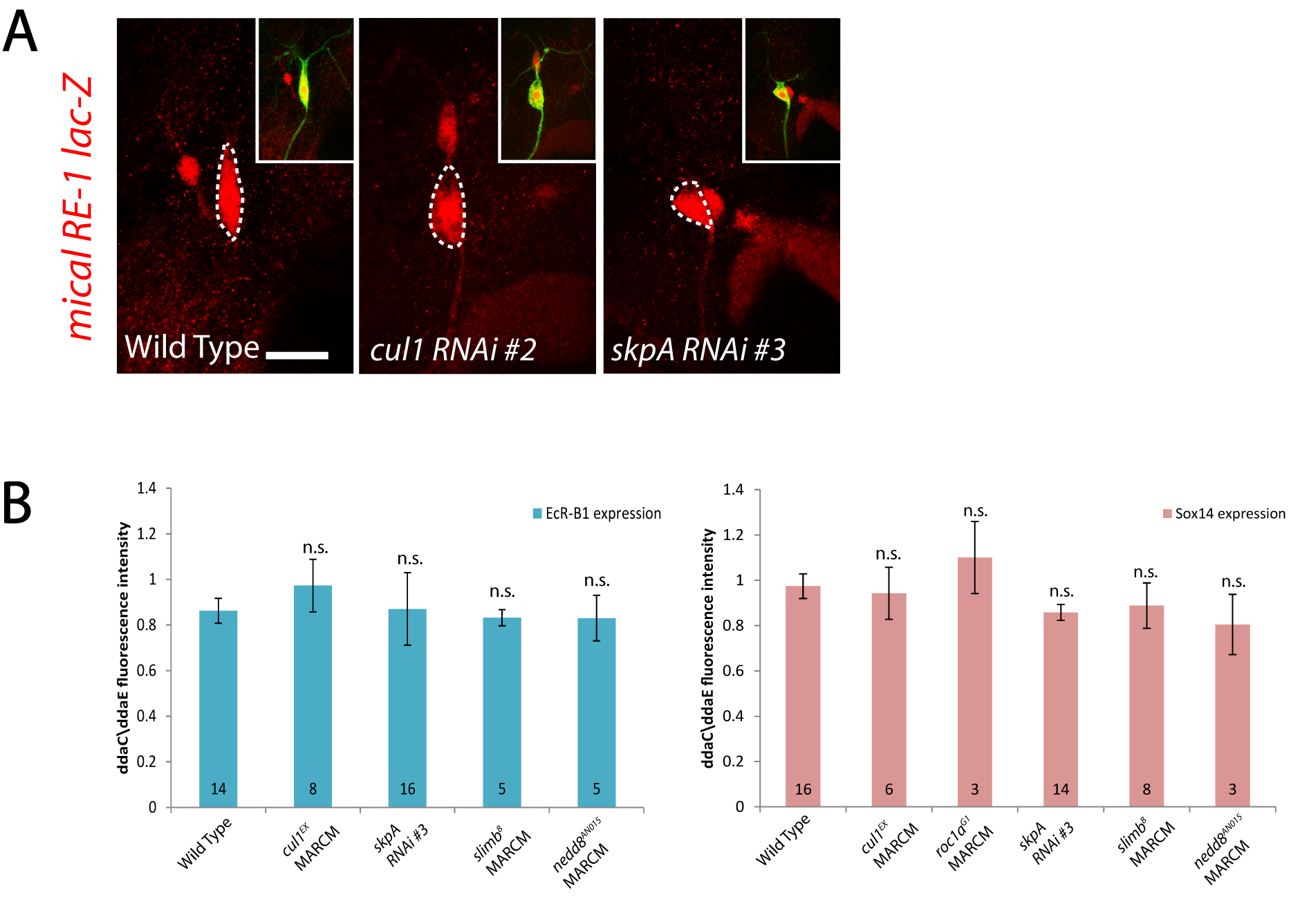

Supplement: Figure S7 — The Cul1-based SCF E3 ligase is not required for EcR-B1/sox14 expression or mical transcription. (A) Confocal images of ddaC neurons expressing UAS-mCD8-GFP driven by ppk-Gal4 at WP immunostained for LacZ (shown in red). The mical-lacZ reporter drives upregulation of LacZ expression at WP under a mical enhancer. mical transcription was not affected in cul1 RNAi or skpA RNAi ddaC neurons at WP. (B) Quantification of immunostaining for EcR-B1 and Sox14. The graphs display the average values of ddaC/ddaE ratios and S.E.M. n is shown on the bars. EcR-B1 and Sox14 expression in wild-type, cul1EX MARCM, roc1aG1 MARCM, skpA RNAi, slimb8 MARCM, or nedd8AN015 MARCM ddaC neurons remained largely unchanged. Dorsal is up in all images. n.s., not significant. The scale bar in (A) is 20 µm. See genotypes in Text S1. (TIF) [file pbio.1001657.s007.tif]

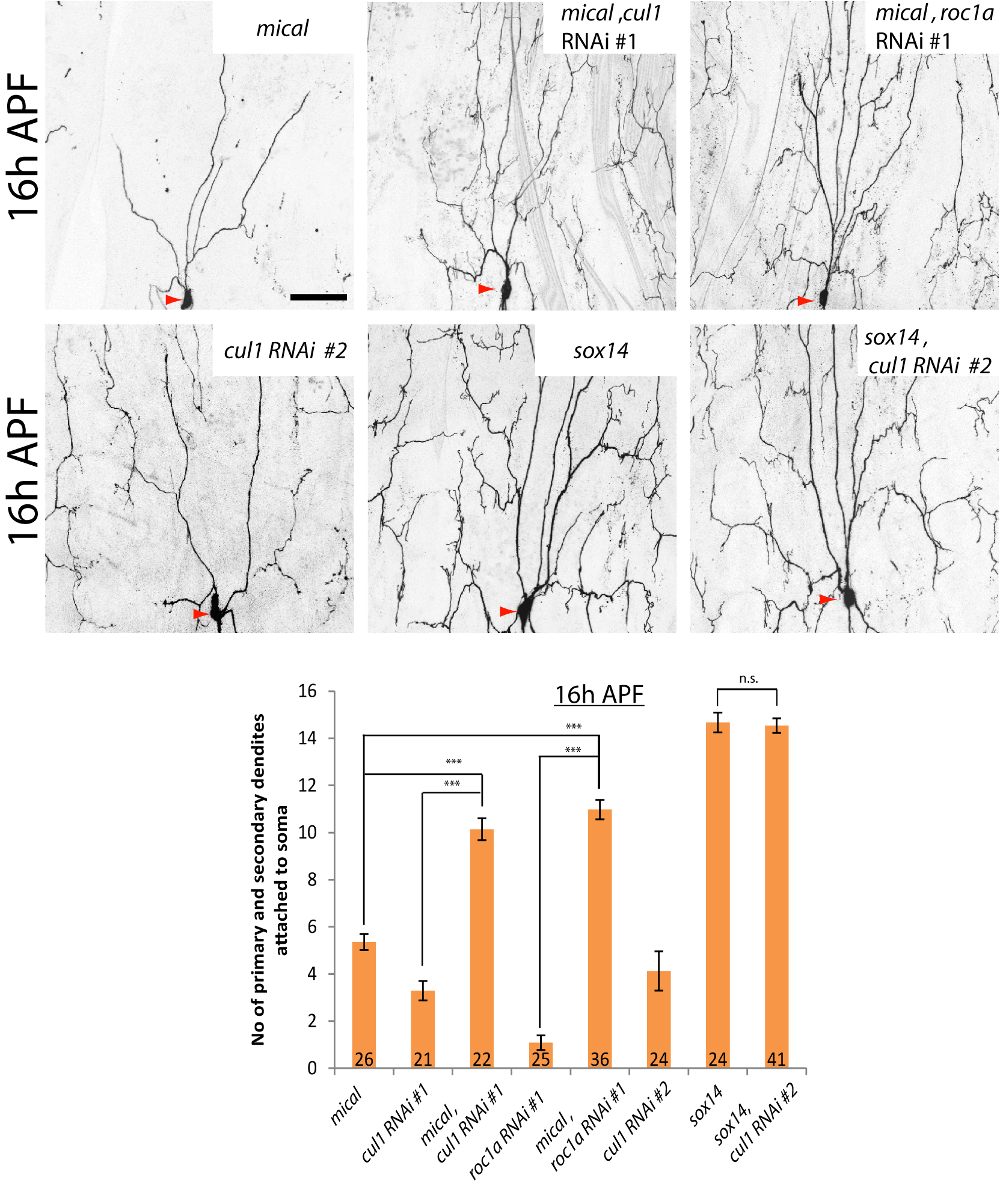

Supplement: Figure S8 — The Cul1-based SCF E3 ligase acts downstream of Sox14 but in parallel to Mical to mediate dendrite pruning. mical ddaC neurons displayed a pruning defect with the average of 5.3 primary and secondary dendrites attached to the soma at 16 h APF. Knockdown of cul1 and roc1a in the mical background with their respective RNAi lines significantly enhanced the mical pruning defects. Knockdown of cul1 with cul1 RNAi #2 in ddaC neurons displayed moderate pruning defect with the average of 4.1 primary and secondary dendrites attached to the soma. sox14 ddaC neurons displayed a severe pruning defect with the average of 14.7 primary and secondary dendrites attached to the soma at 16 h APF . Knockdown of cul1 in sox14 mutant resulted in no enhancement of pruning defect, compared to sox14 mutant alone. Quantification of the average number of primary and secondary dendrites attached to the soma of mutant ddaC neurons at WP. The number of samples (n) in each group is shown on the bars. Error bars represent S.E.M. Dorsal is up in all images. ***p<0.001. n.s., not significant. Scale bar is 20 µm. See genotypes in Text S1. (TIF) [file pbio.1001657.s008.tif]

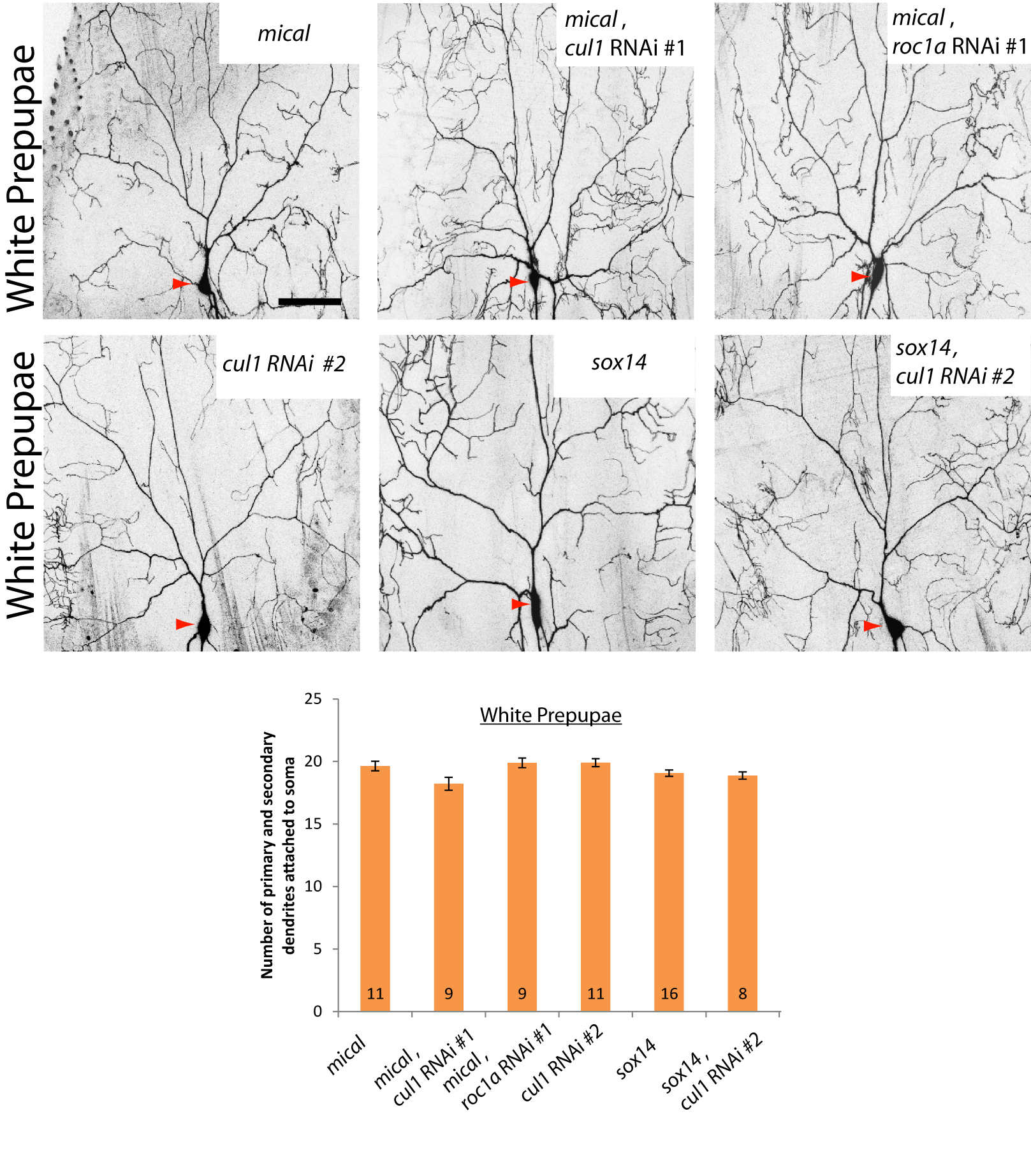

Supplement: Figure S9 — ddaC neurons of various double mutant combinations have a similar number of major dendrites attached to their somas at WP stage. Live confocal images of ddaC neurons expressing UAS-mCD8-GFP driven by ppk-Gal4 at WP. Knockdown of Cul1 or Roc1a in the mical background did not significantly alter the ddaC WP morphology. sox14 mutant, cul1 RNAi, cul1 RNAi, and sox14 double mutant ddaC neurons displayed similar elaboration of primary and secondary dendrites at WP stage. Quantification of the average number of primary and secondary dendrites attached to the soma of mutant ddaC neurons at WP. The number of samples (n) in each group is shown on the bars. Error bars represent S.E.M. Dorsal is up in all images. Scale bar is 20 µm. See genotypes in Text S1. (TIF) [file pbio.1001657.s009.tif]

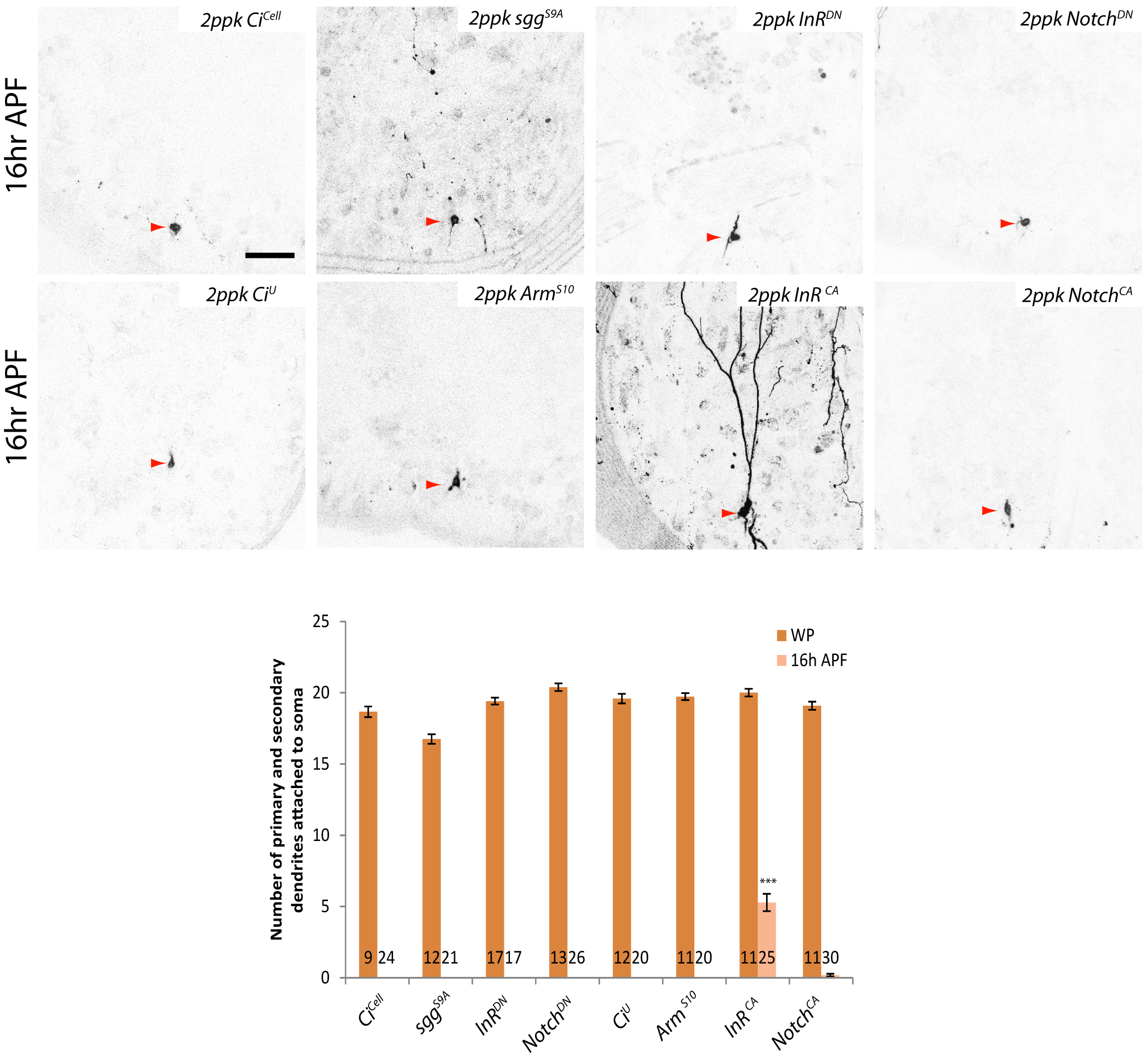

Supplement: Figure S10 — Activation of the Insulin pathway in ddaC neurons results in ddaC dendrite pruning defects. Live confocal images of ddaC neurons expressing UAS-mCD8-GFP driven by ppk-Gal4 at 16 h APF. Inactivation of Hh, Wg, Insulin, or Notch pathways via expression of their respective repressors in ddaC neurons did not result in any pruning defects. Activation of the Insulin signaling via InRCA, but not activation of Hh, Wg, or Notch signalling in ddaC neurons, led to a notable pruning defect at 16 h APF. Quantification of the average number of primary and secondary dendrites attached to the soma of mutant ddaC neurons at WP and 16 h APF. The number of samples (n) in each group is shown on the bars. Error bars represent S.E.M. Dorsal is up in all images. ***p<0.001. Scale bar is 20 µm. See genotypes in Text S1. (TIF) [file pbio.1001657.s010.tif]

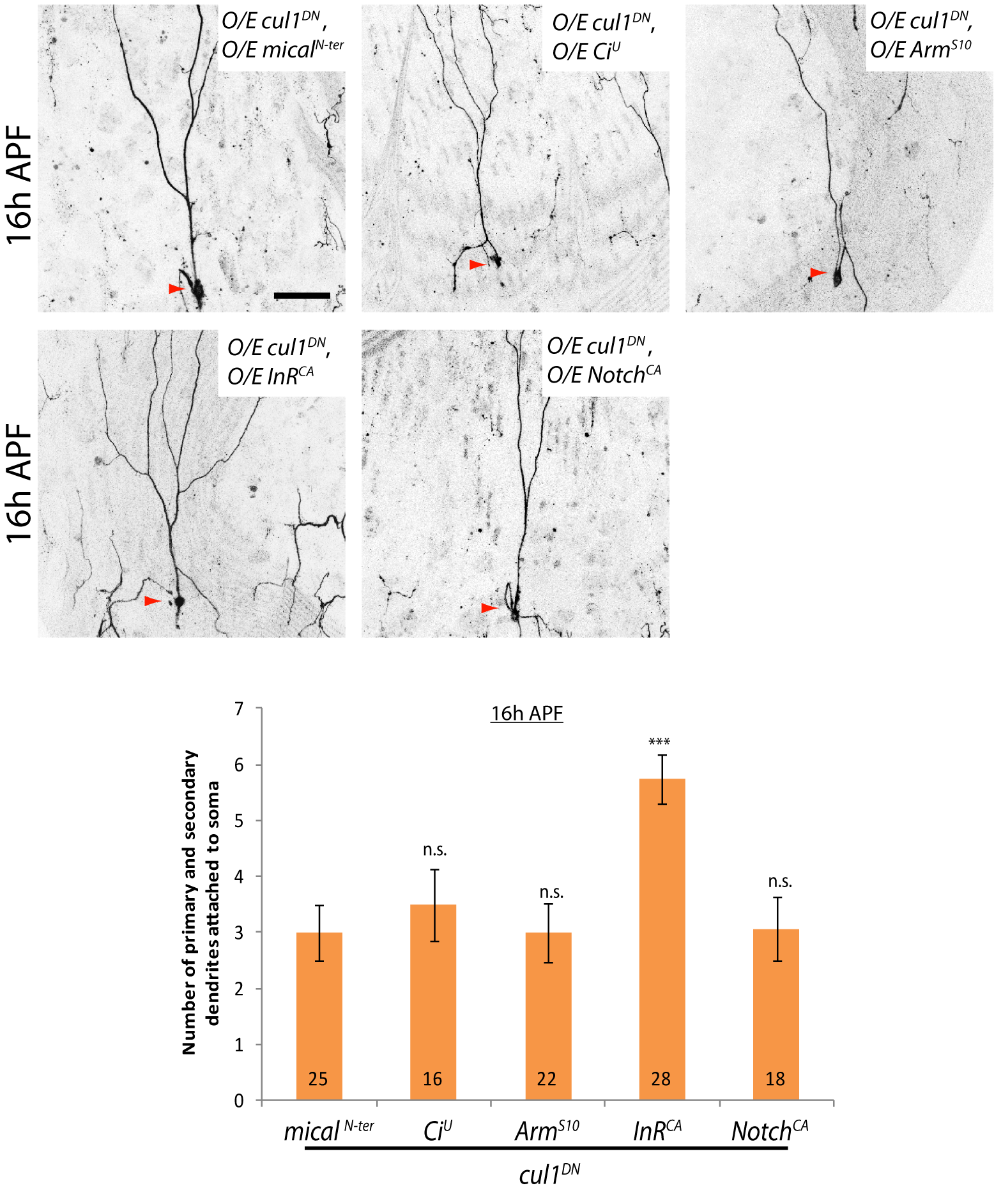

Supplement: Figure S11 — Activation of the Insulin pathway in ddaC neurons enhances dendrite pruning defects in cul1DN expressing ddaC neurons. Co-expression of InRCA, but not other activators of various pathways examined, significantly enhanced cul1DN-mediated pruning defects in ddaC neurons. Quantification of the average number of primary and secondary dendrites attached to the soma of mutant ddaC neurons at 16 h APF. The number of samples (n) in each group is shown on the bars. Error bars represent S.E.M. Dorsal is up in all images. ***p<0.001. n.s., not significant. Scale bar is 20 µm. See genotypes in Text S1. (TIF) [file pbio.1001657.s011.tif]

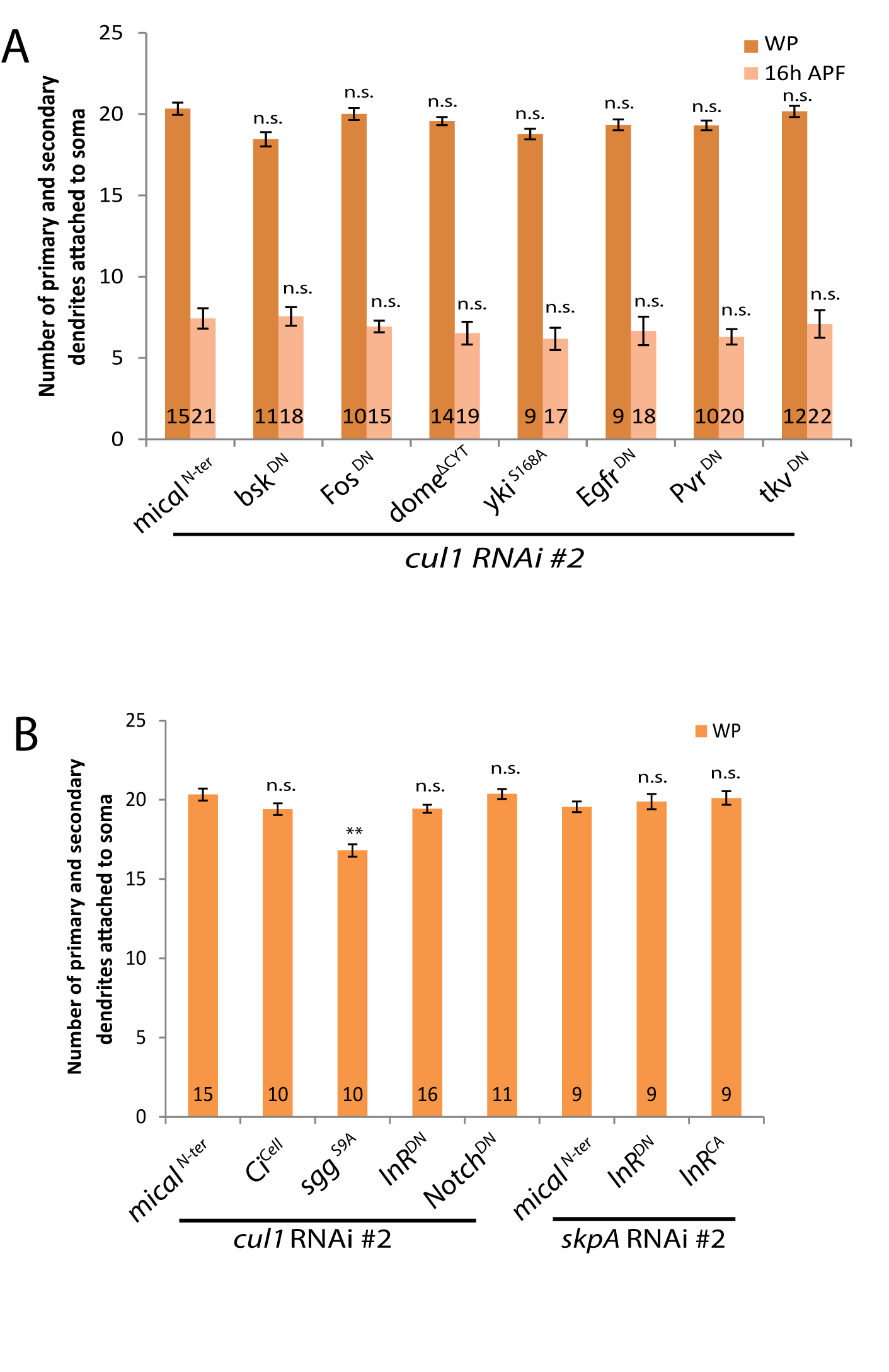

Supplement: Figure S12 — Attenuation of other signaling pathways is unable to rescue cul1 RNAi-mediated dendrite pruning defect. (A) Attenuation of JNK, JAK/STAT, Hippo, EGFR, PVR, and Dpp pathways in ddaC neurons by co-expression of BskDN (JNK), FosDN (JNK), DomeΔCYT (JAK/STAT), YkiS168A (Hippo), EgfrDN (EGFR), PvrDN (PVR), or TkVDN (Dpp) with cul1 RNAi was unable to rescue cul1 RNAi-mediated dendrite pruning defects, similar to the micalN-ter control. Quantification of the average number of primary and secondary dendrites attached to the soma of ddaC neurons at WP and 16 h APF. (B) Quantification of the average number of WP primary and secondary dendrites attached to various genotypes of ddaC neurons in Figure 6A–H. The number of samples (n) in each group is shown on the bars. ** p<0.01. n.s., not significant. Error bars represent S.E.M. See genotypes in Text S1. (TIF) [file pbio.1001657.s012.tif]

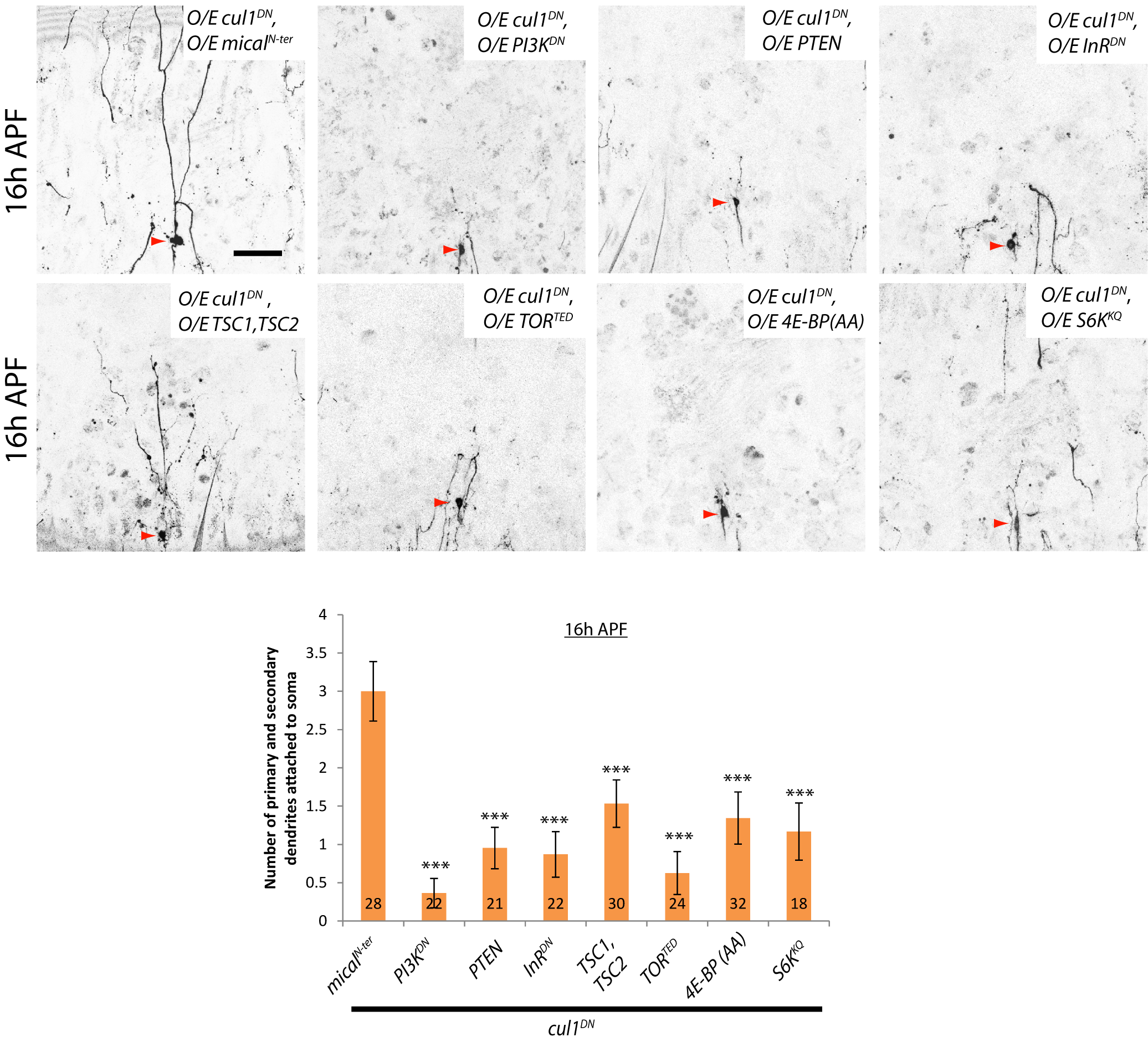

Supplement: Figure S13 — Attenuation of PI3K/TOR signaling pathway is sufficient to rescue cul1DN -mediated ddaC dendrite pruning defect. Inhibition of the PI3K/TOR pathway was also able to suppress cul1DN pruning defects. While ddaC neurons co-expressing nonfunctional micalN-ter with cul1DN displayed apparent pruning defects, co-expression of PI3KDN, PTEN, InRDN, TSC1/TSC2, TORTED, 4E-BP(AA), or S6KKQ significantly suppressed cul1DN pruning defects. Quantification of the average number of primary and secondary dendrites attached to the soma of mutant ddaC neurons at 16 h APF. The number of samples (n) in each group is shown on the bars. Error bars represent S.E.M. Dorsal is up in all images. ***p<0.001. Scale bar is 20 µm. See genotypes in Text S1. (TIF) [file pbio.1001657.s013.tif]

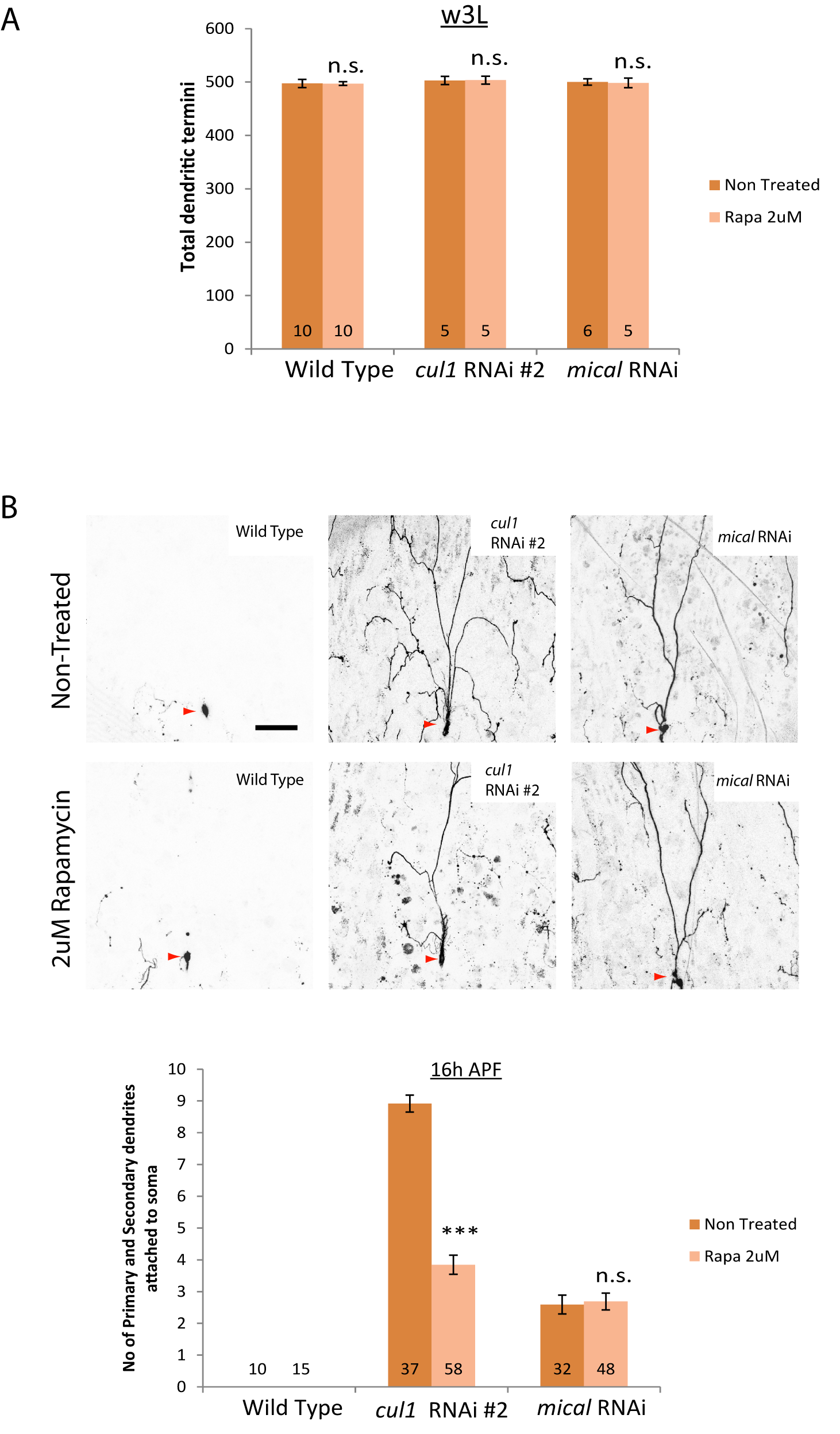

Supplement: Figure S14 — Pharmacological attenuation of the InR/PI3K/TOR signaling significantly suppresses the dendrite pruning defects in cul1 RNAi ddaC neurons. (A) Rapamycin treatment did not affect initial dendrite development in cul1 RNAi or mical RNAi-expressing ddaC neurons. Quantifications of the total dendritic termini of Rapamycin-treated or nontreated mutant ddaC neurons at w3L. (B) Rapamycin treatment, similar to the effects of InRDN and PI3KDN, significantly suppressed the dendrite pruning defects in cul1 RNAi ddaC neurons, but not in mical RNAi ddaC neurons. Quantification of the average number of primary and secondary dendrites attached to the soma of mutant ddaC neurons at 16 h APF. The number of samples (n) in each group is shown on the bars. ***p<0.001. n.s., not significant. Error bars represent S.E.M. See genotypes in Text S1. (TIF) [file pbio.1001657.s014.tif]

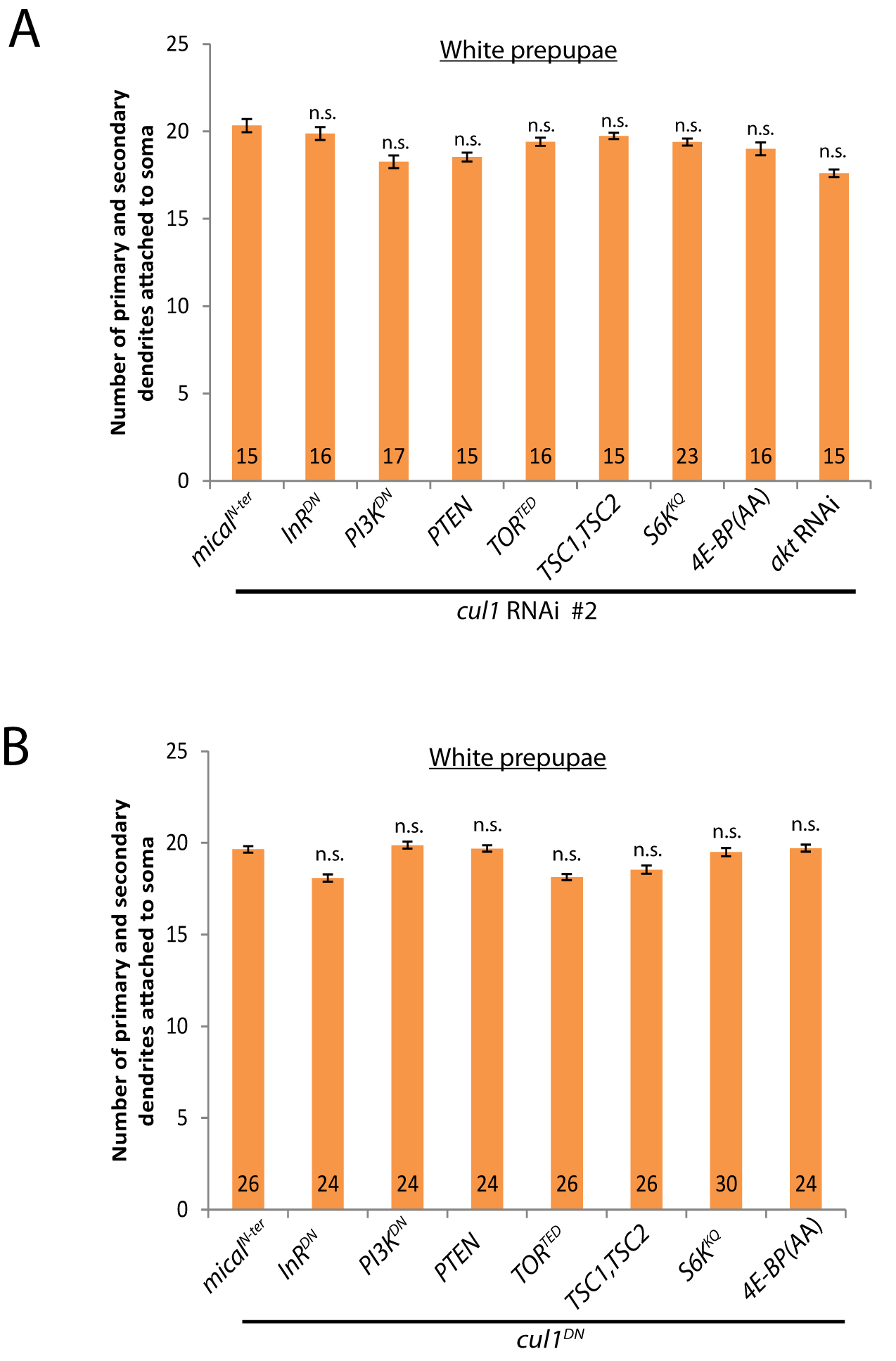

Supplement: Figure S15 — Attenuation of PI3K/TOR signaling does not alter the number of major dendrites attached to ddaC somas at WP stage. (A) Co-expression of nonfunctional micalN-ter, InRDN, PI3KDN, PTEN, TORTED, TSC1/TSC2, S6KKQ, 4E-BP(AA), or akt RNAi with cul1 RNAi resulted in normal elaboration of primary and secondary dendrites in ddaC neurons at the WP stage. (B) Co-expression of nonfunctional micalN-ter, InRDN, PI3KDN, PTEN, TORTED, TSC1/TSC2, S6KKQ, and 4E-BP(AA) with cul1DN resulted in normal elaboration of primary and secondary dendrites in ddaC neurons at the WP stage. Quantification of the average number of primary and secondary dendrites attached to the soma of mutant ddaC neurons at 16 h APF. The number of samples (n) in each group is shown on the bars. n.s., not significant. Error bars represent S.E.M. See genotypes in Text S1. (TIF) [file pbio.1001657.s015.tif]

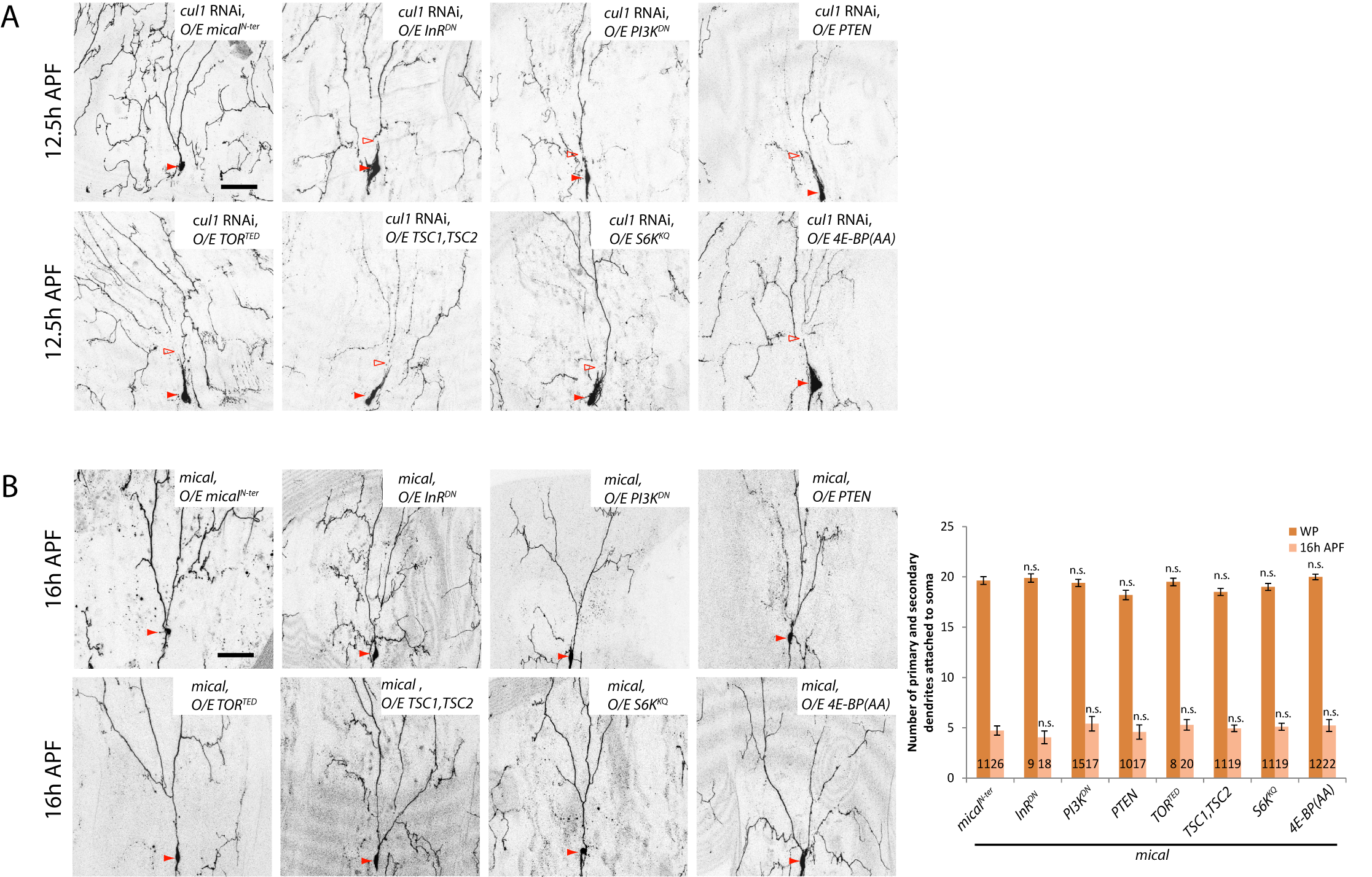

Supplement: Figure S16 — Specific effects of the InR/PI3K/TOR pathway on ddaC dendrite pruning. (A and B) Live confocal images of ddaC neurons expressing UAS-mCD8-GFP driven by ppk-Gal4 at 12.5 h or 16 h APF. (A) While all ddaC neurons co-expressing nonfunctional micalN-ter with cul1 RNAi failed to sever the proximal regions of their dorsal dendrite branch at 12.5 h APF, proximal severing of dorsal dendrite branches was observed in cul1 RNAi ddaC neurons co-expressing InRDN, PI3KDN, PTEN, TORTED, TSC1/TSC2, S6KKQ, or 4E-BP(AA). Empty red arrowheads point to proximal severing of the dorsal dendrite branches. (B) The expression of nonfunctional micalN-ter control, InRDN, PI3KDN, PTEN, TORTED, TSC1/TSC2, S6KKQ, or 4E-BP(AA) was unable to rescue the dendrite pruning defects in mical mutant ddaC neurons. Quantification of the average number of primary and secondary dendrites attached to the soma of mutant ddaC neurons at WP and 16 h APF. The number of samples (n) in each group is shown on the bars. Error bars represent S.E.M. n.s., not significant. Dorsal is up in all images. The scale bar is 50 µm. See genotypes in Text S1. (TIF) [file pbio.1001657.s016.tif]

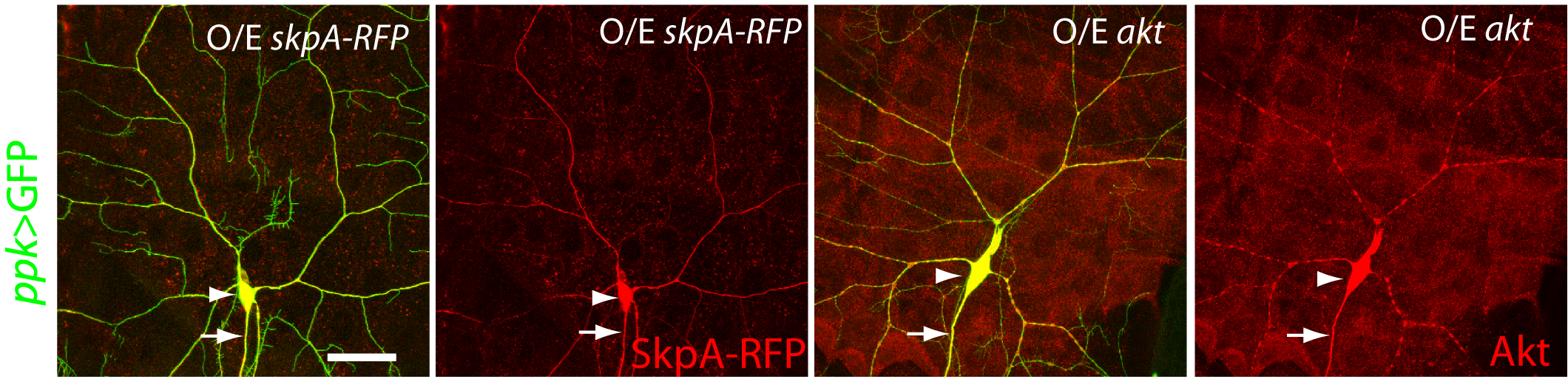

Supplement: Figure S17 — Overexpressed SkpA-RFP and Akt are localized uniformly throughout the ddaC neurons. SkpA-RFP and Akt were labeled in red, and mCD8-GFP in green. White arrowheads and arrows point to ddaC somas and axons, respectively. The scale bars are 50 µm. Dorsal is up in all images. See genotypes in Text S1. (TIF) [file pbio.1001657.s017.tif]

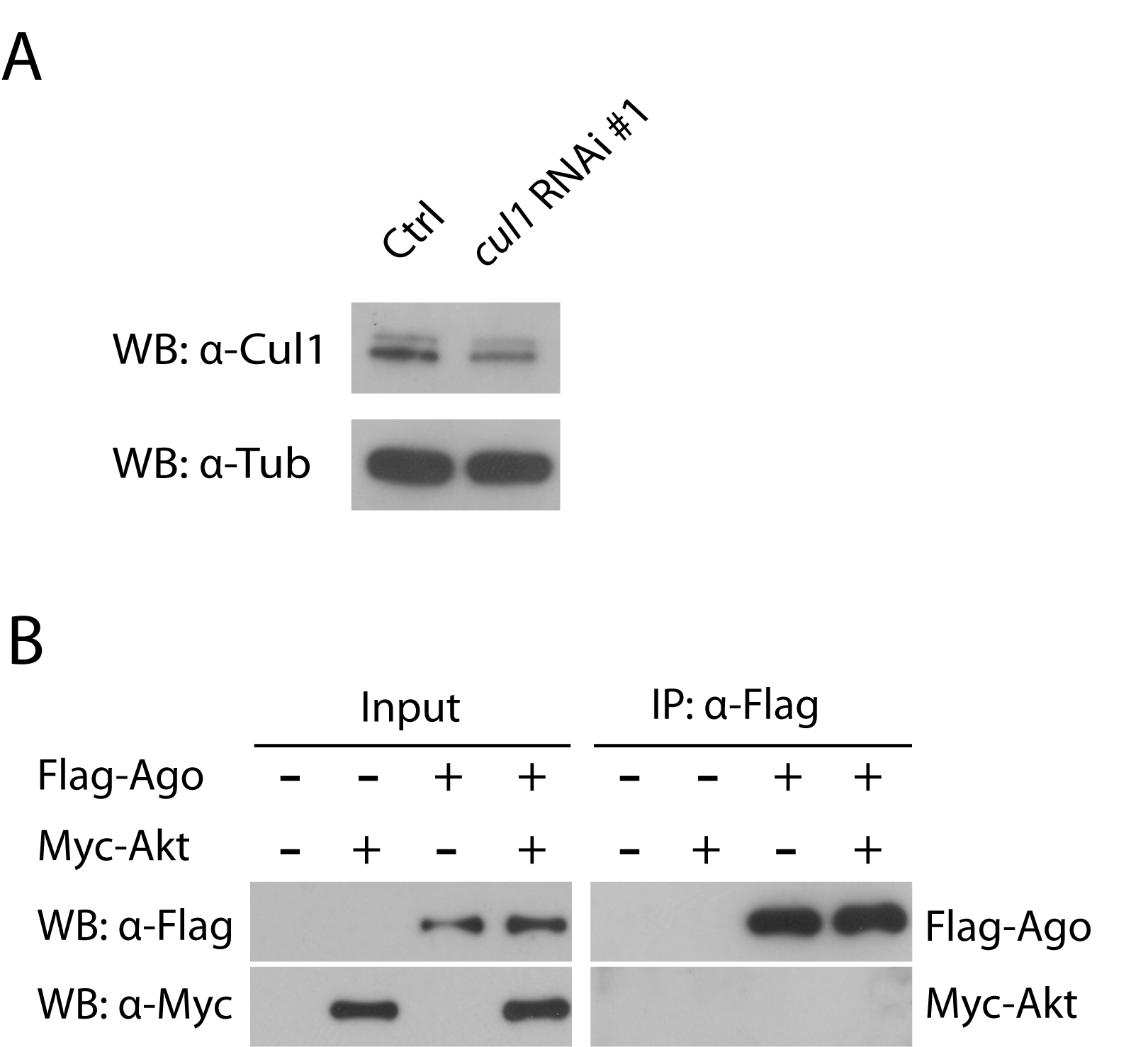

Supplement: Figure S18 — Specificity of cul1 knockdown in the brain. Akt is unable to associate with another F-box containing protein, Ago. (A) Cul1 protein levels were reduced via cul1 RNAi lines #1 using a pan-neuronal driver elav-Gal4. See genotypes in Text S1. (B) Akt did not associate with another F-box-containing protein Ago in S2 cells cotransfected with Flag-Ago and Myc-Akt. (TIF) [file pbio.1001657.s018.tif]

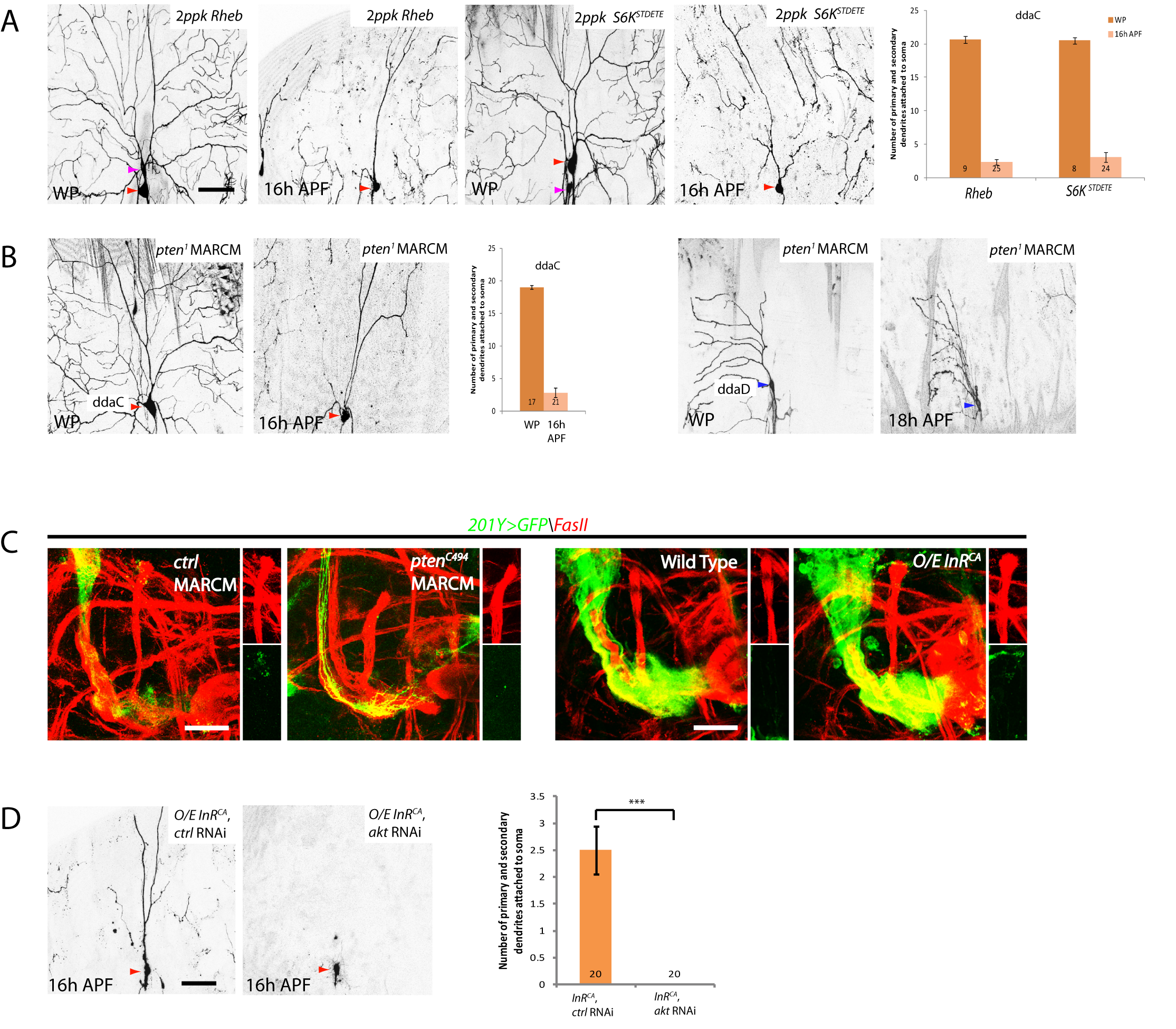

Supplement: Figure S19 — Activation of the InR/PI3K/TOR pathway is sufficient to inhibit ddaC dendrite pruning but not MB γ axon pruning. (A, B, and D) Live confocal images of dda neurons expressing UAS-mCD8-GFP driven at WP, 16 h APF, or 18 h APF. (A) Activation of the InR/PI3K/TOR pathway by the expression of Rheb or S6KSTDETE in ddaC neurons resulted in ddaC dendrite pruning defects. (B) pten1 MARCM ddaC neurons exhibited dendrite pruning defects at 16 h APF; similarly, its ddaD neurons also failed to prune at 18 h APF. Quantification of the average number of primary and secondary dendrites attached to the soma of mutant ddaC neurons at WP or 16 h APF. The number of samples (n) in each group is shown on the bars. Error bars represent S.E.M. Dorsal is up in all images. (C) Confocal images of MB γ neurons expressing UAS-mCD8-GFP driven by 201Y-Gal4 at 24 h APF. Similar to wild-type MB γ neurons, ptenc494 MB γ neurons MARCM clones and InRCA-expressing MB γ neurons pruned their dorsal and medial axon branches by 24 h APF. (D) Expression of akt RNAi in InRCA-expressing ddaC neurons fully suppressed InRCA-mediated dendrite pruning defect. The scale bars are 50 µm. See genotypes in Text S1. (TIF) [file pbio.1001657.s019.tif]

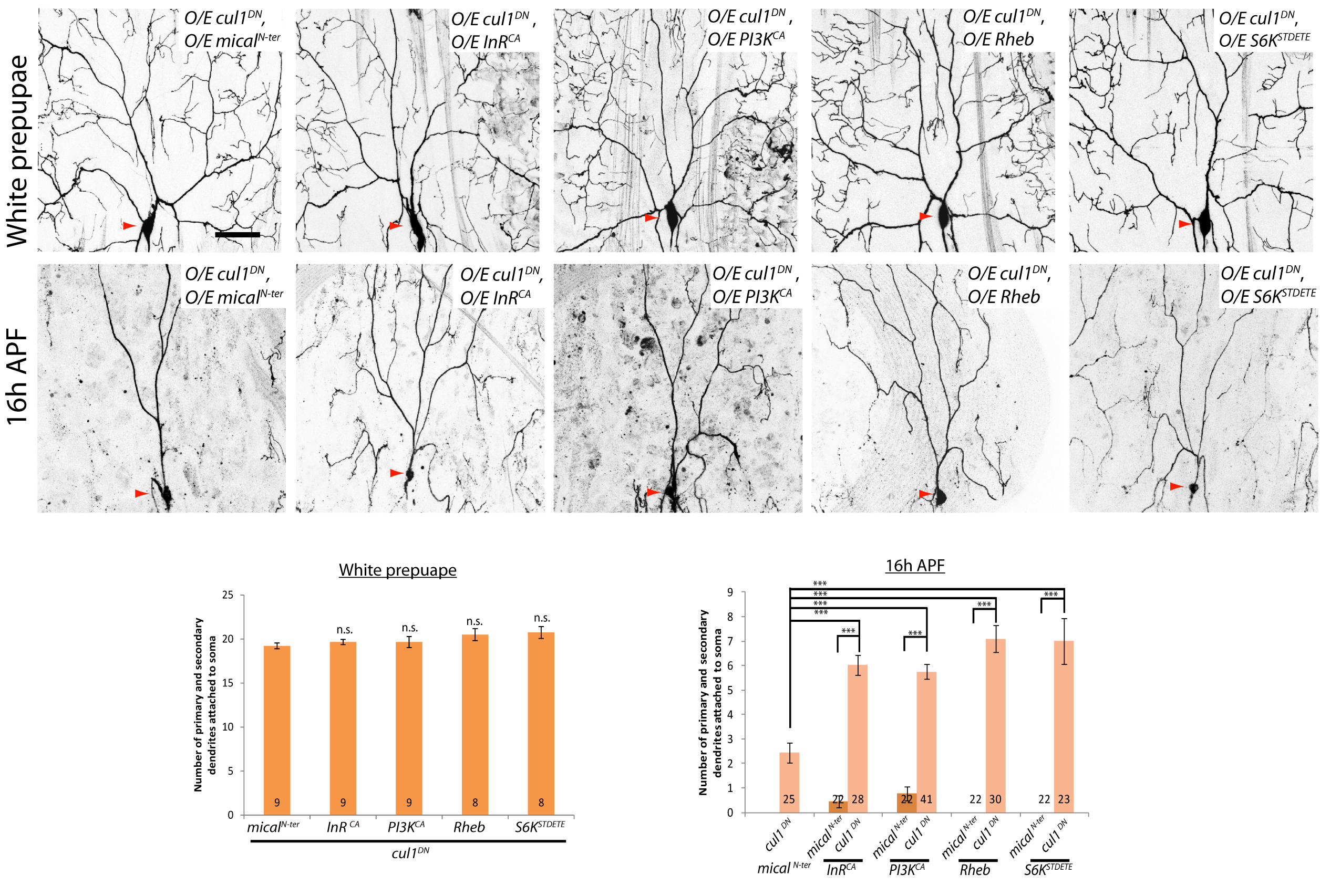

Supplement: Figure S20 — Activation of the InR/PI3K/TOR pathway enhances cul1DN -mediated dendrite pruning defect. Activation of the InR/PI3K/TOR pathway by InRCA, PI3KCA, Rheb, or S6KSTDETE in cul1DN ddaC neurons did not affect normal dendrite arborization at WP. Co-expression of InRCA, PI3KCA, Rheb, or S6KSTDETE with cul1DN dramatically enhanced the pruning defects, compared to that of nonfunctional micalN-ter control. Quantification of the average number of primary and secondary dendrites attached to the soma of mutant ddaC neurons at WP or 16 h APF. The number of samples (n) in each group is shown on the bars. Error bars represent S.E.M. ***p<0.001. n.s., not significant. Dorsal is up in all images. The scale bar is 50 µm. See genotypes in Text S1. (TIF) [file pbio.1001657.s020.tif]

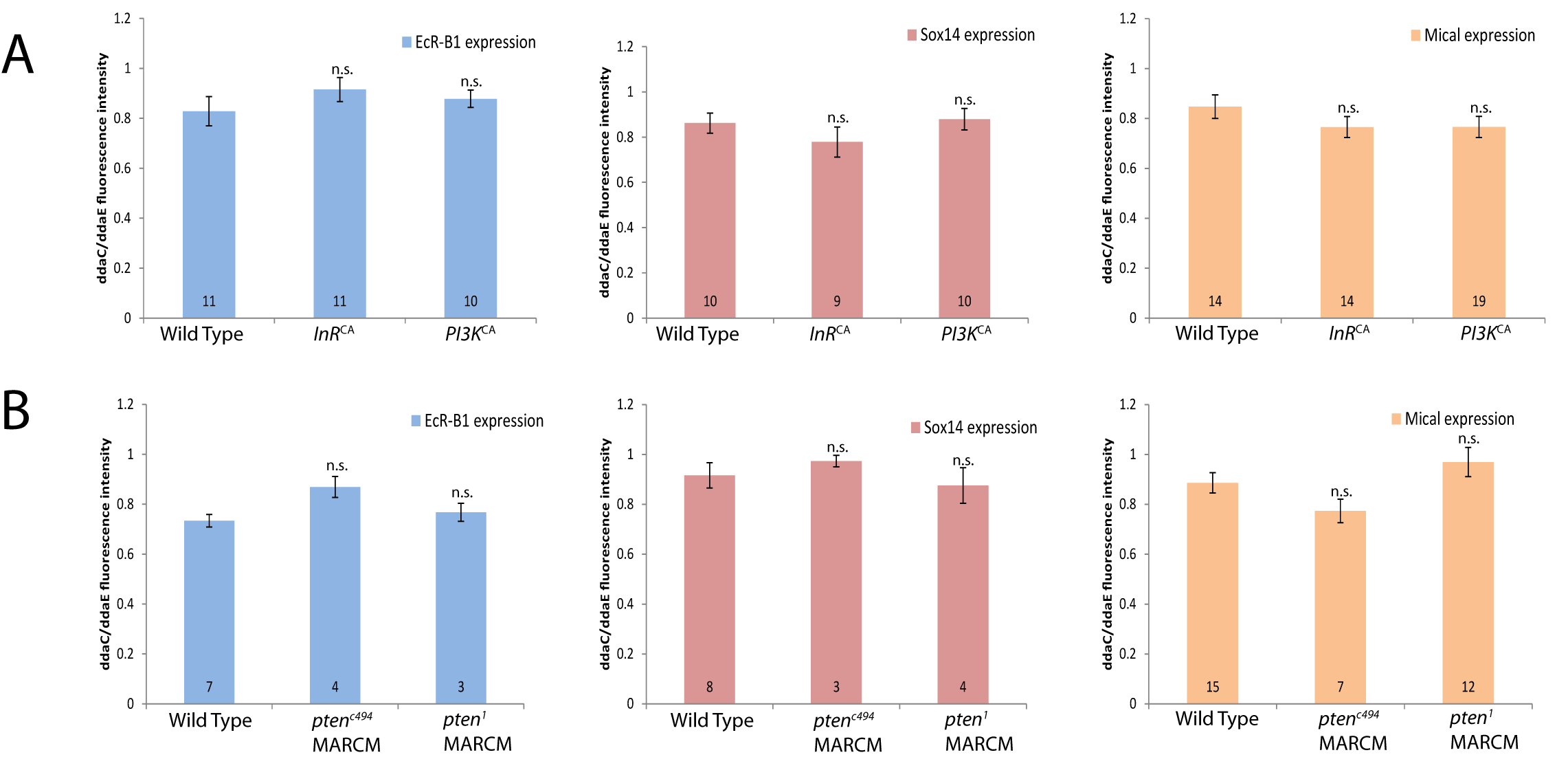

Supplement: Figure S21 — Activation of InR/PI3K/TOR pathway does not affect EcR-B1, Sox14, and Mical expression. (A and B) Activation of the InR/PI3K/TOR pathway via InRCA, PI3KCA, ptenc494 MARCM, or pten1 MARCM in ddaC neurons did not affect EcR-B1, Sox14, and Mical expression at WP stage. Quantification of immunostaining for EcR-B1, Sox14, and Mical levels was performed as described in Materials and Methods. Graphs display the average values of ddaC/ddaE ratios. Error bars represent S.E.M. n is shown on the bars. n.s., not significant. See genotypes in Text S1. (TIF) [file pbio.1001657.s021.tif]

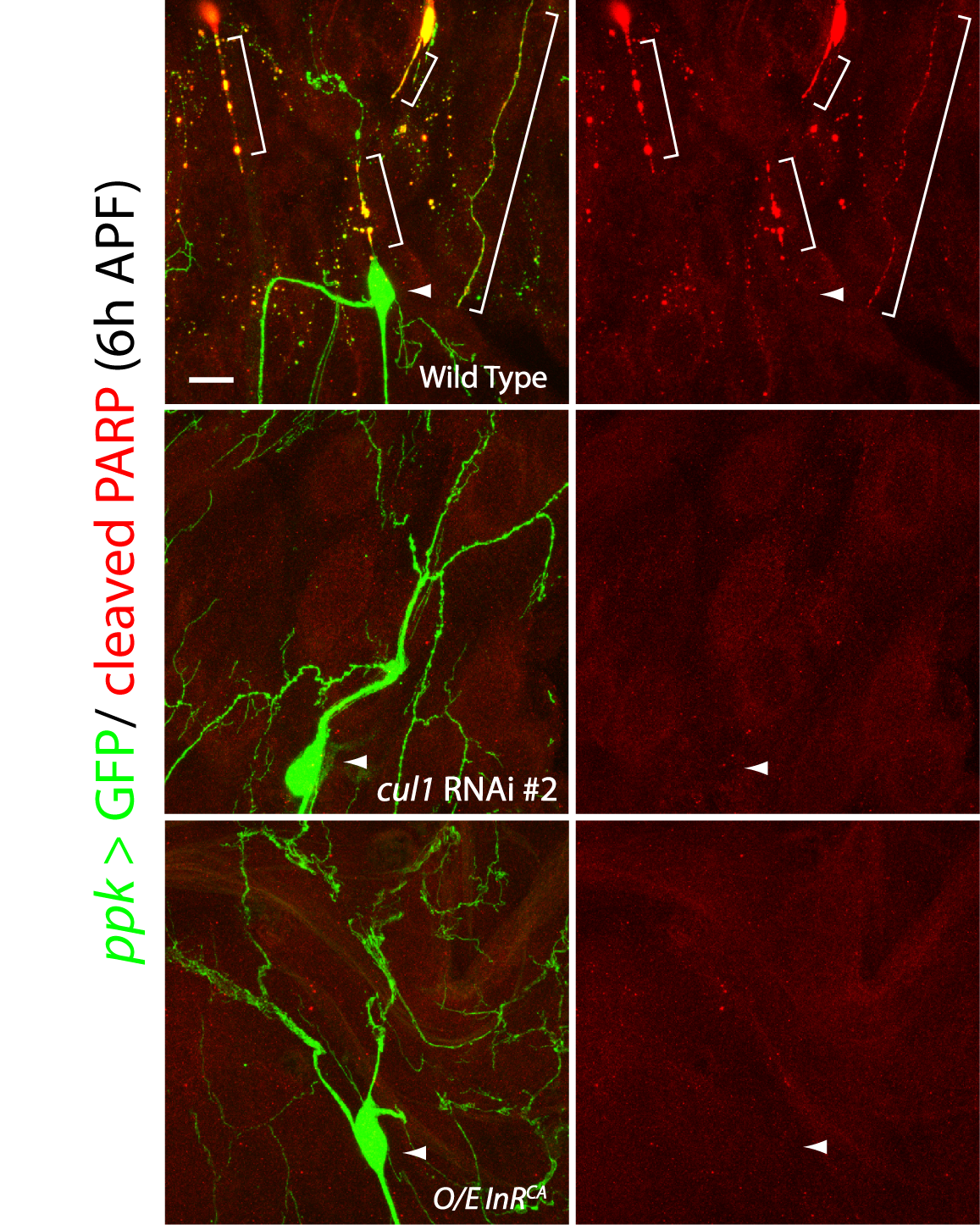

Supplement: Figure S22 — The SCF ligase and the InR/PI3K/TOR pathway regulate dendrite pruning in ddaC neurons at least in part by promoting local caspase activation in the dendrites. Confocal images of ddaC neurons expressing the caspase reporter construct CD8::PARP::VENUS at 6 h APF. While cleaved PARP marked in white bracket was readily detected in the wild-type ddaC neurons at 6 h APF, ddaC neurons expressing cul1 RNAi or InRCA failed to activate the caspase activity at 6 h APF. White arrowhead points to ddaC somas. The scale bar is 20 µm. See genotypes in Text S1. (TIF) [file pbio.1001657.s022.tif]
