# Supplementary material for: A Cullin1-Based SCF E3 Ubiquitin Ligase Targets the InR/PI3K/TOR Pathway to Regulate Neuronal Pruning
Source: PLoS Biol. 2013 Sep 17;11(9):e1001657. doi: 10.1371/journal.pbio.1001657 (PMC3775723; doi:10.1371/journal.pbio.1001657)
Supplement: Text S1 — List of fly strains. Various genotypes were used in the main and supplementary figures. (DOCX) [file pbio.1001657.s023.docx]

**List of fly strains**

**Figure 1:** (B, B’) *w*; ppk-Gal4, UAS-mCD8GFP/ ppk-Gal4, UAS-mCD8GFP; UAS-Dcr2/ UAS-Dcr2.* (C, C’) *w*; ppk-Gal4, UAS-mCD8GFP, UAS-Dcr2/ ppk-Gal4, UAS-mCD8GFP, UAS-Dcr2; UAS-cul1 RNAi #2/ UAS-cul1 RNAi #2.* (D, D’) *w*; ppk-Gal4 / ppk-Gal4, UAS-mCD8GFP; ppk-Gal4, UAS-mCD8GFP/ UAS-cul1^DN^.* (E, E’) *Gal4^5-40^, UAS-Venus:pm, SOP-flp #42/Y; FRTG13, cul1^EX^ /FRTG13, tubP-Gal80.* (F, F’) *Gal4^5-40^, UAS-Venus:pm, SOP-flp #42/Y; FRTG13, cul1^EX^ /FRTG13, tubP-Gal80, UAS-Flag-cul1/+.*

**Figure 2:** (B, B’ and G) *FRT19A/tubP-Gal80, hsFlp, FRT 19A; 201Y-Gal4, UAS mCD8GFP/+.* (C, C’and H) *w*, UAS-mCD8GFP, hsFlp/+; FRTG13, cul1^EX^, 201Y-Gal4/FRTG13, tubP-Gal80.* (D, D’) *roc1a^G1^, FRT19A/tubP-Gal80, hsFlp, FRT19A; 201Y-Gal4, UAS mCD8GFP/+.* (E, E’) *skpA^1^, FRT19A / tubP-Gal80, hsFlp, FRT 19A; 201Y-Gal4, UAS mCD8GFP/+.* (F, F’) *w*, hsFlp/+; 201Y-Gal4, UAS-mCD8GFP/+; FRT82B, slimb^2^/FRT82B, tubP-Gal80.*

**Figure 3:** (A, A’) *w*; ppk-Gal4, UAS-mCD8GFP/ ppk-Gal4, UAS-mCD8GFP; UAS-Dcr2/ UAS-Dcr2.* (B, B’) *roc1a^G1^, FRT19A/tubP-Gal80, hsFlp, FRT19A; Gal4^109(2)80^, UAS- mCD8GFP/+.* (C, C’) *w*; ppk-Gal4, UAS-mCD8GFP/+; UAS-skpA RNAi #1/UAS-Dcr2.* (D, D’) *w*; ppk-Gal4, UAS-mCD8GFP, UAS-Dcr2/ppk-Gal4, UAS-mCD8GFP, UAS-Dcr2; UAS-skpA RNAi #2/UAS-skpA RNAi #2.* (E, E’) *Gal4^5-40^, UAS-Venus:pm ,SOP-flp #42/Y;; FRT82B, slimb^8^/FRT82B, tubP-Gal80.*

**Figure 4:** (C) *w*;;Tub-myc slimb/Tub-myc slimb.* (D) *w*; 201Y-Gal4, UAS-mCD8GFP/201Y-Gal4, UAS-mCD8GFP.* (E) **WT:** *w*; 201Y-Gal4, UAS-mCD8GFP/201Y-Gal4, UAS-mCD8GFP.* ***EcR^DN^***: *w*; UAS-EcR^DN^/201Y-Gal4; UAS mCD8GFP/+.* ***sox14^Δ13^*:** *w*; 201Y-Gal4, UAS mCD8GFP, sox14^Δ13^/201Y-Gal4, UAS mCD8GFP, sox14^Δ13^.*

**Figure 5:** (A) *y,w.* (B) *w*; ppk-Gal4, UAS-mCD8GFP/+ ; UAS-Dcr2/ UAS-cul1 RNAi #2.* (C) *roc1a^G1^, FRT19A/tubP-Gal80, hsFlp, FRT19A; Gal4^109(2)80^, UAS- mCD8GFP/+.* (D) *w*; ppk-Gal4, UAS-mCD8GFP/UAS-skpA RNAi #3; UAS-Dcr2/ +.* (F, F’) *Gal4^5-40^, UAS-Venus:pm, SOP-flp #42/Y; FRTG13, sox14^Δ13^ /FRTG13, tubP-Gal80.* (G, G’) *Gal4^5-40^, UAS-Venus:pm, SOP-flp #42/Y; FRTG13, sox14^Δ13^, cul1^EX^ /FRTG13, tubP-Gal80*. (H, H’) *Gal4^5-40^, UAS-Venus:pm, SOP-flp #42/Y;; FRT82B, mical^15253^ /FRT82B, tubP-Gal80.* (I, I’) *Gal4^5-40^, UAS-Venus:pm, SOP-flp #42/Y;; FRT82B, slimb^8^, mical^15253^ /FRT82B, tubP-Gal80.*

**Figure 6:** (A) *w*; ppk-Gal4, UAS-mCD8GFP, UAS-Dcr2/ +; UAS-cul1 RNAi #2/ UAS-mical^N-ter^.* (B) *w*; ppk-Gal4, UAS-mCD8GFP, UAS-Dcr2/ UAS-Ci^Cell^; UAS-cul1 RNAi #2/+.* (C) *w*; ppk-Gal4, UAS-mCD8GFP, UAS-Dcr2/ +; UAS-cul1 RNAi #2/ UAS-sgg^S9A^.* (D) *UAS-InR^DN^/+; ppk-Gal4, UAS-mCD8GFP, UAS-Dcr2/ +; UAS-cul1 RNAi #2/+.* (E) *w*; ppk-Gal4, UAS-mCD8GFP, UAS-Dcr2/ UAS-Notch^DN^; UAS-cul1 RNAi #2/+.* (F) *w*; ppk-Gal4,UAS-mCD8GFP,UAS-Dcr2/ +; UAS-skpA RNAi #2 / UAS-mical^N-ter^.* (G) *UAS-InR^DN^/+; ppk-Gal4,UAS-mCD8GFP,UAS-Dcr2/ +; UAS-skpA RNAi #2/ +.* (H) *w*; ppk-Gal4,UAS-mCD8GFP,UAS-Dcr2 / UAS-InR^CA^; UAS-skpA RNAi #2 / +.*

**Figure 7:** (A) *w*; ppk-Gal4, UAS-mCD8GFP, UAS-Dcr2/ +; UAS-cul1 RNAi #2/ UAS-mical^N-ter^.* (B) *w*; ppk-Gal4, UAS-mCD8GFP, UAS-Dcr2/ UAS-PI3K^DN^; UAS-cul1 RNAi #2/ +.* (C) *w*; ppk-Gal4, UAS-mCD8GFP, UAS-Dcr2/ UAS-PTEN; UAS-cul1 RNAi #2/ +.* (D) *w*; ppk-Gal4, UAS-mCD8GFP, UAS-Dcr2/ +; UAS-cul1 RNAi #2/ UAS-akt RNAi #1.* (E) *w*; ppk-Gal4, UAS-mCD8GFP, UAS-Dcr2/ UAS-TSC1, UAS-TSC2; UAS-cul1 RNAi #2/ +.* (F) *w*; ppk-Gal4, UAS-mCD8GFP, UAS-Dcr2/ UAS-TOR^TED^; UAS-cul1 RNAi #2/ +.* (G) *w*; ppk-Gal4, UAS-mCD8GFP, UAS-Dcr2/ +; UAS-cul1 RNAi #2/ UAS-4E-BP(AA).* (H) *w*; ppk-Gal4, UAS-mCD8GFP, UAS-Dcr2/ UAS-S6K^KQ^; UAS-cul1 RNAi #2/ +.* (I, I’) *Gal4^5-40^, UAS-Venus:pm, SOP-flp #42/Y; FRTG13, cul1^EX^/FRTG13, tubP-Gal80; UAS-InR^DN^/+.* (J, J’) *Gal4^5-40^, UAS-Venus:pm, SOP-flp #42/Y; FRTG13, cul1^EX^/FRTG13, tubP-Gal80; UAS-4E-BP(AA)/+.*

**Figure 8:** (A) *w*; ppk-Gal4, UAS-mCD8GFP/ ppk-Gal4, UAS-mCD8GFP; UAS-Dcr2/ UAS-Dcr2; UAS-ctrl RNAi/ UAS-ctrl RNAi.* (B) *w*; ppk-Gal4, UAS-mCD8GFP, UAS-Dcr2/ ppk-Gal4, UAS-mCD8GFP, UAS-Dcr2; UAS-cul1 RNAi #2/ UAS-cul1 RNAi #2.* (C) *w*; ppk-Gal4, UAS-mCD8GFP, UAS-Dcr2/ ppk-Gal4, UAS-mCD8GFP, UAS-Dcr2; UAS-akt RNAi BL31701/ UAS-akt RNAi BL31701***.** (E-E’) *w*; UAS-akt/ppk-Gal4, UAS-mCD8GFP, UAS-Dcr2; UAS-ctrl RNAi/+*. (F-F’) *w*;UAS-akt/ ppk-Gal4, UAS-mCD8GFP, UAS-Dcr2; UAS-cul1 RNAi #2/+*. (H) **Ctrl:** *elav-Gal4;;UAS-Dicer2/+.* ***cul1* RNAi #1:** *elav-Gal4/+; UAS-cul1 RNAi #1/UAS-Dicer2.* (J) *elav-Gal4/+; UAS-akt/+ ; tub-myc-slimb/+.*

**Figure 9:** (A, A’) *w*; ppk-Gal4/ppk-Gal4; ppk-Gal4, UAS-mCD8GFP/ppk-Gal4, UAS-mCD8GFP.* (B, B’) *w*; ppk-Gal4/UAS-InR^CA^; ppk-Gal4, UAS-mCD8GFP/+.* (C, C’) *UAS-PI3K^CA^, w*/ +; ppk-Gal4 / +; ppk-Gal4, UAS-mCD8GFP/ +.* (D, D’) *Gal4^5-40^, UAS-Venus:pm, SOP-flp #42 /Y; pten^C494^, FRT40A/tubP-Gal80, FRT40A.* (E) *w*; ppk-Gal4, UAS-mCD8GFP/+; mical^15256^/Df(3R)swp2^MICAL^*. (F)*w*; ppk-Gal4,UAS-mCD8GFP/ UAS-InR^CA^; mical^15256^/ Df(3R) swp2^MICAL^*. (G) *w*; ppk-Gal4,UAS-mCD8GFP/UAS-PI3K^WT^; mical^15256^/ Df(3R)swp2^MICAL^*. (H) *w*; ppk-Gal4, UAS-mCD8GFP/UAS-Rheb; mical^15256^/ Df(3R)swp2^MICAL^*.

**Figure S1.** (A) ***cul1* RNAi #1:** *w*; ppk-Gal4, UAS-mCD8GFP / UAS-cul1 RNAi #1; UAS-Dcr2/+.* ***cul1* RNAi #2:** *w*; ppk-Gal4, UAS-mCD8GFP / +; UAS-Dcr2/ UAS-cul1 RNAi #2.* (B, C, D) **wild-type*:*** *Gal4^5-40^, UAS-Venus:pm, SOP-flp #42/Y; FRTG13/tubP-Gal80 FRTG13.* ***cul1^EX^:*** *Gal4^5-40^, UAS-Venus:pm , SOP-flp #42/Y; FRTG13, cul1^EX^/FRTG13, tubP-Gal80.* ***nedd8^AN015^:*** *Gal4^5-40^, UAS-Venus:pm, SOP-flp #42/Y; nedd8^AN015^, FRT40A/tubP-Gal80, FRT40A.*

**Figure S2:** (A) **ctrl RNAi:** *w*; ppk-Gal4, UAS-mCD8GFP, UAS-Dcr2/ +; ctrl RNAi/+.* ***cul1 RNAi #2*:** *w*; ppk-Gal4, UAS-mCD8GFP, UAS-Dcr2/ + ; UAS-cul1 RNAi #2/ +.* ***skpA RNAi #2*:** *w*; ppk-Gal4, UAS-mCD8GFP, UAS-Dcr2/ +; UAS-skpA RNAi #2/ +.* ***roc1a RNAi #2*:** *w*; ppk-Gal4, UAS-mCD8GFP, UAS-Dcr2/ + ; UAS-roc1A RNAi #2/ +.* (B) *w*; ppk-CD4-tdTomato /+ ; elav-GeneSwitch-Gal4/+. w*; ppk-CD4-tdTomato /+ ; elav-GeneSwitch-Gal4/ UAS-cul1^DN^.*

**Figure S3.** ***roc1a* RNAi #1:** *w*; ppk-Gal4, UAS-mCD8GFP / UAS-roc1a RNAi #1; UAS-Dcr2/+.* **2*ppk* x *roc1a* RNAi #2**: *w*; ppk-Gal4, UAS-mCD8GFP, UAS-Dcr2/ppk-Gal4, UAS-mCD8GFP, UAS-Dcr2; UAS-roc1a RNAi #2/ UAS-roc1a RNAi #2.* ***slimb* RNAi #1:** *w*; ppk-Gal4, UAS-mCD8GFP/UAS-slimb RNAi #1; UAS-Dcr2/+.* ***slimb* RNAi #2:** *w*; ppk-Gal4, UAS-mCD8GFP/UAS-slimb RNAi #2; UAS-Dcr2/+.*

**Figure S4.** (A) **wild-type:** *y,w; Gal4^2-21^, UAS-mCD8GFP.* ***roc1a^G1^****: roc1a^G1^, FRT19A/ tubP-Gal80, hsFlp, FRT 19A; Gal4^109(2)80^, UAS mCD8GFP/+.* ***skpA* RNAi #3:** *w*; UAS-Dcr2/UAS-skpA RNAi #3; Gal4^2-21^, UAS-mCD8GFP/+.* ***slimb^8^:*** *Gal4^5-40^, UAS-Venus:pm,SOP-flp #42/Y;; FRT82B, slimb^8^/FRT82B, tubP-Gal80.* (B) **wild-type (left):** *FRT19A/ tubP-Gal80, hsFlp, FRT 19A; Gal4^109(2)80^, UAS-mCD8GFP/+.* ***roc1a^G1^:*** *roc1a^G1^, FRT19A/ tubP-Gal80, hsFlp, FRT 19A; Gal4^109(2)80^, UAS mCD8GFP/+.* ***slimb^8^:*** *Gal4^5-40^,UAS-Venus:pm ,SOP-flp #42/Y;; FRT82B, slimb^8^/FRT82B, tubP-Gal80.* **wild-type (right):** *w*;Gal4^109(2)^80, UAS-mCD8GFP/+; UAS-Dcr2/+.* ***skpA* RNAi #3:** *w*; Gal4^109(2)80^, UAS-mCD8GFP/UAS-skpA RNAi #3; UAS-Dcr2/+.*

**Figure S5: *roc1b^dc3^*:** *w*; ppk-Gal4, UAS-mCD8GFP/ppk-Gal4, UAS-mCD8GFP; roc1b^dc3^/roc1b^dc3^.* ***cul3^gft2^:*** *Gal4^5-40^, UAS-Venus:pm, SOP-flp #42/Y; cul3^gft2^, FRT40A/tubP-Gal80, FRT40A.* ***ago^3^:*** *Gal4^5-40^, UAS-Venus:pm, SOP-flp #42/Y;; ago^3^, FRT80B/tubP-Gal80, FRT80B.* ***cul4^11L^:*** *Gal4^5-40^, UAS-Venus:pm, SOP-flp #42/Y; cul4^11L^, FRTG13/tubP-Gal80, FRTG13.*

**Figure S6:** (A) **wild-type:** *FRT19A/tubP-Gal80, hsFlp, FRT 19A; 201Y-Gal4, UAS mCD8GFP/+.* ***cul1^EX^* Rescue*:*** *w*, UAS-mCD8GFP, hsFlp/+; FRTG13, cul1^EX^, 201Y-Gal4/FRTG13, tubP-Gal80; UAS-Flag-cul1/+.* ***roc1a^G1^* Rescue**: *roc1a^G1^, FRT19A/tubP-Gal80, hsFlp, FRT 19A; 201Y-Gal4, UAS mCD8GFP/UAS-roc1a.* (B) **wild-type:** w**; 201Y-Gal4, UAS-mCD8GFP* ***. roc1b^dc3^*:** w**; 201Y-Gal4, UAS-mCD8GFP/+; roc1b^dc3^/ roc1b^dc3^.* (C) **Wild type:** *FRT19A/tubP-Gal80, hsFlp, FRT 19A; 201Y-Gal4, UAS mCD8GFP/+.* ***cul1^EX^:*** *w*, UAS-mCD8GFP, hsFlp/+; FRTG13, cul1^EX^, 201Y-Gal4/FRTG13, tubP-Gal80.* ***skpA^1^:*** *skpA^1^, FRT19A / tubP-Gal80, hsFlp, FRT 19A; 201Y-Gal4, UAS mCD8GFP/+.*

**Figure S7:** (A) **wild-type:** *w*; ppk-Gal4, UAS-mCD8GFP, UAS-Dcr2/+; mical-lacZ /UAS-control RNAi.* ***cul RNAi #2:*** *w*; ppk-Gal4, UAS-mCD8GFP, UAS-Dcr2/+; mical-lacZ /UAS-cul1 RNAi #2.* ***skpA RNAi #3:*** *w*; ppk-Gal4, UAS-mCD8GFP, UAS-Dcr2/UAS-skpA RNAi #3; mical-lacZ /+.* (B) **wild-type:** *y, w*. ***cul1^EX^:*** *Gal4^5-40^, UAS-Venus:pm, SOP-flp #42/Y; FRTG13, cul1^EX^/FRTG13, tubP-Gal80.* ***roc1a^G1^****: roc1a^G1^, FRT19A /tubP-Gal80, hsFlp, FRT 19A; Gal4^109(2)80^, UAS mCD8GFP/+.* ***skpA RNAi #3:*** *w*; ppk-Gal4, UAS-mCD8GFP/UAS-skpA RNAi #3; UAS-Dcr2/+.* ***slimb^8^***: *Gal4^5-40^, UAS-Venus:pm, SOP-flp #42/Y;; FRT82B, slimb^8^/FRT82B, tubP-Gal80.* ***nedd8^AN015^***: *Gal4^5-40^, UAS-Venus:pm, SOP-flp #42/Y; nedd8^AN015^, FRT40A/tubP-Gal80, FRT40A.*

**Figure S8 and S9: *mical*:** *w*; ppk-Gal4, UAS-mCD8GFP, UAS-Dcr2/+; Mical^15256^/ Df(3R)swp2^MICAL^*. ***mical*, *cul1* RNAi:** *w*; ppk-Gal4, UAS-mCD8GFP, UAS-Dcr2/UAS-cul1 RNAi #1; Mical^15256^/ Df(3R)swp2^MICAL^.* ***mical, roc1a* RNAi:** *w*; ppk-Gal4, UAS-mCD8GFP, UAS-Dcr2/UAS-roc1a RNAi #1; Mical^15256^/Df(3R)swp2^MICAL^.* ***cul1 RNAi #2*:** *w*;; UAS-cul1 RNAi #2/ppk-Gal4, UAS-mCD8GFP, UAS-Dcr2.* ***sox14*:** *w*; sox14^Δ13^/sox14^Δ13^; ppk-Gal4, UAS-mCD8GFP, UAS-Dcr2/ ppk-Gal4, UAS-mCD8GFP, UAS-Dcr2.* ***sox14*, *cul1 RNAi #2*:** *w*; sox14^Δ13^/sox14^Δ13^; UAS-cul1 RNAi #2/ppk-Gal4, UAS-mCD8GFP, UAS-Dcr2.*

**Figure S10:** (A) ***2ppk Ci^Cell^*:** *w*; ppk-Gal4/UAS-Ci^Cell^; ppk-Gal4, UAS-mCD8GFP/+.* ***2ppk Sgg^S9A^*:** *w*; ppk-Gal4/+; ppk-Gal4, UAS-mCD8GFP/UAS-Sgg^S9A^.* ***2ppk InR^DN^*:** *UAS-InR^DN^/+; ppk-Gal4/ +; ppk-Gal4, UAS-mCD8GFP/+.* ***2ppk Notch^DN^*:** *w*; ppk-Gal4/UAS-Notch^DN^; ppk-Gal4, UAS-mCD8GFP/+.* ***2ppk Ci^U^*:** *w*; ppk-Gal4/+; ppk-Gal4, UAS-mCD8GFP/UAS-Ci^U^.* ***2ppk Arm^S10^*:** *w*; ppk-Gal4/+; ppk-Gal4, UAS-mCD8GFP/UAS-Arm^S10^.* ***2ppk InR^CA^*:** *w*; ppk-Gal4/UAS-InR^CA^; ppk-Gal4, UAS-mCD8GFP/+.* ***2ppk Notch^CA^*:***w*; ppk-Gal4/+; ppk-Gal4, UAS-mCD8GFP/UAS-Notch^CA^.*

**Figure S11: *cul1^DN^, mical^N-Ter^:*** *w*; ppk-Gal4, UAS-mCD8GFP / +; UAS-cul1^DN^/ UAS-mical^N-ter^.* ***cul1^DN^, Ci^U^ :****w*; ppk-Gal4, UAS-mCD8GFP /+; UAS-cul1^DN^/ UAS-Ci^U^.* ***cul1^DN^, Arm^S10^:*** *w*; ppk-Gal4, UAS-mCD8GFP / +; UAS-cul1^DN^/ UAS-Arm^S10^.* ***cul1^DN^, InR^CA^:*** *w*; ppk-Gal4, UAS-mCD8GFP / UAS-InR^CA^; UAS-cul1^DN^/ +.* ***cul1^DN^, Notch^CA^:*** *w*; ppk-Gal4, UAS-mCD8GFP / +; UAS-Cul1 ^DN^/ UAS-Notch^CA^.*

**Figure S12: (**A) ***cul1* RNAi***,* ***mical^N-Ter^*:** *w*; ppk-Gal4, UAS-mCD8GFP, UAS-Dcr2/+; UAS-cul1 RNAi #2/UAS-mical^N-Ter^.* ***cul1* RNAi***,* ***Bsk^DN^*:** *w*; ppk-Gal4, UAS-mCD8GFP, UAS-Dcr2/+; UAS-cul1 RNAi #2/UAS-Bsk^DN^.* ***cul1* RNAi***,* ***Fos^DN^*:** *w*; ppk-Gal4, UAS-mCD8GFP, UAS-Dcr2/UAS-Fos^DN^; UAS-cul1 RNAi #2/+.* ***cul1* RNAi***,* ***dome^ΔCYT^*:** *w*; ppk-Gal4, UAS-mCD8GFP, UAS-Dcr2/UAS-Dome^ΔCYT^; UAS-cul1 RNAi #2/+.* ***cul1* RNAi***,* ***yki^S168A^*:** *w*; ppk-Gal4, UAS-mCD8GFP, UAS-Dcr2/+; UAS-cul1 RNAi #2/UAS-yki^S168A^.* ***cul1* RNAi***,* ***EGFR^DN^*:** *w*; ppk-Gal4, UAS-mCD8GFP, UAS-Dcr2/UAS-EGFR^DN^; UAS-cul1 RNAi #2/+.* ***cul1* RNAi***,* ***Pvr^DN^*:** *w*; ppk-Gal4, UAS-mCD8GFP, UAS-Dcr2/UAS-Pvr^DN^; UAS-cul1 RNAi #2/+.* ***cul1* RNAi***,* ***tkv^DN^*:** *w*; ppk-Gal4, UAS-mCD8GFP, UAS-Dcr2/+; UAS-cul1 RNAi #2/UAS-tkv^DN^.* (B) ***cul1* RNAi, *mical^N-ter^*:** *w*; ppk-Gal4, UAS-mCD8GFP, UAS-Dcr2/ +; UAS-cul1 RNAi #2/ UAS-mical^N-ter^.* ***cul1* RNAi, *Ci^Cell^*:** *w*; ppk-Gal4, UAS-mCD8GFP, UAS-Dcr2/ UAS-Ci^Cell^; UAS-cul1 RNAi #2/+.* ***cul1* RNAi, *sgg^S9A^*:** *w*; ppk-Gal4, UAS-mCD8GFP, UAS-Dcr2/ +; UAS-cul1 RNAi #2/ UAS-sgg^S9A^.* ***cul1* RNAi, *InR^DN^*:** *UAS-InR^DN^/+; ppk-Gal4, UAS-mCD8GFP, UAS-Dcr2/ +; UAS-cul1 RNAi #2/+.* ***cul1* RNAi, *Notch^DN^*:***w*; ppk-Gal4, UAS-mCD8GFP, UAS-Dcr2/ UAS-Notch^DN^; UAS-cul1 RNAi #2/+.* ***skpA* RNAi, *mical^N-ter^*:***w*; ppk-Gal4,UAS-mCD8GFP,UAS-Dcr2/ +; UAS-skpA RNAi #2 / UAS-mical^N-ter^.* ***skpA* RNAi, *InR^DN^*:** *UAS-InR^DN^/+; ppk-Gal4,UAS-mCD8GFP,UAS-Dcr2/ +; UAS-skpA RNAi #2/ +.* ***skpA* RNAi, *InR^CA^*:** *w*; ppk-Gal4,UAS-mCD8GFP,UAS-Dcr2 / UAS-InR^CA^; UAS-skpA RNAi #2 / +.*

**Figure S13:** (A) ***cul1^DN^,mical^N-ter^:*** *w*; ppk-Gal4, UAS-mCD8GFP / +; UAS-cul1^DN^/ UAS-mical^N-ter^.* ***cul1^DN^,PI3K^DN^:*** *w*; ppk-Gal4, UAS-mCD8GFP / UAS-PI3K^DN^; UAS-cul1^DN^/ +.* ***cul1^DN^,PTEN:*** *w*; ppk-Gal4, UAS-mCD8GFP / UAS-PTEN; UAS-cul1^DN^/+.* ***cul1^DN^,InR^DN^:*** *UAS-InR^DN^/+; ppk-Gal4, UAS-mCD8GFP /+ ; UAS-cul1^DN^/ +.* ***cul1^DN^,TSC1,TSC2:*** *w*; ppk-Gal4, UAS-mCD8GFP / UAS-TSC1, UAS-TSC2 ; UAS-cul1^DN^/ +.* ***cul1^DN^,Tor^TED^:*** *w*; ppk-Gal4, UAS-mCD8GFP / UAS-TOR^TED^; UAS-cul1^DN^/ +.* ***cul1^DN^,4E-BP(AA):*** *w*; ppk-Gal4, UAS-mCD8GFP / + ; UAS-cul1^DN^/ UAS-4E-BP(AA).* ***cul1^DN^, S6K^KQ^:*** *w*; ppk-Gal4, UAS-mCD8GFP / UAS-S6K^KQ^; UAS-cul1^DN^/ +.*

**Figure S14: Wild Type:** *w*; ppk-Gal4, UAS-mCD8GFP, UAS-Dcr2/ ppk-Gal4, UAS-mCD8GFP, UAS-Dcr2.* ***cul1 RNAi #2****:* *w*; ppk-Gal4, UAS-mCD8GFP, UAS-Dcr2/ ppk-Gal4, UAS-mCD8GFP, UAS-Dcr2; UAS-cul1 RNAi #2/ UAS-cul1 RNAi #2.* ***mical RNAi****:* *w*; ppk-Gal4, UAS-mCD8GFP, UAS-Dcr2/ppk-Gal4, UAS-mCD8GFP, UAS-Dcr2; UAS-mical RNAi/ UAS-mical RNAi.*

**Figure S15:** (A) ***cul1* RNAi***,****mical^N-ter^*:** *w*; ppk-Gal4, UAS-mCD8GFP, UAS-Dcr2/+; UAS-cul1 RNAi #2/UAS-mical^N-ter^.* ***cul1* RNAi***,****InR^DN^*:** *UAS-InR^DN^/+; ppk-Gal4, UAS-mCD8GFP, UAS-Dcr2/+; UAS-cul1 RNAi #2/+.* ***cul1* RNAi***,****PI3K^DN^*:** *w*; ppk-Gal4, UAS-mCD8GFP, UAS-Dcr2/UAS-PI3K^DN^; UAS-cul1 RNAi #2/+.* ***cul1* RNAi***,****PTEN*:** *w*; ppk-Gal4, UAS-mCD8GFP, UAS-Dcr2/UAS-PTEN; UAS-cul1 RNAi #2/+.* ***cul1* RNAi***,****TOR^TED^*:** *w*; ppk-Gal4,UAS-mCD8GFP, UAS-Dcr2/UAS-TOR^TED^; UAS-cul1 RNAi #2/+.* ***cul1* RNAi*,TSC1,TSC2*:** *w*; ppk-Gal4,UAS-mCD8GFP, UAS-Dcr2/UAS-TSC1,UAS-TSC2; UAS-cul1 RNAi #2/+.* ***cul1* RNAi***,****S6K^KQ^*:** *w*; ppk-Gal4,UAS-mCD8GFP,UAS-Dcr2/UAS-S6K^KQ^; UAS-cul1 RNAi #2/+.* ***cul1* RNAi***,****4E-BP(AA)*:** *w*; ppk-Gal4, UAS-mCD8GFP, UAS-Dcr2/+; UAS-cul1 RNAi #2/UAS-4E-BP(AA).****cul1* RNAi***,****akt* RNAi:** *w*; ppk-Gal4, UAS-mCD8GFP, UAS-Dcr2/ +; UAS-cul1 RNAi #2/ UAS-akt RNAi #1.*  *(B)* ***cul1^DN^,mical^N-ter^:*** *w*; ppk-Gal4, UAS-mCD8GFP/+; UAS-cul1^DN^/UAS-mical^N-ter^.* ***cul1^DN^,InR^DN^:*** *UAS-InR^DN^/+; ppk-Gal4, UAS-mCD8GFP/+; UAS-cul1^DN^/+.* ***cul1^DN^,PI3K^DN^:*** *w*; ppk-Gal4,UAS-mCD8GFP/UAS-PI3K^DN^; UAS-cul1^DN^/+.* ***cul1^DN^,PTEN:*** *w*; ppk-Gal4, UAS-mCD8GFP/UAS-PTEN; UAS-cul1^DN^/+.* ***cul1^DN^,TOR^TED^:*** *w*; ppk-Gal4, UAS-mCD8GFP / UAS-TOR^TED^; UAS-cul1 ^DN^/+.* ***cul1^DN^,TSC1,TSC2:*** *w*; ppk-Gal4, UAS-mCD8GFP/UAS-TSC1, UAS-TSC2; UAS-cul1^DN^/+.* ***cul1 ^DN^,S6K^KQ^:*** *w*; ppk-Gal4, UAS-mCD8GFP/UAS-S6K^KQ^; UAS-cul1^DN^/+.* ***cul1^DN^****,****4E-BP(AA):*** *w*; ppk-Gal4, UAS-mCD8GFP/+ ; UAS-cul1^DN^/UAS-4E-BP(AA).*

**Figure S16:** (A) ***cul1* RNAi***,****mical^N-ter^*:** *w*; ppk-Gal4, UAS-mCD8GFP, UAS-Dcr2/+; UAS-cul1 RNAi #2/UAS-mical^N-ter^.* ***cul1* RNAi***,****InR^DN^*:** *UAS-InR^DN^/+; ppk-Gal4, UAS-mCD8GFP, UAS-Dcr2/+; UAS-cul1 RNAi #2/+.* ***cul1* RNAi***,****PI3K^DN^*:** *w*; ppk-Gal4, UAS-mCD8GFP, UAS-Dcr2/UAS-PI3K^DN^; UAS-cul1 RNAi #2/+.* ***cul1* RNAi***,****PTEN*:** *w*; ppk-Gal4, UAS-mCD8GFP, UAS-Dcr2/UAS-PTEN; UAS-cul1 RNAi #2/+.* ***cul1* RNAi***,****TOR^TED^*:** *w*; ppk-Gal4,UAS-mCD8GFP, UAS-Dcr2/UAS-TOR^TED^; UAS-cul1 RNAi #2/+.* ***cul1* RNAi*,TSC1,TSC2*:** *w*; ppk-Gal4,UAS-mCD8GFP, UAS-Dcr2/UAS-TSC1,UAS-TSC2; UAS-cul1 RNAi #2/+.* ***Cul1* RNAi***,****S6K^KQ^*:** *w*; ppk-Gal4,UAS-mCD8GFP,UAS-Dcr2/UAS-S6K^KQ^; UAS-cul1 RNAi #2/+.* ***cul1* RNAi***,****4E-BP(AA)*:** *w*; ppk-Gal4, UAS-mCD8GFP, UAS-Dcr2/+; UAS-Cul1 RNAi #2/UAS-4E-BP(AA).* (B) ***mical,mical^N-ter^*:** *w*; ppk-Gal4, UAS-mCD8GFP/UAS-mical^N-ter^; mical^15256^/Df(3R)swp2^MICAL^*. ***mical,InR^DN^*:** *w*; ppk-Gal4, UAS-mCD8GFP/UAS-InR^DN^; mical^15256^/Df(3R)swp2^MICAL^*. ***mical,PI3K^DN^*:** *w*; ppk-Gal4, UAS-mCD8GFP/UAS-PI3K^DN^; mical^15256^/Df(3R)swp2^MICAL^*. ***mical,PTEN*:** *w*; ppk-Gal4, UAS-mCD8GFP/UAS-PTEN; mical^15256^/Df(3R)swp2^MICAL^*. ***mical,TOR^TED^*:** *w*; ppk-Gal4, UAS-mCD8GFP/UAS-TOR^TED^; mical ^15256^/Df(3R)swp2^MICAL^*. ***mical,TSC1,TSC2*:** *w*; ppk-Gal4, UAS-mCD8GFP/UAS-TSC1,UAS-TSC2; mical^15256^/Df(3R)swp2^MICAL^*. ***mical,S6K^KQ^*:** *w*; ppk-Gal4, UAS-mCD8GFP/UAS-S6K^KQ^; mical^15256^/Df(3R)swp2^MICAL^*. ***mical,4E-BP(AA)*:** *w*; ppk-Gal4, UAS-mCD8GFP/UAS-4E-BP(AA); mical^15256^/ Df(3R)swp2^MICAL^*.

**Figure S17: O/E skpA-RFP:** *w*;ppk-Gal4, UAS-mCD8GFP/+; UAS-skpA-RFP/+.* ***O/E*** **akt:** *w*;UAS-akt/+ ; ppk-Gal4, UAS-mCD8GFP/+.*

**Figure S18:** (A)  **Ctrl:** *elav-Gal4; UAS-Dcr2.* ***cul1* RNAi #1:** *elav-Gal4/+; UAS-cul1 RNAi #1/UAS-Dcr2.*

**Figure S19:** (A) ***2ppk Rheb:*** *w*; ppk-Gal4/UAS-Rheb; ppk-Gal4, UAS-mCD8GFP/+.* ***2ppk S6K^STDETE^:*** *w*; ppk-Gal4 /+; ppk-Gal4, UAS-mCD8GFP/UAS-S6K^STDETE^.* (B) ***pten^1^*:** *Gal4^5-40^, UAS-Venus:pm, SOP-flp #42/Y; pten^1^, FRT40A/tubP-Gal80, FRT40A.* (C) ***ctrl* MARCM*:*** *w*, UAS-mCD8GFP, hsFlp/+; FRT40A /201Y-Gal4, FRT40A, tubP-Gal80.* ***pten^c494^* MARCM*:*** *w*, UAS-mCD8GFP, hsFlp/+; FRT40A, pten^c494^ /201Y-Gal4, FRT40A, tubP-Gal80.* **Wild Type:** *w*; 201Y-Gal4, UAS-mCD8GFP/201Y-Gal4, UAS-mCD8GFP.* ***InR^CA^*:** *w*; 201Y-Gal4, UAS-mCD8GFP/UAS InR^CA^.* (D) ***InR^CA^*;*ctrl* RNAi:** w*;*UAS-InR^CA^*/+; *UAS-ctrl* RNAi/ *ppk-Gal4, UAS-mCD8GFP, UAS-Dcr2.* ***InR^CA^*;*akt* RNAi:** w*;*UAS-InR^CA^*/+; *UAS-akt* RNAi (BL31701) / *ppk-Gal4, UAS-mCD8GFP, UAS-Dcr2.*

**Figure S20: *cul1^DN^,mical^N-ter^:*** *w*; ppk-Gal4,UAS-mCD8GFP/+; UAS-cul1^DN^/UAS-mical^N-ter^.* ***cul1 ^DN^,InR^CA^:*** *w*; ppk-Gal4, UAS-mCD8GFP/UAS-InR^CA^; UAS-cul1^DN^/+.* ***cul1 ^DN^,PI3K^CA^:*** *UAS-PI3K^CA^,w*/+; ppk-Gal4, UAS-mCD8GFP/+; UAS-cul1^DN^/+.* ***cul1^DN^,Rheb:*** *w*; ppk-Gal4, UAS-mCD8GFP/UAS-Rheb; UAS-cul1^DN^/+.* ***cul1^DN^,S6K^STDETE^:*** *w*; ppk-Gal4,UAS-mCD8GFP/+; UAS-cul1^DN^/UAS-S6K^STDETE^.*

**Figure S21:** (A) **Wild Type:** *w*; ppk-Gal4/ppk-Gal4; ppk-Gal4, UAS-mCD8GFP/ppk-Gal4, UAS-mCD8GFP.* ***InR^CA^:*** *w*; ppk-Gal4/UAS-InR^CA^; ppk-Gal4, UAS-mCD8GFP/+.* ***PI3K^CA^:*** *UAS-PI3K^CA^, w*/+; ppk-Gal4/+; ppk-Gal4, UAS-mCD8GFP/+.* (B) **wild-type:** *y,w*. ***pten^C494^*:** *Gal4^5-40^, UAS-Venus:pm, SOP-flp #42/Y; pten^c494^, FRT40A/tubP-Gal80, FRT40A.* ***pten^1^*:** *Gal4^5-40^, UAS-Venus:pm, SOP-flp #42/Y; pten^1^, FRT40A/tubP-Gal80, FRT40A.*

**Figure S22:** **Wild Type**: *w*;ppk-Gal4, UAS-mCD8GFP,UAS-Dcr2/UAS-CD8::PARP-Venus; UAS-ctrl RNAi/+.* ***cul1 RNAi #2***: *w*;ppk-Gal4, UAS-mCD8GFP,UAS-Dcr2/UAS-CD8::PARP-Venus; UAS-cul1 RNAi #2/ + .* ***InR^CA^***: *w*;UAS-InR^CA^/UAS-CD8::PARP-Venus; ppk-Gal4, UAS-mCD8GFP/+.*
